# A regioselective sustainable neat approach to naphtho[2,3-*b*]thiophene-4,9-dione: detailed mechanistic study and DFT analysis

**DOI:** 10.1039/d5ra08362a

**Published:** 2026-01-28

**Authors:** Soumen K. Manik, Sk Asraf Ali, Nayim Sepay, Aniruddha Deb, Manik Shit, Paritosh Barik, Jyotirmoy Rath, Kankan K. Maity, Sudhir C. Pal, Shubhankar Samanta, Nirmal K. Hazra

**Affiliations:** a Department of Chemistry, Coastal Environmental Studies Research Centre, Egra Sarada Shashi Bhusan College, Vidyasagar University Purba Medinipur Egra West Bengal 721429 India nirmalkrhazra@gmail.com; b Department of Chemistry, Bidhannagar College EB 2, Sector I, Salt Lake Kolkata-700064 West Bengal India chemshubha@gmail.com; c District Institute of Education and Training (DIET) Jhargram West Bengal-721507 India; d Department of Chemistry, Lady Brabourne College Kolkata-700017 West Bengal India; e Department of Chemical Engineering, Birla Institute of Technology Mesra Ranchi Jharkhand 835215 India; f Department of Chemistry, Jadavpur University Jadavpur Kolkata-700032 West Bengal India; g Department of Physics, Coastal Environmental Studies Research Centre, Egra Sarada Shashi Bhusan College, Vidyasagar University Purba Medinipur Egra West Bengal 721429 India; h Department of Chemistry, Belda College Paschim Medinipur Belda West Bengal-721424 India

## Abstract

An environment friendly simple, solvent-free, and lower *E*-factor technique has been developed for synthesizing anticancer property-containing substituted naphtho[2,3-*b*]thiophene-4,9-dione derivatives *via* a one-pot three-component reaction using active methylene compounds, alkyl/aryl isothiocyanate, and 1,4-naphthoquinone derivatives. The reaction proceeds through the regioselective Michael addition between the di-anionic intermediate and 1,4-naphthoquinone derivatives, followed by a domino reaction, which has been established *via* DFT study. Finally, it undergoes a Krapcho reaction followed by aromatization in a basic environment, yielding the desired product naphtho[2,3-*b*]thiophene-4,9-dione. The reluctant addition of a di-anionic intermediate to the naphthalene-1,4-dione or 2-bromonaphthalene-1,4-dione in the solvent medium has been overcome using a neat approach. Practicability of the methodology has been enhanced *via* gram scale solvent-free cascade reaction. The wide substrate scope, biological activity, sustainability, and detailed mechanistic study make our reaction more acceptable in the current scenario.

## Abstract 2

A regioselective sustainable neat approach to naphtho[2,3-*b*]thiophene-4,9-dione has been established *via* detailed mechanistic study and DFT analysis.

## Graphical abstract

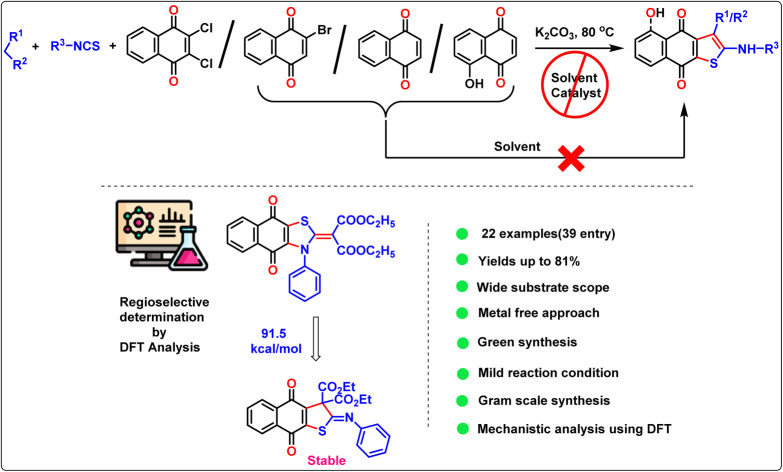

## Introduction

**Fig. 1:**
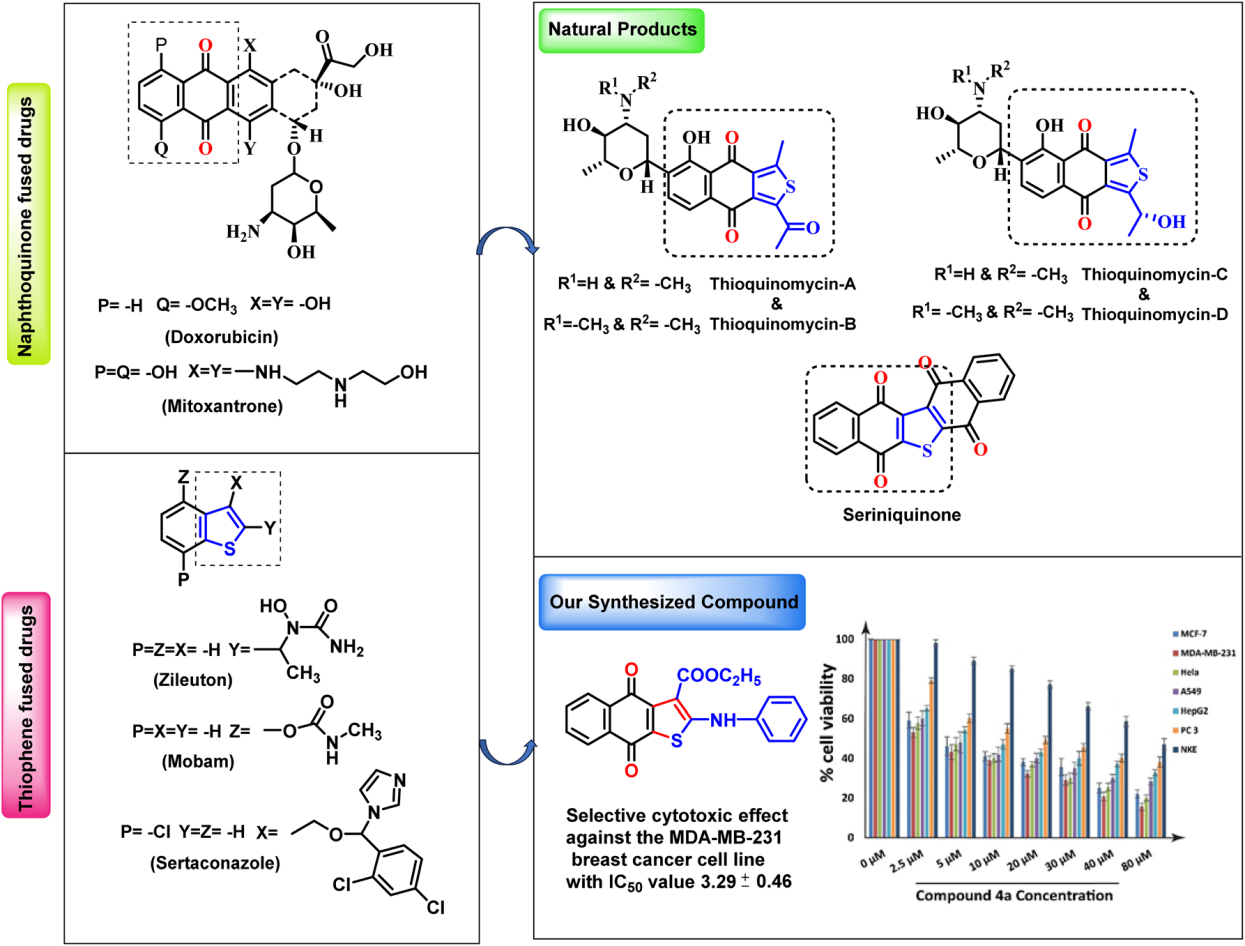
Quinone and thiophene fused drugs and bioactive scaffold of thiophene fused naphthoquinones.

The naphthoquinone and thiophene moieties have shown their importance as a central core structure in various bioactive compounds. The naphthoquinone and thiophene scaffolds exhibit distinct chemical and structural properties that facilitate interactions with particular targets in biological systems. The significant therapeutic effects of naphthoquinone and thiophene-based drugs have prompted researchers to develop and evaluate numerous new molecules. Researchers have successfully developed compounds with enhanced potency and efficacy by incorporating the thiophene moiety into naphthoquinone. Therefore, naphthoquinone-fused thiophene is a significant target for synthetic and medicinal chemists due to its extensive applications across pharmaceutical, biological, and industrial domain. Interestingly, naphthoquinone-fused thiophene exhibits significant biological activity, including anticancer,^1–5^ antiprotozoal,^6–9^ and antipsoriatic effects.^10–12^ Naturally occurring Thioquinomycin A-D, and Serinoquinone exhibit potent cytotoxicity effects on several cancer cell lines (Fig. 1).^13^ FDA [Food and Drug Administration] approved drug molecules in the quinone class include doxorubicin and mitoxantrone.^14^ Commercially available drugs containing thiophene and benzothiophene motifs include zileuton, mobam, and sertaconazole (Fig. 1).^15^ These drugs have a diverse range of medicinal applications. The naphtho[2,3-*b*]thiophene-4,9-dione moiety has been shown to have substantial cytotoxicity against several human solid tumor cell lines that are resistant to doxorubicin and *cis-*platin.^4^

**Scheme 1:**
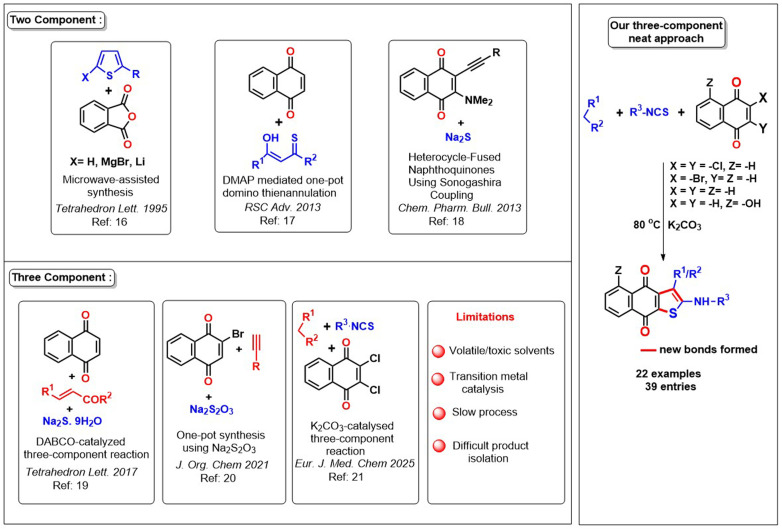
Previous *vs.* present approach to the synthesis of naphtho[2,3-*b*]thiophene-4,9-dione.

Inspired by the significance of naphtho[2,3-*b*]thiophene-4,9-dione, researchers employed a traditional method to synthesize these scaffolds, starting with thiophene or quinone. A. Acosta *et al.*^16^ (Scheme 1) initially developed a microwave-assisted synthesis method for the fabrication of naphtho[2,3-*b*]thiophene-4,9-dione using an annulation approach, with thiophene as the starting material. In subsequent studies, several research groups employed two-component coupling processes to obtain the intended scaffolds. Typically, the researchers used a quinone-embedded framework that was combined with either α-enolicdithioesters/β-oxothioamides^17^ or the Sonogashira coupling product involving 3-bromo-2-(dimethylamino)-5-hydroxynaphthalene-1,4-dione with but-3-yne-2-ol (Scheme 1).^18^ Consequently, researchers are constantly encountering new obstacles in their pursuit of this objective. The three-component reaction is a novel method that enables the synthesis of naphtho[2,3-*b*]thiophene-4,9-dione from either naphthoquinone or bromo naphthoquinone combined with α, β-unsaturated ketones or alkynes in the presence of a sulfur donor (Scheme 1).^19,20^ Recently, we have established a methodology for the synthesis of naphtho[2,3-*b*]thiophene-4,9-dione derivatives using a traditional solvent-added approach.^21^

The previously reported methods required a prolonged reaction period within a sealed tube, while the subsequent method operated in the presence of a transition metal catalyst in the hazardous dioxane solvent. The significant limitations of existing methodologies have motivated us to innovate a more comprehensive, efficient, and practical approach that is also easily implemented.

In continuation of heterocyclic synthesis under sustainable conditions, we want to synthesize anti-cancer bioactive molecules under solvent-free multicomponent reaction. The multi-component reaction is a very pretty method as it produces complex molecules from single reagents without isolation of intermediates.^22^ It not only avoids the toxic solvent and aqueous workup but also reduces the multiple use of column chromatography for each step's separation. Nowadays, it's a challenge to develop an eco-friendly and environmentally benign synthetic strategy. Presently, the multi-component reaction has become an increasingly important method in organic synthesis. They are regarded as efficient synthetic methodologies for the synthesis of diverse functionalized complex molecules in a timely and cost-effective manner.^23^ MCRs are a combination of three or more reactants in one pot procedure, resulting in a new compound with high yields.^24–29^

**Scheme 2:**
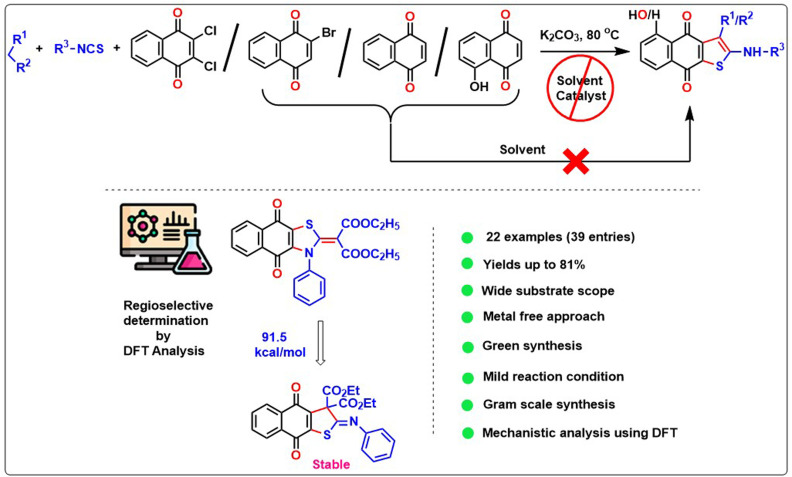
Outlook of our present methodology for naphthothiophene.

Herein, we present a novel and efficient one-pot three-component regioselective synthesis of naphthothiophene (4) using active methylene compounds, alkyl/aryl isothiocyanate, and 1,4-naphthoquinone and its derivatives facilitated by K_2_CO_3_ under neat condition (Scheme 2). The limitation of the previous solvent-added reaction has been overcome using the substrates 2-bromonaphthalene-1,4-dione and naphthalene-1,4-dione for the naphtho[2,3-*b*]thiophene-4,9-diones formation, where we got the desired product under only neat conditions. The protocol has a low *E*-factor compared to the existing methods, and it furnished a broad array of the naphtho[2,3-*b*]thiophene-4,9-diones. Additionally, we have also introduced a brief computational mechanistic observation involving this protocol.

## Results and discussion

In concern to the environmental issue, we initiated our investigation by performing a model reaction utilizing ethyl acetoacetate, phenyl isothiocyanate, and 2,3-dichloro-1,4-naphthoquinone as the primary substrates (Table 1). The reaction was initiated in ethanol without a base by adding 1, 2, and 3A simultaneously at 100 °C for 9 h (entry 1). Regrettably, no required product 4a was found under these specified conditions. Moreover, the reaction was conducted using Et_3_N (3.5 equiv.) as a base in ethanol at a temperature of 60 °C for 9 h, but the desired product 4a was not obtained (entry 2). Similarly, in the presence of another strong base, DABCO (3.0 equiv.) was unable to produce 4a in ethanol or DMSO medium (entries 3 and 4). The lack of success in this reaction motivated us to investigate a more effective combination of base and solvent for our model reaction. Significantly, the replacement of the organic base with K_2_CO_3_ resulted in the production of the desired product 4a with a yield of 21% in DMSO (entry 5). This was achieved by adding 3A to the anion species produced from the reaction with 1 and 2 in the presence of K_2_CO_3_. By substituting DMSO with DMF, a 41% yield of 4a was obtained (entry 7). Furthermore, a 51% yield of compound 4a was obtained by substituting K_2_CO_3_ with Cs_2_CO_3_ (entry 8). The reaction was easily progressed at ambient temperature by sequentially adding 1,2, and 3A (entry 6). Nevertheless, the production of the cyclized product (35%) remains unsatisfactory even after 16 h of stirring. In concern to the environment, we want to establish our reaction under neat conditions, and surprisingly, 4a was obtained effectively with 64% yield at 80 °C (entry 9). By replacing K_2_CO_3_ with Na_2_CO_3_ or CH_3_COONa, the desired target product was obtained with a yield of 51% and 26%, respectively (entries 10 and 11).

**Table 1:** Optimisation of the three-component tandem approach^a^

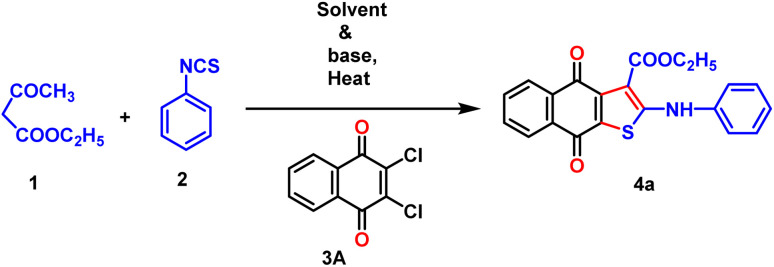					
Entry	Solvent	Base	Temperature (°C)	Time (h)	Yield (%)
1	C_2_H_5_OH	None	100 °C	9	NR
2	C_2_H_5_OH	Et_3_N	60 °C	9	NR
3	C_2_H_5_OH	DABCO	100 °C	9	NR
4	DMSO	DABCO	100 °C	9	NR
5	DMSO	K_2_CO_3_	80 °C	9	21
6	DMF	K_2_CO_3_	RT	16	35
7	DMF	K_2_CO_3_	80 °C	8	41
8	DMF	CS_2_CO_3_	80 °C	6	51
**9**	**Neat**	**K**_**2**_ **CO**_**3**_	**80 °C**	**1**	**64**
10	Neat	Na_2_CO_3_	80 °C	1	51
11	Neat	CH_3_COONa	80 °C	3	26

a Reagent & conditions: substrate 1 (0.1 mmol), substrate 2 (1.1 equiv.), substrate 3 (1 equiv.), base (2.5 equiv.).

Interestingly, the visual color change in a progressive manner helps us to monitor our reaction. A yellow color is observed when substrates 1 and 2 are heated in potassium carbonate, suggesting the formation of a dianion. Subsequently, the inclusion of 3A in the generated dianion through the combination of 1 and 2 results in a color change from yellow to red, therefore indicating the formation of a significant quantity of the product (4a).

**Fig. 2:**
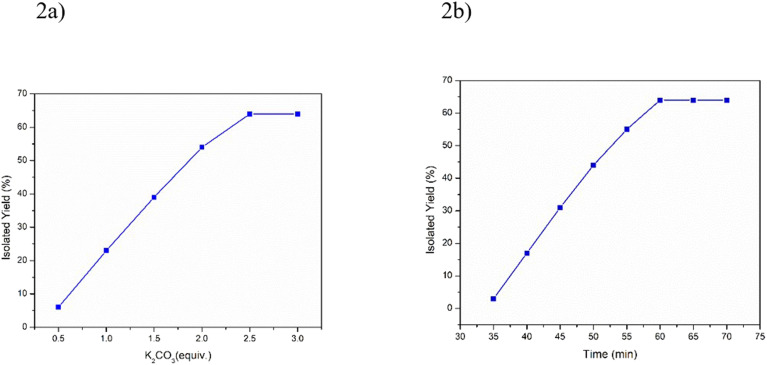
Schematic representation of the potassium carbonate-promoted neat reaction to produce naphtho[2,3-*b*]thiophene-4,9-dione derivatives. (a) Reagent-dependent conversion efficiency of the neat reaction with different mmols of potassium carbonate at constant temperature and time. (b) Time-dependent plot to get the maximum yield at constant temperature and constant equivalent of potassium carbonate.

Remarkably, we have found that the yield of the desired product was increased with a constant enhancement of the K_2_CO_3_ equivalence, which became fixed after the addition of 2.5 equivalent with respect to the quinone substrate 3A, and the yield of the cyclized product was increased up to 1 h (Fig. 2). Hence, the final optimized condition for the three-component coupling reaction under neat condition is substrate 1 (0.1 mmol), substrate 2 (1.1 equiv.), substrate 3 (1 equiv.), K_2_CO_3_ (2.5 equiv.), temperature 80 °C, and time 1 h.

**Fig. 3:**
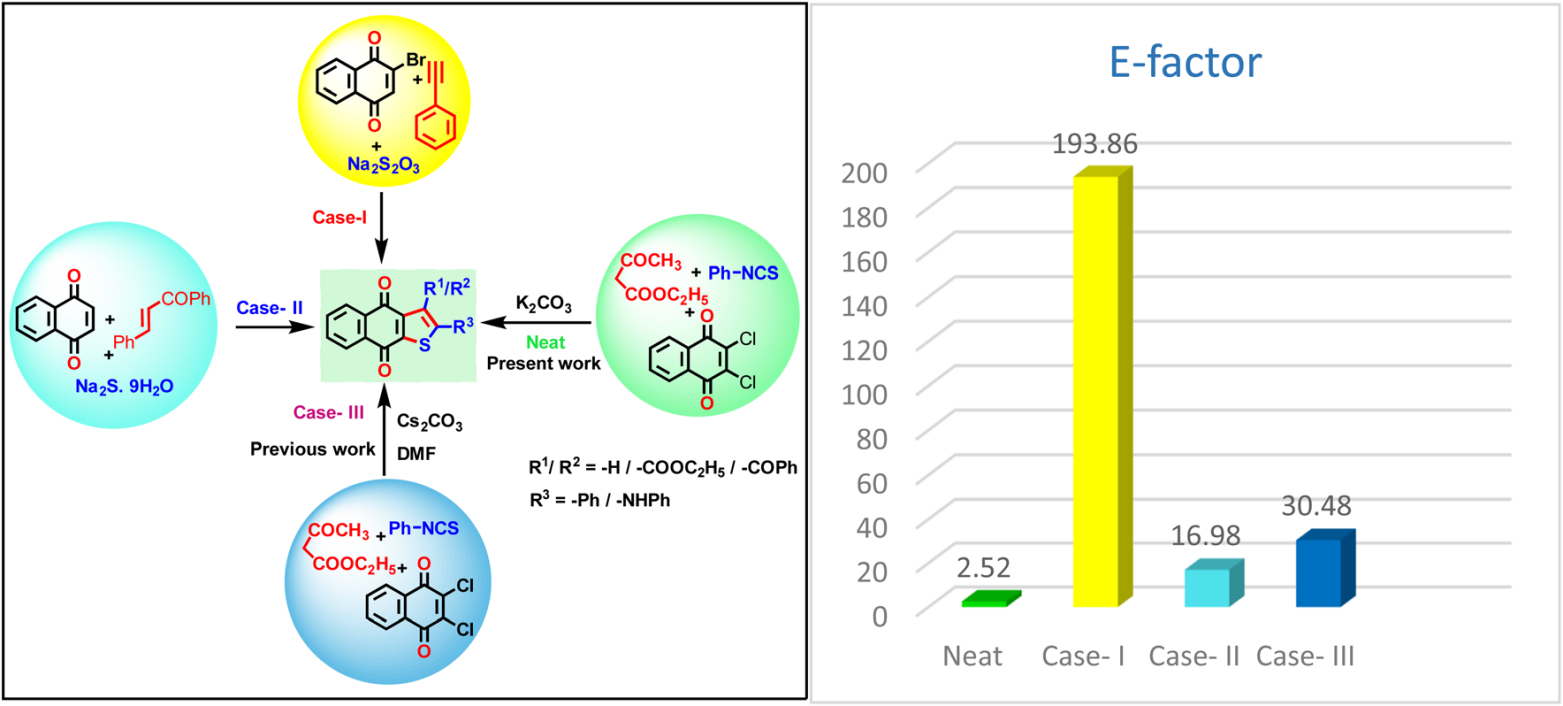
Various synthetic approaches to naphthothiophene and *E*-factor.

Furthermore, we have compared the *E* factor between the existing protocols with the current methodology using the equation *E* factor = [Σ*m* (raw materials) + Σ*m* (reagents) + Σ*m* (solvents) + *m* (water) − *m* (product)]/*m*(product) and obtained a surprising result, which is shown in Fig. 3.^30^ These observations indicated that our protocol furnished lower toxic chemicals compared to the reported one.

Once the optimal condition has been established (Table 1, entry 9), the synthesis of naphtho[2,3-*b*]thiophene-4,9-dione was investigated using different active methylene compounds, alkyl/aryl isothiocyanate, and 2,3-dichloro-1,4-naphthoquinone 3A. Several active methylene compounds easily react with various alkyl/aryl isothiocyanate derivatives and 2,3-dichloro-1,4-naphthoquinone 3A to successfully synthesize the required naphtho[2,3-*b*]thiophene-4,9-dione with moderate to good yields (Tables 2–4). Diethyl malonate, ethyl acetoacetate, diethyl 3-oxopentanedioate, methyl 3-oxobutanoate, methyl 4-methoxy-3-oxobutanoate, *tert*-butyl 3-oxobutanoate, and acetylacetone are active methylene compounds compatible with the reaction (See SI, Table S1). *Tert*-butyl 3-oxobutanoate exhibited superior efficacy with respect to other active methylene compounds. Phenyl isothiocyanate with an electron-donating group (*p*-OMe) (Table 2) provides higher reactivity, and electron-withdrawing groups (*p*-Cl and *p*-NO_2_) (Table 3) provide lower reactivity and yields of moderate magnitude of 4a–4m. The acetylacetone is reluctant to add with the alkyl/aryl isothiocyanate as the intermediate stabilized by the two electron-withdrawing-COMe group, and hence the yields of the ultimate products 4d and 4h are low. The −ve charge on the sulfur atom in the dianion intermediate is stabilized by the chloro (–Cl)/nitro(–NO_2_) group on the isothiocyanate derivatives, resulting in low yields of compound 4l–4m respectively. The beauty of this method is the removal of one ester or acyl group from the active methylene compound 1 after coupling with quinone 3 and alkyl/aryl isothiocyanate 2. During aromatization, diethylmalonate removed one ester group, while ethyl acetoacetate/diethyl 3-oxopentanedioate/methyl 3-oxobutanoate/methyl 4-methoxy-3-oxobutanoate/*tert*-butyl 3-oxobutanoate/acetyl acetone removed the acetyl group in the final product 4.

**Table 2:** Three component neat approach to naphtho[2,3-*b*]thiophene-4,9-dione derivatives from 2,3-dichloro 1,4-naphthoquinone/2-bromo-1,4-naphthoquinone/1,4-naphthoquinone^a^

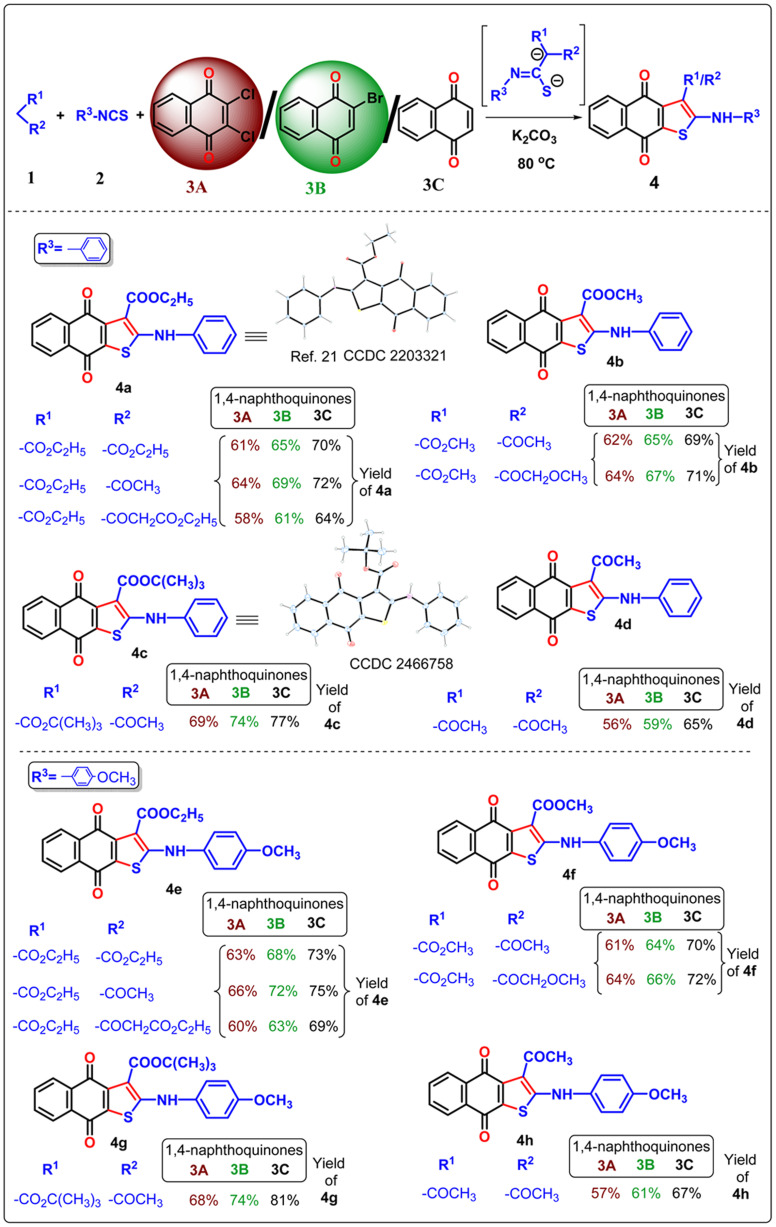

a Reagent and conditions: substrate 1 (0.1 mmol), substrate 2 (1.1 equiv.), substrate 3 (1 equiv.), K_2_CO_3_ (2.5 equiv.), temperature 80 °C, time 1 h.

**Table 3:** Three component neat approach to naphtho[2,3-*b*]thiophene-4,9-dione derivatives from 2,3-dichloro 1,4-naphthoquinone/2-bromo-1,4-naphthoquinone/1,4-naphthoquinone^a^

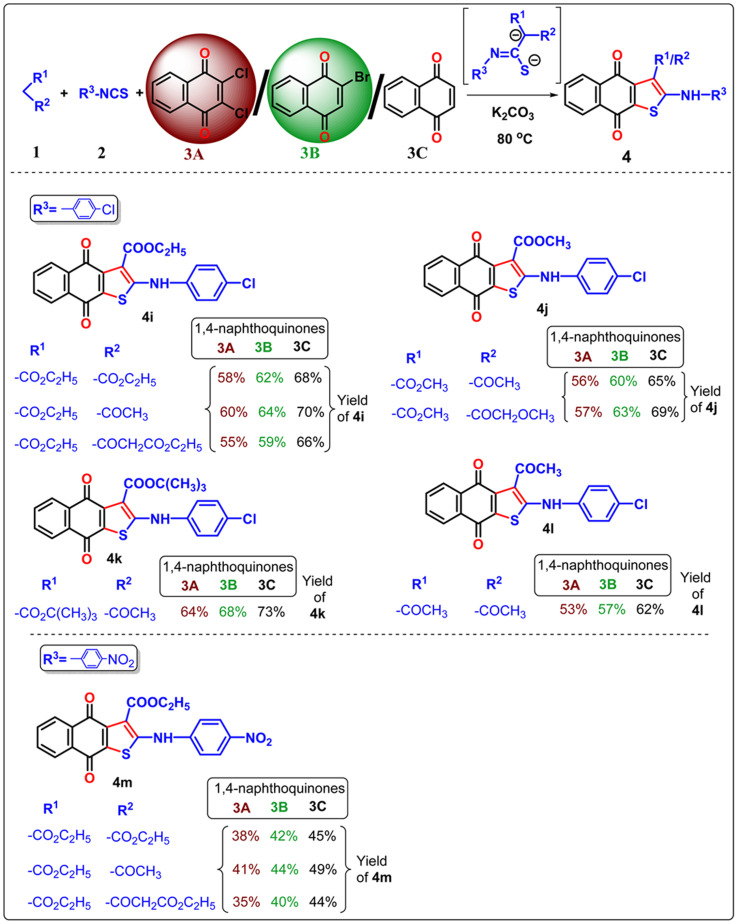

a Reagent and conditions: substrate 1 (0.1 mmol), substrate 2 (1.1 equiv.), substrate 3 (1 equiv.), K_2_CO_3_ (2.5 equiv.), temperature 80 °C, time 1 h.

**Table 4:** Three component neat approach to naphtho[2,3-*b*]thiophene-4,9-dione derivatives from 2,3-dichloro 1,4-naphthoquinone/2-bromo-1,4-naphthoquinone/1,4-naphthoquinone^a^

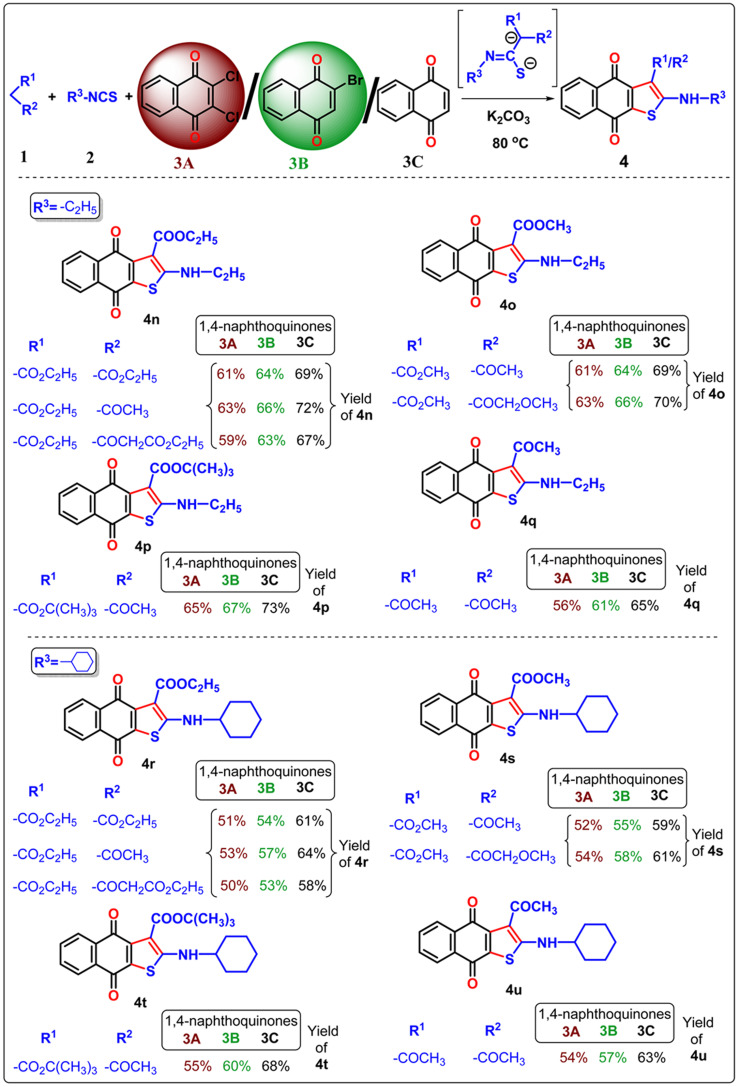

a Reagent and conditions: substrate 1 (0.1 mmol), substrate 2 (1.1 equiv.), substrate 3 (1 equiv.), K_2_CO_3_ (2.5 equiv.), temperature 80 °C, time 1 h.

In the previous communications,^21^ we have only prepared the naphtho[2,3-*b*]thiophene-4,9-dione from the substrate 2,3-dichloronaphthalene-1,4-dione under solvent-added conditions, but this method has been modified *via* environmental benign conditions under neat conditions. Surprisingly, during the extension of the protocol in the presence of 2-bromonaphthalene-1,4-dione or naphthalene-1,4-dione, the reaction did not happen in the addition of solvent with phenyl isothiocyanate (Scheme 2). This problem has been overcome under neat conditions within 1 h. Consequently, the reaction was extended to 2-bromo-1,4-naphthoquinone 3B in the presence of alkyl/aryl isothiocyanate and active methylene compounds at 80 °C under neat conditions. With the same substituted derivatives of alkyl/aryl isothiocyanate, the established technique was well tolerated, and the yield of the desired products 4a–4v (total 22 instances) was significantly higher from 3B than the product obtained from 2,3-dichloro-1,4-naphthoquinone 3A. This observation suggests that the presence of halide substitution in the quinone ring delayed the addition–elimination process. To investigate our methodology and enhance the yield of naphtho[2,3-*b*]thiophene-4,9-dione, we examined the advancement of our reaction with 1,4-naphthoquinone 3C under neat conditions. We observed that the desired product 4 exhibited the best yields compared to 3A and 3B. This observation provides more evidence that the removal of the halide group from the naphthoquinone moieties initiates the addition–elimination reaction, resulting in the formation of naphtho[2,3-*b*]thiophene-4,9-dione derivatives. Hence, we can conclude that the presence of an electron-withdrawing group in the quinone ring lowers the yields of 4. The three components coupling product 4a and 4c were crystallized, and they are shown in Table 2 with the CCDC number.

Our protocol was also successfully applied with aliphatic alkyl isothiocyanate derivatives under neat conditions (Table 4). Ethyl isothiocyanate, cyclohexyl isothiocyanate underwent three component cyclization with significant yields and it was found that the smaller acyclic isothiocyanate furnished dianion intermediate effectively and participate Michael addition with quinone moiety more comfortably than the corresponding cyclic analogue of isothiocyanate. Consequently, the desired 4n–4q furnished with higher yields than 4r–4u.

The diversity of our protocol has also been successfully applied to 5-hydroxy-1,4-naphthoquinone (juglone; obtain from natural source), ethyl acetoacetate, and phenyl isothiocyanate in the presence of K_2_CO_3_ under neat conditions with a satisfactory yield 4v (Table 5).

**Table 5:** Three component neat approach to naphtho[2,3-*b*]thiophene-4,9-dione from 5-hydroxy-1,4-naphthoquinone^a^

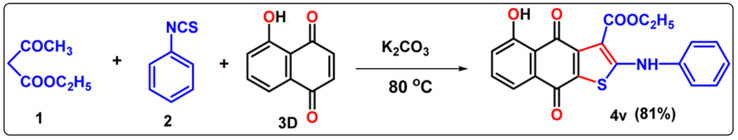

a Reagent and conditions: substrate 1 (0.1 mmol), substrate 2 (1.1 equiv.), substrate 3 (1 equiv.), K_2_CO_3_ (2.5 equiv.), temperature 80 °C, time 1 h.

**Scheme 3:**
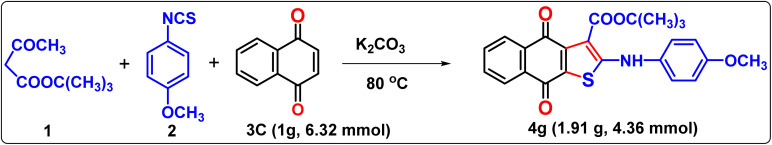
Synthesis of naphthothiophene 4g on a gram scale.

The protocol was also applicable in terms of practical aspects. From an applied standpoint, the reaction was performed on a gram scale and gave a good yield of naphthothiophene derivative 4g under neat conditions (Scheme 3). We obtained 1.91 g (4.36 mmol) product of 4g from 1 g (6.32 mmol) of 3C, yielding a 69% yield of the desired naphthothiophene at 80 °C after 1.5 h of heating. The time required for the gram-scale neat reaction was greater than that of the milligram–scale reaction.

**Scheme 4:**
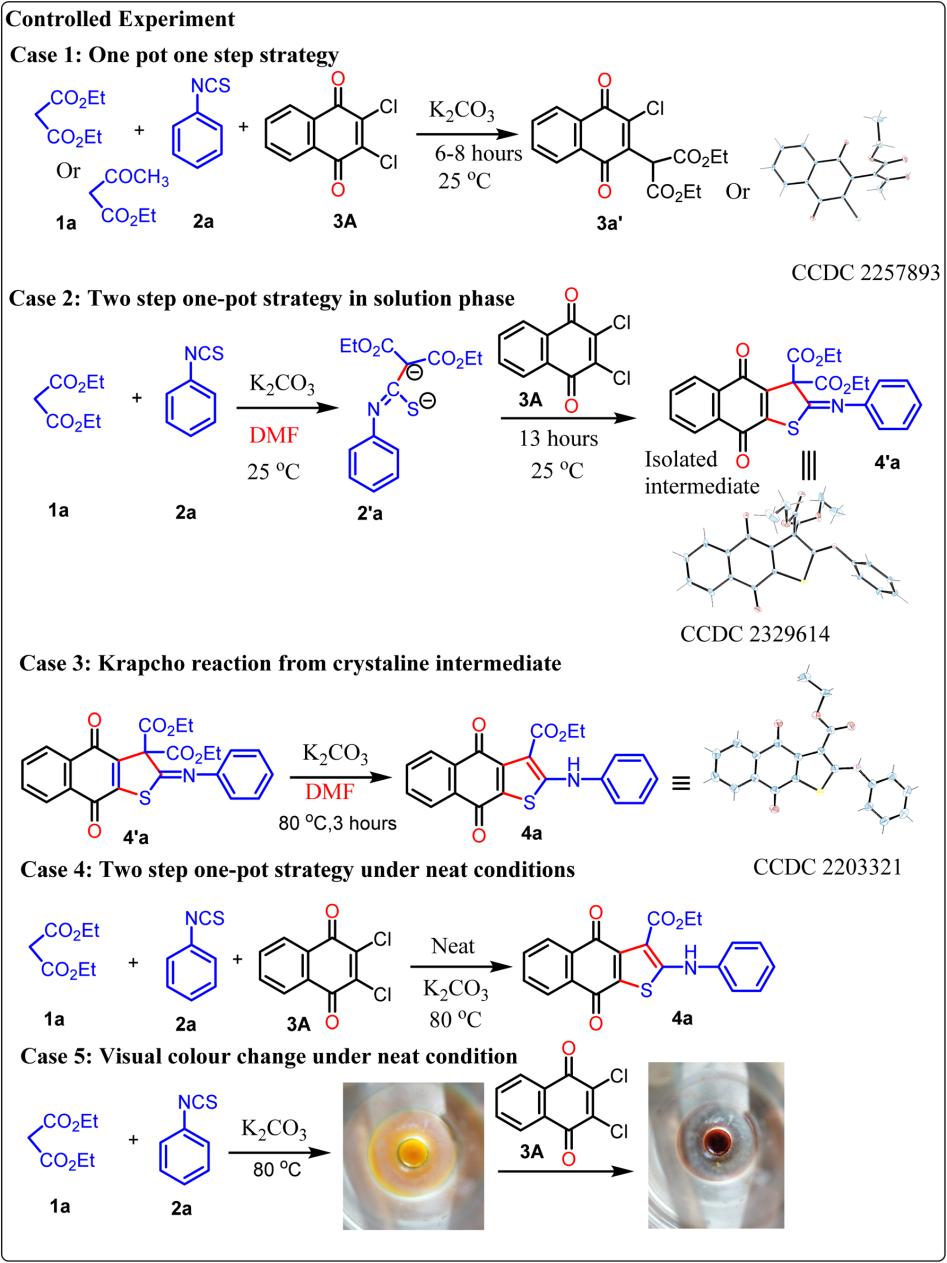
Systematic observations to understand the mechanism.

Once, we have successfully developed a synthetic strategy for the three-component neat approach, our goal is to understand the precise mechanistic pathway of the overall observation by performing a series of controlled experiments (Scheme 4). In the presence of potassium carbonate, we have taken diethylmalonate or ethyl acetoacetate 1, phenyl isothiocyanate 2, and 2,3-dichloro-1,4-napthoquinone 3A. Although we have assessed the feasibility of our method using a one-pot approach by combining all the necessary substrates 1, 2, and 3A simultaneously, we were unable to obtain the cyclized product (case 1). In this reaction, diethylmalonate easily combines with quinone to form a crystalline product 3a′ with a high yield through the replacement of one –Cl atom by an addition–elimination mechanism, which has been confirmed by X-ray crystallographic study. The phenyl isothiocyanate remains inactive in the reaction mixture. This finding offers a vital indication that the formation of dianions is the necessary step to obtain the thiophene-fused quinone derivatives. Hence, we have devised a two-step one-pot strategy where active methylene is allowed to first react with phenyl isothiocyanate in a basic medium to produce a yellow dianion intermediate 2′a within 30 minutes under neat conditions. After that, it reacts with 2,3-dichloro-1,4-napthoquinone to form a crystalline product 4′a within 30 minutes under neat conditions (case 2). Compound 4a is obtained by the Krapcho reaction of the crystalline product 4′a at 80 °C (case 3). The isolation of the intermediate 4′a is the crucial factor for understanding the complete mechanism of the reaction. Curiously, it has been observed that the intermediate cannot stabilize under neat conditions, and three substrates can be easily coupled sequentially to form the required product 4a (case 4). It has been noticed that the visual color change (yellow to red) is an interesting point to monitor the progress of the reaction (case 5).

**Scheme 5:**
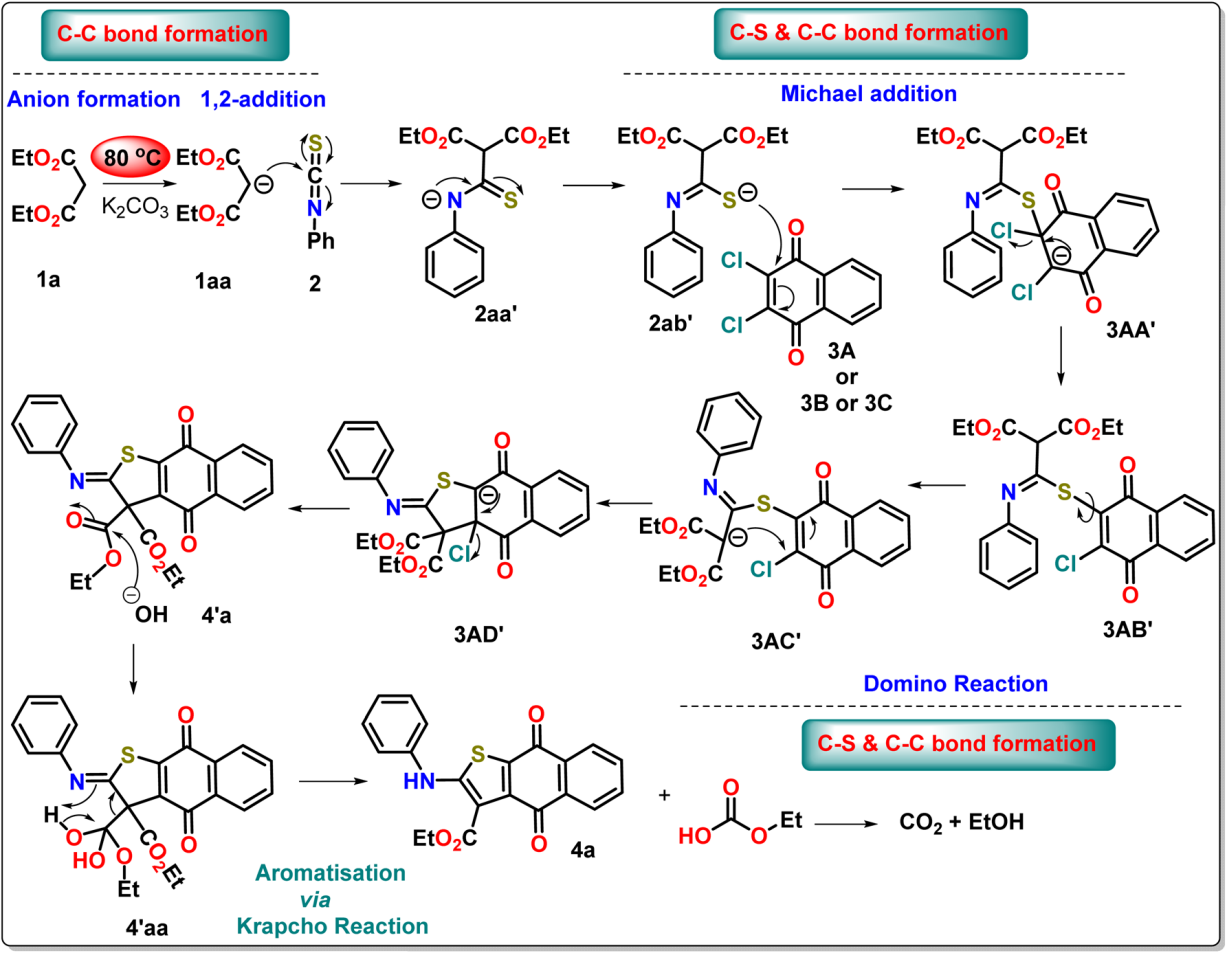
Plausible reaction mechanism for the formation of 4a from the experimental evidence.

Based on the controlled experiment and the isolation of the intermediate, we have proposed a mechanism for the three-component coupling reaction under neat conditions through density functional theory, as this study offers the best opportunity to understand the reaction dynamics (Scheme 5).

### Theoretical study of the mechanism

**Fig. 4:**
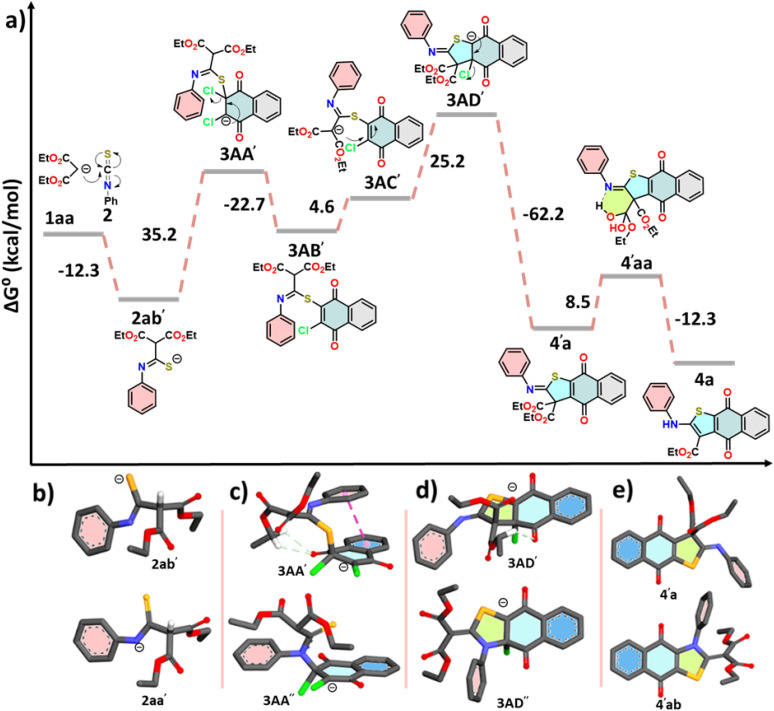
(a) An energy profile diagram for the formation of compound 4. Energy-minimized structures of anionic species (b) 2ab′ and 2aa′, (c) 3AA′ and 3AA″, (d) 3AD′ and 3AD″, and (e) molecules 4′a and 4′ab. Most of the hydrogens have been removed for clarity.

To thoroughly elucidate the reaction leading to the formation of compound 4, we employed density functional theory (DFT). The nucleophilic attack of 1aa on the carbon center of 2 is decidedly spontaneous, yielding a free energy change of −12.2 kcal mol^−1^ (Fig. 4a). This reaction produces 2aa′, which possesses a negative charge on the nitrogen atom but exhibits slight instability (Δ*G*° = −0.4 kcal mol^−1^) when compared to its resonating structure 2ab′, where the sulfur atom holds the negative charge (Fig. 4b). Importantly, both resonating structures (2aa′ and 2ab′) are nearly equally poised for reaction with 3A. However, experimental results compellingly demonstrate that the reaction preferentially occurs through 2ab′, leading to the formation of intermediate 3AA′, a process requiring 35.2 kcal mol^−1^ of free energy. To explain the reason for this sulfur-selective nucleophilic attack, we investigated *N*-site attacks on 3A, resulting in 3AA″ (Fig. 4c). Notably, the conversion from 3A to 3AA″ demands a considerable 74.4 kcal mol^−1^ of free energy. The differences in stability between 3AA′ and 3AA″ are pivotal in this context. The π-stacking interaction between the naphthoquinone ring (blue) and the phenyl ring (wine red), along with the weak hydrogen bond between the naphthoquinone C=O and the methylene hydrogens of the diethyl malonate, significantly stabilizes 3AA′ (Fig. 4c). In stark contrast, 3AA″ lacks these stabilizing intramolecular forces, further supporting the observed selectivity.

A facile chloride elimination (Δ*G*° = −22.7 kcal mol^−1^) from 3AA′ leads directly to a fully conjugated intermediate 3AB′. The deprotonation from the DEM unit of 3AC′ requires an energy input of 5.0 kcal mol^−1^. However, the resulting anion 3AC′ is resonance-stabilised, benefiting from resonance with both the ester groups of the DEM part and the imidothioate part. This resonance (with the negative charge on the carbon centre) enables the formation of an ambident nucleophile, having a negative charge on the nitrogen centre, both of which exhibit comparable stability. The intramolecular nucleophilic cyclisation can proceed through either the carbon centre or the nitrogen centre. Converting 3AC′ to 3AD′ demands 25.2 kcal mol^−1^, while cyclisation through the nitrogen centre to form 3AD″ from the canonical structure of 3AC″ requires 82.2 kcal mol^−1^. Notably, the anionic species 3AD″ is less stable than 3AD′ due to steric interactions between the *N*-phenyl ring and the ethyl groups of the malonate unit (Fig. 4d). This steric strain results in a twisted conformation, disrupting the planarity of the malonate unit and consequently impacting the degree of conjugation in 3AD″. The transformation of compound 3AD′ into 4′a*via* an elimination reaction significantly reduces steric congestion, yielding a conjugation-stabilised product. This reaction releases a substantial free energy of −62.2 kcal mol^−1^, making it spontaneous. In contrast, the elimination reaction involving 3AD″ yields product 4′ab, which shares the same destabilising structural features as 3AD″. Consequently, 4′ab is less stable compared to 4′a (Fig. 4e). In the case of compound 4′a, stabilisation is achieved through conjugation with minimal steric strain. The free energy difference between 4′a and 4′ab is 91.5 kcal mol^−1^. The final product, identified as compound 4, is generated through the removal of one ester group from the DEM unit *via* the Krapcho reaction. This mechanism involves a nucleophilic attack by a hydroxyl ion, which is produced from the reaction of K_2_CO_3_ with water, targeting one of the ester centres in compound 4′a. This interaction results in the formation of an intermediate, labelled 4′aa, which is stabilised by hydrogen bonding. The free energy required for this step is estimated at 8.5 kcal mol^−1^. Following this, the decomposition of compound 4′aa leads to the formation of 4a and ethyl hydrogen carbonate, with the latter releasing CO_2_ as a gas. This gas release not only drives the reaction forward but also increases entropy, consistent with Le Châtelier's principle. This step is spontaneous by release −12.3 kcal mol^−1^.

## Conclusion

In conclusion, we have established a three-component sustainable cascade protocol for synthesizing 2,3-disubstituted anti-cancer naphtho[2,3-*b*]thiophene-4,9-diones in favourable yields utilizing active methylene compounds, alkyl/aryl isothiocyanates, and either 2,3-dichloro-1,4-naphthoquinone, 2-bromo-1,4-naphthoquinone, 1,4-naphthoquinone or 5-hydroxy-1,4-naphthoquinone. The gram-scale synthesis of fused naphtho[2,3-*b*]thiophene-4,9-diones is another practical application of this method. The regioselective addition of dianion intermediate to quinone system has been recognized using DFT analysis.

## Experimental section

Reagents, starting materials, and solvents were obtained from commercial suppliers and used without further purification. Analytical TLC was carried out on Merck silica gel GF 254 plates. The crude product was purified by column chromatography using silica gel (100–200 mesh) with a 1 : 20 mixture of ethyl acetate and petroleum ether as the eluent to isolate the desired products. ^1^H NMR and ^13^C NMR spectra were recorded on a Bruker spectrometer at 300, 400, 500, 600 MHz and 75, 101, 126, 151 MHz, respectively. Chemical shifts are reported in *δ* values (ppm) relative to tetramethylsilane (Me_4_Si) as an internal standard, and coupling constants (*J*) are given in hertz (Hz). TOF MS ES+ spectra were recorded on a Bruker mass spectrometer.

### General procedure for the synthesis of naphtho[2,3-*b*]thiophene-4,9-diones (4a–4v)

Active methylene compound (0.1 mmol), alkyl/aryl isothiocyanate (1.1 equiv., 0.11 mmol), and 2.5 equiv. K_2_CO_3_ was added to a hard glass test tube. The mixture was heated at 80 °C for 30 minutes in an oil bath. Once the dianion formation was complete, then 1,4-naphthoquinone (1 equiv. 0.1 mmol) was added to the reaction mixture, and the heating was continued at 80 °C for an additional 30 minutes. Upon completion, checked by TLC, the reaction mixture was acidified with dilute HCl, extracted with ethyl acetate (3 × 10.0 mL), followed by washing with brine water (3 × 10.0 mL). The organic layer was dried over sodium sulfate (Na_2_SO_4_). After evaporating the solvent, the crude product was purified by column chromatography on silica gel (100–200 mesh) using an ethyl acetate-petroleum ether (1 : 20) eluent to get the pure desired products 4.

#### Diethyl (*Z*)-4,9-dioxo-2-(phenylimino)-4,9-dihydronaphtho[2,3-*b*]thiophene-3,3(2H) dicarboxylate (4′a)

Yellow crystalline solid (71%, 31.8 mg), m.p. 108–110 °C, ^1^H NMR (300 MHz, CDCl_3_) *δ* 8.17–8.09 (m, 2H), 7.83–7.71 (m, 2H), 7.43–7.28 (m, 2H), 7.25–7.21 (m, 1H), 7.01–6.97 (m, 2H), 4.42–4.31 (m, 4H), 1.31 (t, *J* = 7.2 Hz, 6H). ^13^C NMR (75 MHz, CDCl_3_) *δ* 179.5, 177.1, 163.8, 159.0, 153.6, 149.9, 138.1, 134.9 (2C), 133.8 (2C), 132.6, 132.0, 129.6 (2C), 127.4, 126.9, 126.6, 119.8 (2C), 63.5 (2C), 14.1 (2C). LCMS (ESI) *m*/*z*: [M + H]^+^ calcd for C_24_H_19_NO_6_S: 450.1013 found 450.1046.

#### Ethyl 4,9-dioxo-2-(phenylamino)-4,9-dihydronaphtho[2,3-*b*]thiophene-3-carboxylate (4a)

Red crystalline solid, 72% yield, 27.1 mg; m.p. 142–144 °C, ^1^H NMR (400 MHz, CDCl_3_) *δ* 10.05 (s, 1H), 8.15–8.11 (m, 2H), 7.72–7.70 (m, 2H), 7.49–7.45 (m, 2H), 7.41–7.39 (m, 2H), 7.27–7.23 (m, 1H), 4.46 (q, *J* = 7.1 Hz, 2H), 1.47 (t, *J* = 7.1 Hz, 3H). ^13^C NMR (101 MHz, CDCl_3_) *δ* 178.6, 177.5, 165.9, 163.9, 141.6, 139.3, 134.6, 133.4, 133.1, 132.6, 130.0 (2C), 129.6, 127.3, 125.8, 125.6, 120.7 (2C), 107.3, 61.4, 14.1. HRMS (ESI) *m*/*z*: [M + Na]^+^ calcd for C_21_H_15_NO_4_S: 400.0722 found 400.0721.

#### Methyl 4,9-dioxo-2-(phenylamino)-4,9-dihydronaphtho[2,3-*b*]thiophene-3-carboxylate (4b)

Red crystalline solid, 71% yield, 25.7 mg; m.p. 125–127 °C, ^1^H NMR (600 MHz, CDCl_3_) *δ* 10.05 (s, 1H), 8.15–8.12 (m, 2H), 7.73–7.70 (m, 2H), 7.49–7.46 (m, 2H), 7.41–7.39 (m, 2H), 7.26–7.24 (m, 1H), 4.00–3.99 (m, 3H). ^13^C NMR (151 MHz, CDCl_3_) *δ* 178.5, 177.5, 166.4, 164.2, 141.5, 139.3, 134.5, 133.4, 133.2, 132.6, 130.0 (2C), 127.4, 125.8, 125.8, 120.9 (2C), 106.7, 52.2. HRMS (ESI) *m*/*z*: [M – H]^+^ calcd for C_20_H_12_NO_4_S: 362.0485 found 362.0482.

#### Tert-butyl 4,9-dioxo-2-(phenylamino)-4,9-dihydronaphtho[2,3-*b*]thiophene-3-carboxylate (4c)

Red crystalline solid, 77% yield, 31.2 mg; m.p. 152–154 °C, ^1^H NMR (400 MHz, CDCl_3_) *δ* 10.01 (s, 1H), 8.14–8.11 (m, 2H), 7.72–7.69 (m, 2H), 7.49–7.44 (m, 2H), 7.41–7.39 (m, 2H), 7.26–7.22 (m, 1H), 1.67 (s, 9H). ^13^C NMR (151 MHz, CDCl_3_) *δ* 178.7, 177.5, 165.2, 163.2, 142.0, 139.4, 134.8, 133.3, 133.0, 132.7, 129.9 (2C), 129.1, 128.4, 127.1, 125.8, 125.3, 120.4 (2C), 109.2, 82.7, 28.2. HRMS (ESI) *m*/*z*: [M − H]^+^ calcd for C_23_H_18_NO_4_S: 404.0955 found 404.0947.

#### 3-Acetyl-2-(phenylamino)naphtho[2,3-*b*]thiophene-4,9-dione (4d)

Red crystalline solid, 65% yield, 22.5 mg; m.p. 158–160 °C, ^1^H NMR (400 MHz, CDCl_3_) *δ* 11.45 (s, 1H), 8.17–8.13 (m, 2H), 7.76–7.74 (m, 2H), 7.50–7.47 (m, 2H), 7.43–7.41 (m, 2H), 7.31–7.30 (m, 1H), 2.71 (s, 3H). ^13^C NMR (101 MHz, CDCl_3_) *δ* 198.4, 180.5, 177.7, 164.9, 141.2, 139.2, 134.2, 133.6, 133.5, 132.6, 130.0 (2C), 127.4, 126.1, 125.9, 121.4 (2C), 115.8, 31.6. LCMS (ESI) calcd for C_20_H_13_NO_3_S [M + H]^+^: 348.0616 found 348.0681.

#### Ethyl 2-((4-methoxyphenyl)amino)-4,9-dioxo-4,9-dihydronaphtho[2,3-*b*]thiophene-3-carboxylate (4e)

Red solid, 75% yield, 30.5 mg; m.p. 128–130 °C, ^1^H NMR (400 MHz, CDCl_3_) *δ* 9.75 (s, 1H), 8.14–8.09 (m, 2H), 7.71–7.68 (m, 2H), 7.33–7.31 (m, 2H), 7.01–6.97 (m, 2H), 4.45 (q, *J* = 7.1 Hz, 2H), 3.87 (s, 3H), 1.46 (t, *J* = 7.1 Hz, 3H). ^13^C NMR (101 MHz, CDCl_3_) *δ* 178.6, 177.4, 166.5, 165.9, 158.0, 142.0, 134.6, 133.2, 132.9, 132.6, 132.5, 129.2, 127.2, 125.7, 123.9 (2C), 115.2 (2C), 106.1, 61.2, 55.6, 14.1. Mass calcd for C_22_H_17_NO_5_S [M]^+^: 407.0827 found: 407.2591.

#### Methyl 2-((4-methoxyphenyl)amino)-4,9-dioxo-4,9-dihydronaptho[2,3-*b*]thiophene-3-carboxylate (4f)

Red solid, 72% yield, 28.3 mg; m.p. 112–114 °C, ^1^H NMR (400 MHz, CDCl_3_) *δ* 9.76 (s, 1H), 8.14–8.10 (m, 2H), 7.72–7.69 (m, 2H), 7.34–7.32 (m, 2H), 7.01–6.98 (m, 2H), 3.99 (s, 3H), 3.87 (s, 3H). ^13^C NMR (151 MHz, CDCl_3_) *δ* 178.9, 177.5, 166.8, 166.4, 158.1, 141.8, 134.6, 133.3, 133.1, 132.6, 132.4, 129.4, 127.3, 125.7, 124.1 (2C), 115.2 (2C), 105.5, 55.6, 52.1. HRMS (ESI) *m*/*z*: [M − H]^+^ calcd for C_21_H_14_NO_5_S: 392.0591 found 392.0595.

#### Tert-butyl 2-((4-methoxyphenyl)amino)-4,9-dioxo-4,9-dihydronaphtho[2,3-*b*]thiophene-3-carboxylate (4g))

Red solid, 81% yield, 35.2 mg; m.p. 136–138 °C, ^1^H NMR (400 MHz, CDCl_3_) *δ* 9.72 (s, 1H), 8.11–8.08 (m, 2H), 7.69–7.67 (m, 2H), 7.33–7.30 (m, 2H), 6.99–6.96 (m, 2H), 3.86–3.85 (m, 3H), 1.66 (s, 9H). ^13^C NMR (151 MHz, CDCl_3_) *δ* 178.8, 177.4, 165.8, 165.3, 157.8, 142.3, 134.8, 133.1, 132.9, 132.7, 132.6, 128.6, 127.0, 125.7, 123.6 (2C), 115.0 (2C), 107.9, 82.4, 55.6, 28.2 (3C). HRMS (ESI) *m*/*z*: [M − H]^+^ calcd for C_24_H_20_NO_5_S: 434.1060 found 434.1061.

#### 3-Acetyl-2-((4-methoxyphenyl)amino)naphtho[2,3-*b*]thiophene-4,9-dione (4h)

Red solid, 67% yield, 25.2 mg; m.p. 170–172 °C, ^1^H NMR (300 MHz, CDCl_3_) *δ* 11.15 (s, 1H), 8.12–8.07 (m, 2H), 7.71–7.68 (m, 2H), 7.31–7.28 (m, 2H), 6.97–6.95 (m, 2H), 3.84 (s, 3H), 2.67 (s, 3H). ^13^C NMR (75 MHz, CDCl_3_) *δ* 198.2, 180.5, 177.7, 167.4, 141.6, 134.3, 133.5 (3C), 132.7, 132.4, 127.4, 125.9, 124.4 (3C), 115.2 (2C), 115.0, 55.7, 31.6. Mass calcd for C_21_H_15_NO_4_S [M]^+^: 377.0722 found 377.8676.

#### Ethyl 2-((4-chlorophenyl)amino)-4,9-dioxo-4,9-dihydronaphtho[2,3-*b*]thiophene-3-carboxylate (4i)

Red solid, 70% yield, 28.7 mg; m.p. 168–170 °C, ^1^H NMR (300 MHz, CDCl_3_) *δ* 10.04 (s, 1H), 8.16–8.12 (m, 2H), 7.74–7.70 (m, 2H), 7.45–7.42 (m, 2H), 7.35–7.32 (m, 2H), 4.46 (q, *J* = 7.1 Hz, 2H), 1.46 (t, *J* = 7.1 Hz, 3H). ^13^C NMR (75 MHz, CDCl_3_) *δ* 178.5, 177.5, 165.9, 163.3, 141.5, 137.9, 134.5, 133.5, 133.1, 132.5, 130.7, 130.0 (2C), 129.0, 127.4, 125.8, 121.9 (2C), 107.7, 61.5, 14.1. Mass calcd for C_21_H_14_ClNO_4_S [M]^+^: 412.0410 found 412.4849.

#### Methyl 2-((4-chlorophenyl)amino)-4,9-dioxo-4,9-dihydronaphtho[2,3-*b*]thiophene-3-carboxylate (4j)

Red solid, 69% yield, 27.3 mg; m.p. 152–154 °C, ^1^H NMR (400 MHz, CDCl_3_) *δ* 10.03 (s, 1H), 8.14–8.11 (m, 2H), 7.73–7.71 (m, 2H), 7.45–7.41 (m, 2H), 7.35–7.32 (m, 2H), 3.99 (s, 3H). ^13^C NMR (151 MHz, CDCl_3_) *δ* 178.6, 177.5, 166.4, 163.7, 141.4, 137.9, 134.5, 133.5, 133.2, 132.5, 130.9, 130.0 (2C), 129.2, 127.4, 125.9, 122.1 (2C), 107.1, 52.3. HRMS (ESI) *m*/*z*: [M – H]^+^ calcd for C_20_H_11_ClNO_4_S: 396.0096 found 396.0091.

#### Tert-butyl 2-((4-chlorophenyl)amino)-4,9-dioxo-4,9-dihydronaphtho[2,3-*b*]thiophene-3-carboxylate (4k)

Red solid, 73% yield, 32.0 mg; m.p. 174–176 °C, ^1^H NMR (600 MHz, CDCl_3_) *δ* 10.02 (s, 1H), 8.14–8.11 (m, 2H), 7.72–7.70 (m, 2H), 7.43–7.41 (m, 2H), 7.34–7.32 (m, 2H), 1.66 (s, 9H). ^13^C NMR (151 MHz, CDCl_3_) *δ* 178.5, 177.5, 165.2, 162.7, 141.9, 138.0, 134.7, 133.4, 133.0, 132.6, 130.0 (2C), 128.7, 127.1, 125.8, 123.9, 121.6 (2C), 109.5, 82.9, 28.1 (3C). HRMS (ESI) *m*/*z*: [M + Na]^+^ calcd for C_23_H_18_ClNO_4_S: 462.0542 found 462.0531.

#### 3-Acetyl-2-((4-chlorophenyl)amino)naphtho[2,3-*b*]thiophene-4,9-dione (4l)

Red solid, 62% yield, 23.6 mg; m.p. 148–150 °C, ^1^H NMR (500 MHz, CDCl_3_) *δ* 11.42 (s, 1H), 8.17–8.14 (m, 2H), 7.77–7.75 (m, 2H), 7.45–7.44 (m, 2H), 7.37–7.35 (m, 2H), 2.71 (s, 3H). ^13^C NMR (126 MHz, CDCl_3_) *δ* 198.5, 180.1, 177.6, 164.4, 141.1, 137.7, 134.2, 133.6, 132.5, 131.3, 130.4, 130.1 (2C), 129.0, 127.5, 125.9, 122.6 (2C), 116.1, 31.6. Mass calcd for C_20_H_12_ClNO_3_S [M + Na + H]^+^:405.0201 found: 405.2555.

#### Ethyl 2-((4-nitrophenyl)amino)-4,9-dioxo-4,9-dihydronaphtho[2,3-*b*]thiophene-3-carboxylate (4m)

Orange solid, 49% yield, 20.6 mg; m.p. 180–182 °C, ^1^H NMR (400 MHz, CDCl_3_) *δ* 10.53 (s, 1H), 8.37–8.33 (m, 2H), 8.19–8.17 (m, 2H), 7.78–7.75 (m, 2H), 7.50–7.48 (m, 2H), 4.50 (q, *J* = 7.2 Hz, 2H), 1.48 (t, *J* = 7.1 Hz, 3H). ^13^C NMR (101 MHz, CDCl3) *δ* 178.1, 177.2, 169.9, 165.7, 159.1, 144.6, 143.3, 134.5, 133.8, 133.3, 132.4, 131.1, 127.5, 126.1, 126.0 (2C), 118.1 (2C), 110.8, 61.9, 14.0. Mass calcd for C_21_H_14_N_2_O_6_S [M + Na]^+^:445.0470 found 445.1604.

#### Ethyl 2-(ethylamino)-4,9-dioxo-4,9-dihydronaphtho[2,3-*b*]thiophene-3-carboxylate (4n)

Deep red solid, 72% yield, 23.6 mg; m.p. 125–127 °C, ^1^H NMR (500 MHz, CDCl_3_) *δ* 8.12–8.11 (m, 2H), 8.09 (s, 1H), 7.70–7.68 (m, 2H), 4.40 (q, *J* = 7.1 Hz, 2H), 3.44–3.39 (m, 2H), 1.45–1.41 (m, 6H). ^13^C NMR (126 MHz, CDCl3) *δ* 178.9, 177.2, 169.1, 165.8, 142.8, 134.7, 133.0, 132.9, 132.7, 129.0, 127.2, 125.6, 103.8, 60.9, 42.6, 29.7, 14.2. Mass calcd for C_17_H_15_NO_4_S [M + H]^+^: 330.0800 found 330.1377.

#### Methyl 2-(ethylamino)-4,9-dioxo-4,9-dihydronaphtho[2,3-*b*]thiophene-3-carboxylate (4o)

Deep red solid, 70% yield, 22.0 mg; m.p. 120–122 °C, ^1^H NMR (400 MHz, CDCl_3_) *δ* 8.11–8.09 (m, 3H), 7.69–7.67 (m, 2H), 3.92 (s, 3H), 3.44–3.37 (m, 2H), 1.44–1.40 (m, 3H). ^13^C NMR (151 MHz, CDCl_3_) *δ* 179.0, 177.2, 169.3, 166.3, 142.6, 134.6, 133.2, 133.0, 132.6, 129.1, 127.3, 125.7, 103.3, 51.8, 42.7, 14.0. HRMS (ESI) *m*/*z*: [M − H]^+^ calcd for C_16_H_12_NO_4_S: 314.0485 found 314.0487.

#### Tert-butyl 2-(ethylamino)-4,9-dioxo-4,9-dihydronaphtho[2,3-*b*]thiophene-3-carboxylate (4p)

Deep red solid, 73% yield, 26.0 mg; m.p. 134–136 °C, ^1^H NMR (600 MHz, CDCl_3_) *δ* 8.11–8.06 (m, 2H), 7.99–7.98 (m, 1H), 7.68–7.65 (m, 2H), 3.41–3.36 (m, 2H), 1.62 (s, 9H), 1.43–1.40 (m, 3H). ^13^C NMR (151 MHz, CDCl_3_) *δ* 178.9, 177.1, 168.8, 165.1, 143.3, 134.9, 133.0, 132.8, 132.8, 128.3, 127.0, 125.6, 105.6, 81.9, 42.6, 28.3 (3C), 14.06. HRMS (ESI) *m*/*z*: [M − H]^+^ calcd for C_19_H_18_NO_4_S: 356.0955 found 356.0949.

#### 3-Acetyl-2-(ethylamino)naphtho[2,3-*b*]thiophene-4,9-dione (4q)

Deep red solid, 65% yield, 19.4 mg; m.p. 110–112 °C, ^1^H NMR (500 MHz, CDCl_3_) *δ* 9.56 (s, 1H), 8.14–8.11 (m, 2H), 7.73–7.71 (m, 2H), 3.45–3.39 (m, 2H), 2.63 (s, 3H), 1.43 (t, *J* = 7.3 Hz, 3H). ^13^C NMR (126 MHz, CDCl_3_) *δ* 197.5, 180.5, 177.3, 169.7, 142.2, 134.3, 133.4, 133.3, 132.7, 129.4, 127.3, 125.7, 113.2, 42.8, 31.4, 13.9. Mass calcd for C_16_H_13_NO_3_S [M + H]^+^: 300.0694 found 300.3356.

#### Ethyl 2-(cyclohexylamino)-4,9-dioxo-4,9-dihydronaphtho[2,3-*b*]thiophene-3-carboxylate (4r)

Red solid, 64% yield, 24.5 mg; m.p. 126 °C–128 °C, ^1^H NMR (500 MHz, CDCl_3_) *δ* 8.21–8.19 (m, 1H), 8.11–8.09 (m, 2H), 7.69–7.67 (m, 2H), 4.39 (q, *J* = 7.1 Hz, 2H), 3.36–3.33 (m, 1H), 2.14–2.11 (m, 2H), 1.84–1.81 (m, 2H), 1.69–1.67 (m, 2H), 1.48–1.46 (m, 1H), 1.44–1.41 (m, 6H). ^13^C NMR (126 MHz, CDCl_3_) *δ* 178.8, 177.2, 168.0, 165.9, 142.9, 134.7, 133.0, 132.9, 132.7, 128.8, 127.2, 125.6, 103.7, 60.8, 57.3, 32.1 (2C), 25.3, 24.4 (2C), 14.2. Mass calcd for C_21_H_21_NO_4_S [M + H]^+^: 384.1271 found 384.4041.

#### Methyl 2-(cyclohexylamino)-4,9-dioxo-4,9-dihydronaphtho[2,3-*b*]thiophene-3-carboxylate (4s)

Red solid, 61% yield, 22.5 mg; m.p. 118–120 °C, ^1^H NMR (600 MHz, CDCl_3_) *δ* 8.25–8.24 (m, 1H), 8.11–8.09 (m, 2H), 7.69–7.67 (m, 2H), 3.92 (s, 3H), 3.36–3.33 (m, 1H), 2.15–2.11 (m, 2H), 1.85–1.80 (m, 2H), 1.73–1.67 (m, 3H), 1.46–1.42 (m, 3H). ^13^C NMR (151 MHz, CDCl_3_) *δ* 179.0, 177.2, 168.1, 166.4, 142.7, 134.6, 133.1, 133.0, 132.6, 129.0, 127.3, 125.6, 103.1, 57.3, 51.8, 32.1 (2C), 25.3, 24.4 (2C). HRMS (ESI) *m*/*z*: [M − H]^+^ calcd for C_20_H_18_NO_4_S: 368.0955 found 368.0950.

#### Tert-butyl 2-(cyclohexylamino)-4,9-dioxo-4,9-dihydronaphtho[2,3-*b*]thiophene-3-carboxylate (4t)

Red solid, 68% yield, 27.9 mg; m.p. 138–140 °C, ^1^H NMR (600 MHz, CDCl_3_) *δ* 8.10–8.08 (m, 1H), 8.07–8.05 (m, 2H), 7.67–7.65 (m, 2H), 3.34–3.28 (m, 1H), 2.14–2.12 (m, 2H), 1.83–1.81 (m, 2H), 1.62–1.61 (m, 9H), 1.44–1.41 (m, 3H), 1.30–1.27 (m, 3H). ^13^C NMR (151 MHz, CDCl_3_) *δ* 178.9, 177.1, 167.6, 165.1, 143.4, 134.9, 132.9 (2C), 128.1, 127.0, 125.6, 105.5, 81.8, 57.3, 32.2 (2C), 29.7, 28.2(3C), 25.3, 24.5 (2C). HRMS (ESI) *m*/*z*: [M − H]^+^ calcd for C_23_H_24_NO_4_S: 410.1424 found 410.1419.

#### 3-Acetyl-2-(cyclohexylamino)naphtho[2,3-*b*]thiophene-4,9-dione (4u)

Deep red solid, 63% yield, 22.2 mg; m.p. 117–119 °C, ^1^H NMR (500 MHz, CDCl_3_) *δ* 9.74–9.72 (m, 1H), 8.14–8.11 (m, 2H), 7.73–7.71 (m, 2H), 3.39–3.33 (m, 1H), 2.63 (s, 3H), 2.14–2.10 (m, 2H), 1.86–1.81 (m, 2H), 1.71 (s, 1H), 1.49–1.44 (m, 2H), 1.28–1.27 (m, 3H). ^13^C NMR (126 MHz, CDCl_3_) *δ* 197.4, 180.5, 177.4, 168.5, 142.3, 134.3, 133.3, 133.2, 132.7, 127.3, 125.7, 113.1, 59.5, 38.2, 32.0, 31.2, 29.7 (2C), 29.4, 22.7. HRMS (ESI) *m*/*z*: [M + H]^+^ calcd for C_20_H_20_NO_3_S: 354.1166 found 354.1169.

#### Ethyl 5-hydroxy-4,9-dioxo-2-(phenylamino)-4,9-dihydronaphtho[2,3-*b*]thiophene-3-carboxylate(4v)

Red crystalline solid, 81% yield, 31.8 mg, m.p. 148–150 °C, ^1^H NMR (600 MHz, CDCl_3_) *δ* 12.06 (s, 1H), 10.08 (s, 1H), 7.64–7.63 (m, 1H), 7.57–7.54 (m, 1H), 7.48–7.46 (m, 1H), 7.39–7.38 (m, 2H), 7.27–7.23 (m, 2H), 7.20–7.18 (m, 1H), 4.47–4.43 (m, 2H), 1.47–1.44 (m, 3H). ^13^C NMR (101 MHz, CDCl_3_) *δ* 182.36, 177.94, 165.73, 164.38, 161.06, 142.56, 139.18, 135.59, 134.64, 129.98 (2C), 125.82, 123.63, 120.79 (2C), 120.73, 120.00, 115.11, 107.80, 61.49, 14.08. HRMS (ESI) *m*/*z*: [M + H]^+^ calcd for C_21_H_16_NO_5_S: 394.0751 found 394.0750.

## Conflicts of interest

There are no conflicts to declare.

## Supplementary Material

RA-016-D5RA08362A-s001

RA-016-D5RA08362A-s002

## Data Availability

CCDC 2203321, 2257893, 2329614 and 2466758 contain the supplementary crystallographic data for this paper.^31*a*–*d*^ The data supporting this article have been included as part of the supplementary information (SI). Supplementary information: experimental details, characterization and DFT data. See DOI: https://doi.org/10.1039/d5ra08362a.
