# Supplementary material for: A regioselective sustainable neat approach to naphtho[2,3-b]thiophene-4,9-dione: detailed mechanistic study and DFT analysis
Source: RSC Adv. 2026 Jan 28;16(7):6090–105. doi: 10.1039/d5ra08362a (PMC12851160; doi:10.1039/d5ra08362a)
Supplement: RA-016-D5RA08362A-s001 [file RA-016-D5RA08362A-s001.pdf]

Supplementary Information (SI) for RSC Advances.
This journal is © The Royal Society of Chemistry 2026

A Regioselective Sustainable Neat Approach to Naphtho[2,3-b]thiophene-4,9-dione: Detailed Mechanistic Study and DFT Analysis

Soumen K. Manik [a], Sk Asraf Ali [b,c], Nayim Sepay [d], Aniruddha Deb [e], Manik Shit [f], Paritosh Barik[b], Jyotirmoy Rath [g], Kankan K. Maity [h], Sudhir C. Pal [a], Shubhankar Samanta*[b], and Nirmal K. Hazra*[a]

[a] Department of Chemistry, Coastal Environmental Studies Research Centre, Egra Sarada Shashi Bhusan College, Vidyasagar University, Egra, Purba Medinipur, West Bengal, 721429, India.

[b] Department of Chemistry, Bidhannagar College, EB 2, Sector I, Salt Lake, Kolkata-700064, West Bengal, India.

[c] District Institute of Education and Training (DIET), Jhargram, West Bengal-721507, India

[d] Department of Chemistry, Lady Brabourne College, Kolkata-700017, West Bengal, India.

[e] Department of Chemical Engineering, Birla Institute of Technology, Mesra, Ranchi, Jharkhand, 835215, India

[f] Department of Chemistry, Jadavpur University, Jadavpur, Kolkata-700032, West Bengal, India.

[g] Department of Physics, Coastal Environmental Studies Research Centre, Egra Sarada Shashi Bhusan College, Vidyasagar University, Egra, Purba Medinipur, West Bengal, 721429, India.

[h] Department of Chemistry, Belda College, Belda, Paschim Medinipur, West Bengal-721424, India

*Corresponding authors:

Shubhankar Samanta chemshubha@gmail.com

Nirmal K Hazra nirmalkrhazra@gmail.com

Content **Page No**

Content	Page No
**1. General information and methods**	**S2**
**2. Substrate diversity**	**S2**
**2. Crystal data of compound 4c and 4´a**	**S3-S4**
**4. E-factor calculation**	**S4-S6**
**3. Experimental Section and Compound Characterization Data**	**S7-S14**
**4. Copies of ^{1}H NMR, ^{13}C NMR and HRMS spectra**	**S15-S72**
**5. DFT**	**S73-S99**

General information and methods:

Reagents, starting materials, and solvents were obtained from commercial suppliers and used without further purification. Analytical TLC was carried out on Merck silica gel GF 254 plates. The crude product was purified by column chromatography. ^{1}H NMR and ^{13}C NMR spectra were recorded on a Bruker spectrometer at 300, 400, 500, 600 MHz and 75, 101, 126, 151 MHz, respectively. Chemical shifts are reported in δ values (ppm) relative to tetramethylsilane (Me_4Si) as an internal standard, and coupling constants (J) are given in hertz (Hz). TOF MS ES+ spectra were recorded on a Bruker mass spectrometer. Melting points were determined using a melting point apparatus and were uncorrected.

Table S1: Diversity of substrate

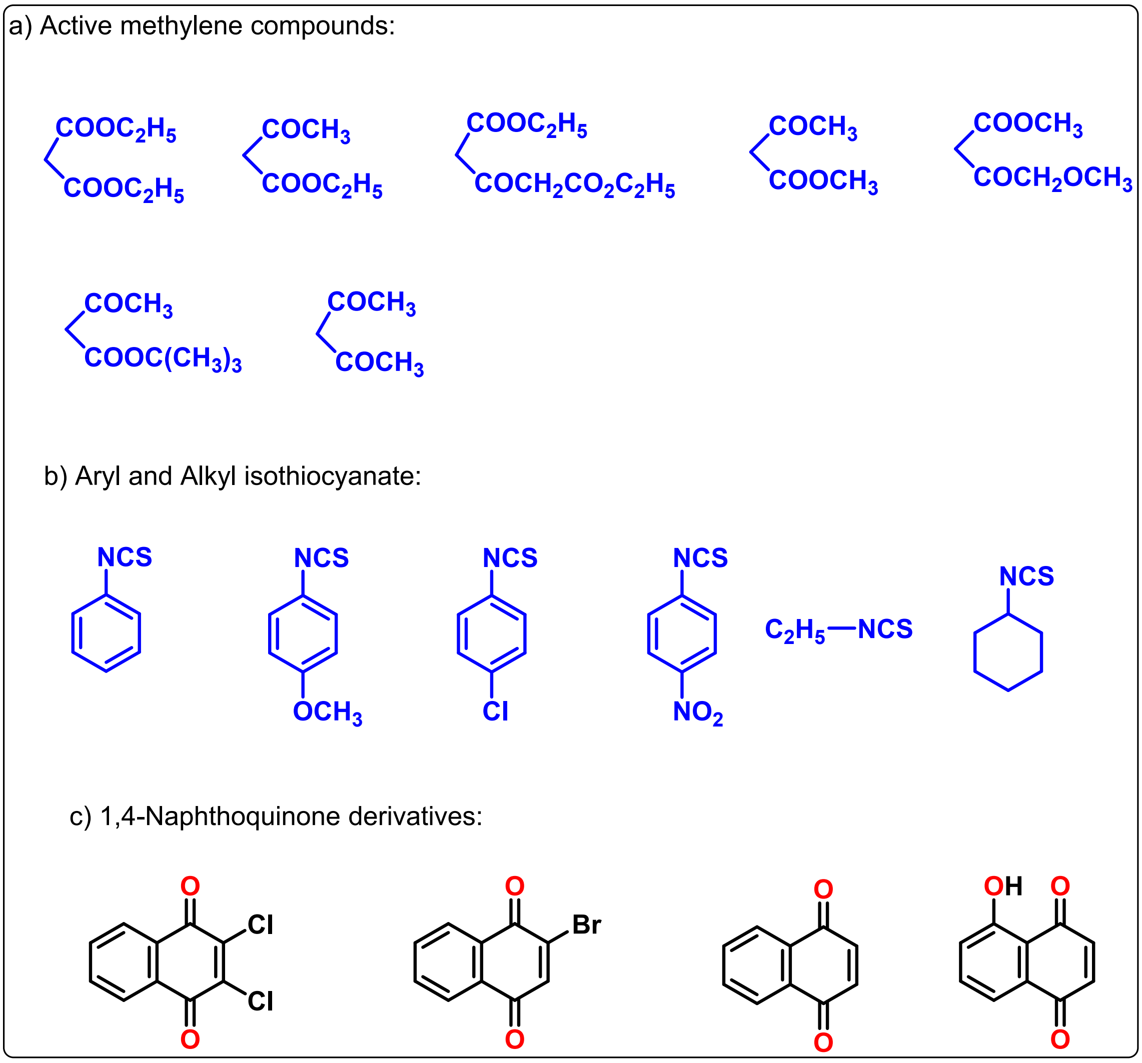

Table S2: Crystal data and refinement parameters of compounds **4´a** and **4c**

Identification Code	4´a	4c
Empirical formula	$C_{24}H_{19}NO_6S$	$C_{23}H_{19}NO_4S$
Formula weight	449.46	405.45
Temperature	296 K	293 K
Wavelength	0.71073 Å	0.71073 Å
Radiation type	Mo K\α	Mo K\α
Radiation system	Fine-focus sealed tube	Fine-focus sealed tube
Crystal system	Orthorhombic	Orthorhombic
Space group	P b c n	P c a 21
Cell length	a =13.391(3) Å<br>b =9.952(3) Å<br>c =33.797(9) Å	a = 8.7952(3) Å<br>b = 11.7758 (5) Å<br>c = 19.3258 (8) Å
Cell angle	α =90°<br>β =90°<br>γ =90°	α =90°<br>β =90°<br>γ =90°
Cell volume	4504(2)	2001.58 (14)
Density	1.326 mg/m^3	1.345 mg/m^3
Data completeness	0.996	1
Absorption correction	multi-scan	multi-scan
Refinement method	Full-matrix least-squares on F2	Full-matrix least-squares on F2
Index ranges	-16<=h<=16,<br>-12<=k<=12,<br>-40<=l<=40	-12<=h<=7,<br>-16<=k<=10,<br>-18<=l<=26
Reflection number	4171	3718
refine parameters	292	265
Theta range	2.39 - 27.11	3.46 - 29.08
Cell formula units Z	8	4
CCDC no	2329614	2466758

$^aR_1=\Sigma||F_o|-|Fc||/\Sigma|F_o|$, $^bwR_2=[\Sigma w(F_o^2-Fc^2)^2/\Sigma w(F_o^2)^2]/2$, $w=1/[\sigma^2(F_o^2)+(0.1000P)^2+0.3347P]$, where $P=(F_o^2+2Fc^2)/3$

E-factor calculation (Figure 3):

$$\text{E factor} = \frac{[\Sigma m\,(raw\ materials)\ +\Sigma m\,(reagents)\ +\ \Sigma m\,(solvents)\ +\ m\,(water)\ -\ m\,(product)]}{m(product)}$$

- Previous work:

1. Case I (Reference 20):

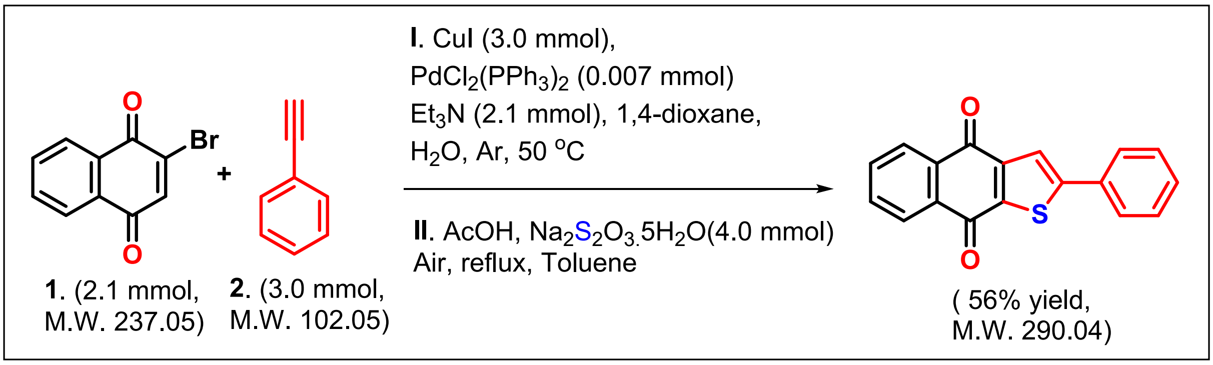

Molecular weight of substrate 1= 237.05 g/mol
Equivalent weight of substrate 1= 2.1 mmol
Molecular weight of substrate 2= 102.05 g/mol
Equivalent weight of substrate 2= 3.0 mmol
Molecular weight of CuI = 190.45
Equivalent weight of CuI = 3.0 mmol
Molecular weight of $PdCl_2(PPh_3)_2$ = 701.90
Equivalent weight of $PdCl_2(PPh_3)_2$ = 0.007 mmol
Volume of Et_3N = 0.3 ml
Density of Et_3N = 0.726 g/ml
Volume of 1,4-dioxane = 6 ml
Density of 1,4-dioxane = 1.033 g/ml
Volume of H_2O = 2 ml
Density of H_2O = 0.997 g/ml
Volume of AcOH = 20 ml
Density of AcOH = 1.05 g/ml
Molecular weight of $Na_2S_2O_3.5H_2O$ = 248.18 g/mol
Equivalent weight of $Na_2S_2O_3.5H_2O$ = 4.0 mmol
Volume of Toluene = 40 ml
Density of Toluene = 0.867 g/ml

Weight of the product = [(56/100) × 290.04 × 2.1]/ 1000
= 0.3410g

E-factor

$$= \frac{[\left(\frac{237.05 \times 2.1}{1000}\right) + \left(\frac{102.05 \times 3.0}{1000}\right) + \left(\frac{190.45 \times 3.0}{1000}\right) + \left(\frac{701.90 \times 0.007}{1000}\right) + \ldots + (6 \times 1.033) + (2 \times 0.9970) + (20 \times 1.05) + (40 \times 0. \ldots}{0.3410}$$

= 193.86

2. Case 2 (Reference 19):

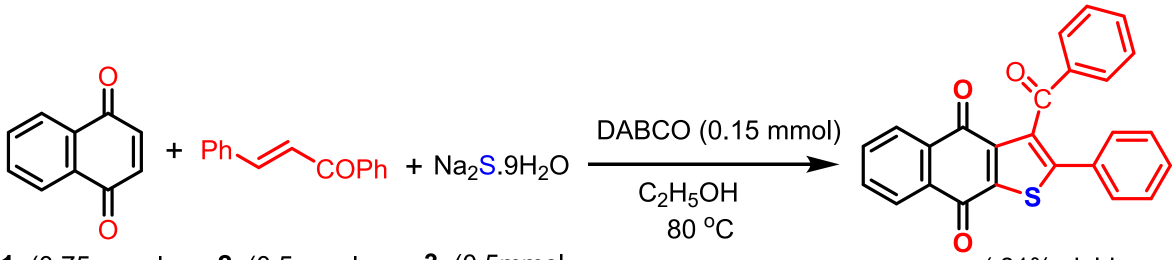

1. (0.75 mmol, M.W. 158.15) **2**. (0.5mmol, M.W. 208.09) **3**. (0.5mmol, M.W. 240.18) (81% yield, M.W. 394.44)

Molecular weight of substrate 1= 158.15
Equivalent weight of substrate 1= 0.75 mmol
Molecular weight of substrate 2 = 208.09
Equivalent weight of substrate 2= 0.5 mmol
Molecular weight of substrate 3 = 240.18
Equivalent weight of substrate 3= 0.5 mmol
Molecular weight of DABCO = 112.17
Equivalent weight of DABCO = 0.15 mmol
Volume of C_2H_5OH = 5 ml
Density of C_2H_5OH = 0.79 g/ml

Weight of the product = [(81/100) ×394.44×0.75]/1000

= 0.2396g

E-factor

$$= \frac{[\left(\frac{158.15 \times 0.75}{1000}\right) + \left(\frac{208.09 \times 0.5}{1000}\right) + \left(\frac{240.18 \times 0.5}{1000}\right) + \left(\frac{112.17 \times 0.15}{1000}\right) + (5.0 \times 0.79)] - 0.2396 \ldots}{0.2396}$$

= 16.98

3. Case 3 (Reference 21):

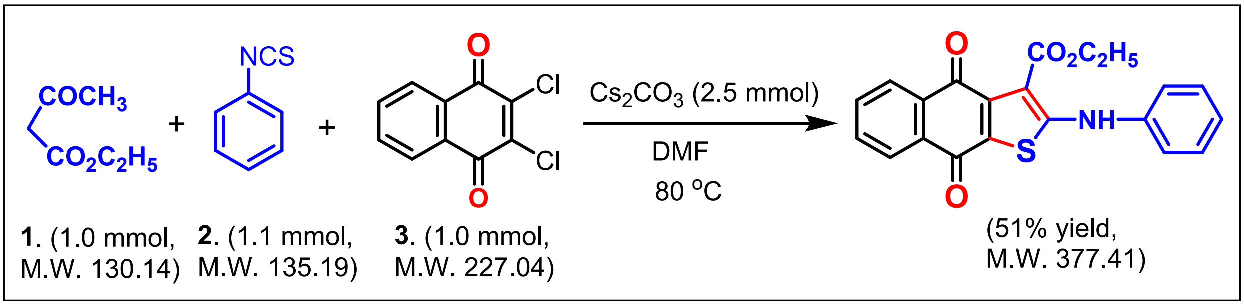

Molecular weight of substrate 1= 130.14
Equivalent weight of substrate 1= 1.0 mmol
Molecular weight of substrate 2 = 135.19
Equivalent weight of substrate 2= 1.1 mmol
Molecular weight of substrate 3 = 227.04
Equivalent weight of substrate 3= 1.0 mmol
Molecular weight of Cs_2CO_3 = 325.82
Equivalent weight of Cs_2CO_3 = 2.5 mmol
Volume of DMF = 5 ml
Density of DMF = 0.948 g/ml

Weight of the product = [(51/100) ×377.41×1]/1000

= 0.1925g

E-factor

$$= \frac{[\left(\frac{130.14 \times 1}{1000}\right) + \left(\frac{135.19 \times 1.1}{1000}\right) + \left(\frac{227.04 \times 1}{1000}\right) + \left(\frac{325.82 \times 2.5}{1000}\right) + (5.0 \times 0.948)] - 0.1925}{0.1925}$$

= 30.48

➢ Present work:

4. Case 4 (Neat approach):

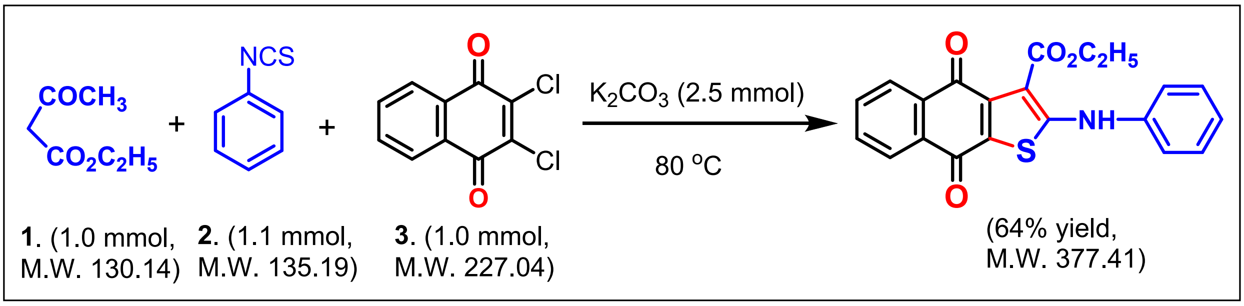

Molecular weight of substrate 1= 130.14
Equivalent weight of substrate 1= 1.0 mmol

Molecular weight of substrate 2 = 135.19
Equivalent weight of substrate 2= 1.1 mmol
Molecular weight of substrate 3 = 227.04
Equivalent weight of substrate 3= 1.0 mmol
Molecular weight of K_2CO_3 = 138.21
Equivalent weight of K_2CO_3 = 2.5 mmol

Weight of the product = [(64/100) ×377.41×1]/1000

= 0.2415g

$$\text{E-factor} = \frac{\left[\left(\frac{130.14 \times 1}{1000}\right) + \left(\frac{135.19 \times 1.1}{1000}\right) + \left(\frac{227.04 \times 1}{1000}\right) + \left(\frac{138.21 \times 2.5}{1000}\right)\right] - 0.2415}{0.2415}$$

= 2.52

Experimental Section and Compound Characterization Data:

Initially, K_2CO_3 (2.5 mmol) was added to an oven-dry hard glass test tube with a glass rod. Then, an active methylene compound (1 mmol) and an alkyl/aryl isothiocyanate (1.1 mmol) were added to the test tube. The test tube was heated at 80 °C for 30 minutes in an oil bath. Then, 2,3-dichloro-1,4-naphthoquinone / 2-bromo-1,4-naphthoquinone / 1,4-naphthoquinone was added to the test tube and heated for an additional 30 minutes. The progress of the reaction was monitored by thin-layer chromatography (TLC) using a 1.5:8.5 (v/v) EtOAc: Hexane mixture as the mobile phase. Once the 1,4-quinone derivatives are consumed, as indicated by TLC, the reaction mixture is brought to room temperature. The reaction mixture was acidified with dilute HCl, extracted with ethyl acetate, followed by washing with brine water. The organic layer was dried over sodium sulfate (Na_2SO_4). After evaporating the solvent, the crude product was purified by column chromatography on silica gel (100–200 mesh) using an ethyl acetate-petroleum ether (1:20) eluent to get the pure desired products **4a** – **4u**.

Diethyl (Z)-4,9-dioxo-2-(phenylimino)-4,9-dihydronaphtho[2,3-b]thiophene-3,3(2H)-dicarboxylate (4´a)

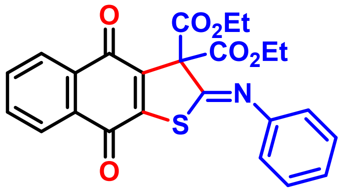

Purified by silica gel column chromatography (eluent: petroleum ether/ethyl acetate = 20:1 to 12:1); yellow crystalline solid (71%, 31.8mg), m.p. 108 – 110 °C, ^1H NMR (300 MHz, $CDCl_3$) δ 8.17 – 8.09 (m, 2H), 7.83 –

7.71 (m, 2H), 7.43-7.28 (m, 2H), 7.25-7.21 (m, 1H), 7.01 – 6.97 (m, 2H), 4.42-4.31 (m, 4H), 1.31 (t, J = 7.2 Hz, 6H). ^{13}C NMR (75 MHz, $CDCl_3$) δ 179.5, 177.1, 163.8, 159.0, 153.6, 149.9, 138.1, 134.9 (2C), 133.8 (2C), 132.6, 132.0, 129.6 (2C), 127.4, 126.9, 126.6, 119.8 (2C), 63.5 (2C), 14.1 (2C). LCMS (ESI) m/z : 450.1046.

Ethyl 4,9-dioxo-2-(phenylamino)-4,9-dihydronaphtho[2,3-b]thiophene-3-carboxylate (4a)

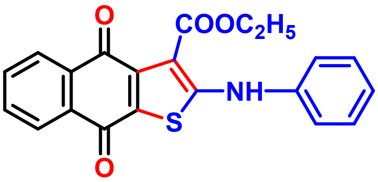

Purified by silica gel column chromatography (eluent: petroleum ether/ethyl acetate = 30:1 to 20:1); red crystalline solid, 72 % yield, 27.1 mg; m.p. 142-144 °C, ^{1}H NMR (400 MHz, $CDCl_3$) δ 10.05 (s, 1H), 8.15 – 8.11 (m, 2H), 7.72 – 7.70 (m, 2H), 7.49 – 7.45 (m, 2H), 7.41 - 7.39 (m, 2H), 7.27 – 7.23 (m, 1H), 4.46 (q, J = 7.1 Hz, 2H), 1.47 (t, J = 7.1 Hz, 3H). ^{13}C NMR (101 MHz, $CDCl_3$) δ 178.6, 177.5, 165.9, 163.9, 141.6, 139.3, 134.6, 133.4, 133.1, 132.6, 130.0 (2C), 129.6, 127.3, 125.8, 125.6, 120.7 (2C), 107.3, 61.4, 14.1. HRMS (ESI) *m/z*: [M + Na]$^+$ calcd for $C_{21}H_{15}NO_4S$: 400.0722 found 400.0721.

Methyl 4,9-dioxo-2-(phenylamino)-4,9-dihydronaphtho[2,3-b]thiophene-3-carboxylate (4b)

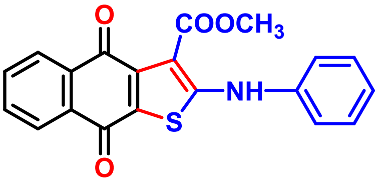

Purified by silica gel column chromatography (eluent: petroleum ether/ethyl acetate = 30:1 to 20:1); red crystalline solid, 71% yield, 25.7 mg; m.p. 125-127 °C, ^{1}H NMR (600 MHz, $CDCl_3$) δ 10.05 (s, 1H), 8.15 – 8.12 (m, 2H), 7.73 – 7.70 (m, 2H), 7.49 – 7.46 (m, 2H), 7.41 – 7.39 (m, 2H), 7.26 – 7.24 (m, 1H), 4.00 – 3.99 (m, 3H). ^{13}C NMR (151 MHz, $CDCl_3$) δ 178.5, 177.5, 166.4, 164.2, 141.5, 139.3, 134.5, 133.4, 133.2, 132.6, 130.0 (2C), 127.4, 125.8, 125.8, 120.9 (2C), 106.7, 52.2. HRMS (ESI) *m/z*: [M - H]$^+$ calcd for $C_{20}H_{12}NO_4S$: 362.0485 found 362.0482.

Tert-butyl 4,9-dioxo-2-(phenylamino)-4,9-dihydronaphtho[2,3-b]thiophene-3-carboxylate (4c)

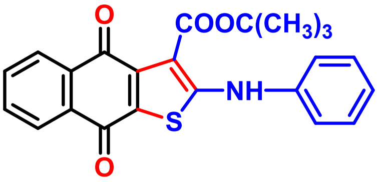

Purified by silica gel column chromatography (eluent: petroleum ether/ethyl acetate = 30:1 to 20:1); red crystalline solid, 77% yield, 31.2 mg; m.p. 152-154 °C, ^{1}H NMR (400 MHz, $CDCl_3$) δ 10.01 (s, 1H), 8.14 – 8.11 (m, 2H), 7.72 – 7.69 (m, 2H), 7.49 – 7.44 (m, 2H), 7.41 – 7.39 (m, 2H), 7.26 – 7.22 (m, 1H), 1.67 (s, 9H). ^{13}C NMR (151 MHz, $CDCl_3$) δ 178.7, 177.5, 165.2, 163.2, 142.0, 139.4, 134.8, 133.3, 133.0, 132.7, 129.9 (2C), 129.1, 128.4, 127.1, 125.8, 125.3, 120.4 (2C), 109.2, 82.7, 28.2. HRMS (ESI) *m/z*: $[M - H]^+$ calcd for $C_{23}H_{18}NO_4S$: 404.0955 found 404.0947.

3-Acetyl-2-(phenylamino)naphtho[2,3-b]thiophene-4,9-dione (4d)

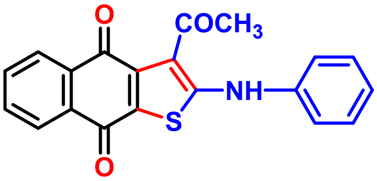

Purified by silica gel column chromatography (eluent: petroleum ether/ethyl acetate = 30:1 to 20:1); red crystalline solid, 65% yield, 22.5 mg; m.p. 158-160 °C, ^{1}H NMR (400 MHz, $CDCl_3$) δ 11.45 (s, 1H), 8.17 – 8.13 (m, 2H), 7.76 – 7.74 (m, 2H), 7.50 – 7.47 (m, 2H), 7.43 – 7.41 (m, 2H), 7.31 – 7.30 (m, 1H), 2.71 (s, 3H). ^{13}C NMR (101 MHz, $CDCl_3$) δ 198.4, 180.5, 177.7, 164.9, 141.2, 139.2, 134.2, 133.6, 133.5, 132.6, 130.0 (2C), 127.4, 126.1, 125.9, 121.4 (2C), 115.8, 31.6. LCMS (ESI) m/z: 348.0681.

Ethyl 2-((4-methoxyphenyl)amino)-4,9-dioxo-4,9-dihydronaphtho[2,3-b]thiophene-3-carboxylate (4e)

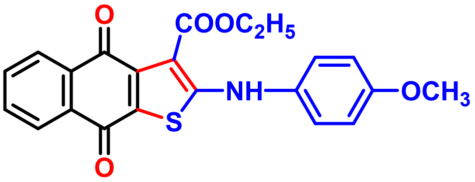

Purified by silica gel column chromatography (eluent: petroleum ether/ethyl acetate = 30:1 to 20:1); red solid, 75% yield, 30.5 mg; m.p. 128-130 °C, ^{1}H NMR (400 MHz, $CDCl_3$) δ 9.75 (s, 1H), 8.14 – 8.09 (m, 2H), 7.71 – 7.68 (m, 2H), 7.33 – 7.31 (m, 2H), 7.01 – 6.97 (m, 2H), 4.45 (q, J = 7.1 Hz, 2H), 3.87 (s, 3H), 1.46 (t, J = 7.1 Hz, 3H). ^{13}C NMR (101 MHz, $CDCl_3$) δ 178.6, 177.4, 166.5, 165.9, 158.0, 142.0, 134.6, 133.2, 132.9, 132.6, 132.5, 129.2, 127.2, 125.7, 123.9(2C), 115.2(2C), 106.1, 61.2, 55.6, 14.1. Mass $[M]^+$: 407.2591

Methyl 2-((4-methoxyphenyl)amino)-4,9-dioxo-4,9-dihydronaptho[2,3-b]thiophene-3-carboxylate (4f)

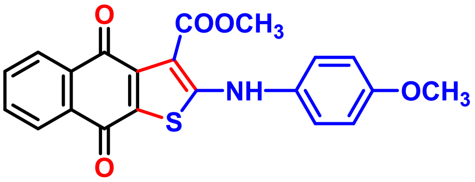

Purified by silica gel column chromatography (eluent: petroleum ether/ethyl acetate = 30:1 to 20:1); red solid, 72% yield, 28.3 mg; m.p. 112-114 °C, ^{1}H NMR (400 MHz, $CDCl_3$) δ 9.76 (s, 1H), 8.14 – 8.10 (m, 2H), 7.72 – 7.69 (m, 2H), 7.34 – 7.32 (m, 2H), 7.01 – 6.98 (m, 2H), 3.99 (s, 3H), 3.87 (s, 3H). ^{13}C NMR (151 MHz, $CDCl_3$) δ 178.9, 177.5, 166.8, 166.4, 158.1, 141.8, 134.6, 133.3, 133.1, 132.6, 132.4, 129.4, 127.3, 125.7, 124.1(2C), 115.2(2C), 105.5, 55.6, 52.1. HRMS (ESI) *m/z*: $[M - H]^+$ calcd for $C_{21}H_{14}NO_5S$: 392.0591 found 392.0595.

Tert-butyl 2-((4-methoxyphenyl)amino)-4,9-dioxo-4,9-dihydronaphtho[2,3b]thiophene-3-carboxylate (4g)

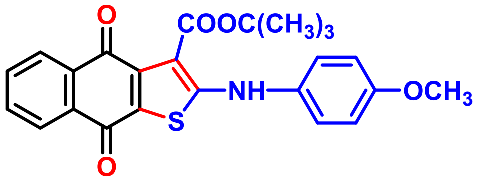

Purified by silica gel column chromatography (eluent: petroleum ether/ethyl acetate = 30:1 to 20:1); red solid, 81% yield, 35.2 mg; m.p. 136-138 °C, ^{1}H NMR (400 MHz, $CDCl_3$) δ 9.72 (s, 1H), 8.11 - 8.08 (m, 2H), 7.69 - 7.67 (m, 2H), 7.33 – 7.30 (m, 2H), 6.99 – 6.96 (m, 2H), 3.86 – 3.85 (m, 3H), 1.66 (s, 9H). ^{13}C NMR (151 MHz, $CDCl_3$) δ 178.8, 177.4, 165.8, 165.3, 157.8, 142.3, 134.8, 133.1, 132.9, 132.7, 132.6, 128.6, 127.0, 125.7, 123.6(2C), 115.0(2C), 107.9, 82.4, 55.6, 28.2(3C). HRMS (ESI) *m/z*: $[M - H]^+$ calcd for $C_{24}H_{20}NO_5S$: 434.1060 found 434.1061.

3-Acetyl-2-((4-methoxyphenyl)amino)naphtho[2,3-b]thiophene-4,9-dione (4h)

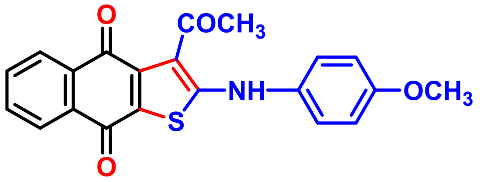

Purified by silica gel column chromatography (eluent: petroleum ether/ethyl acetate = 30:1 to 20:1); red solid, 67% yield, 25.2 mg; m.p. 170-172 °C, ^{1}H NMR (300 MHz, $CDCl_3$) δ 11.15 (s, 1H), 8.12 – 8.07 (m, 2H), 7.71 – 7.68 (m, 2H), 7.31 – 7.28 (m, 2H), 6.97 – 6.95 (m, 2H), 3.84 (s, 3H), 2.67 (s, 3H). ^{13}C NMR (75 MHz, $CDCl_3$) δ 198.2, 180.5, 177.7, 167.4, 141.6, 134.3, 133.5(3C), 132.7, 132.4, 127.4, 125.9, 124.4(3C), 115.2(2C), 115.0, 55.7, 31.6. Mass $[M]^+$: 377.8676.

Ethyl 2-((4-chlorophenyl)amino)-4,9-dioxo-4,9-dihydronaphtho[2,3-b]thiophene-3-carboxylate (4i)

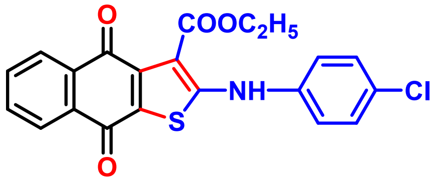

Purified by silica gel column chromatography (eluent: petroleum ether/ethyl acetate = 30:1 to 20:1); red solid, 70% yield, 28.7 mg; m.p. 168-170 °C, ^{1}H NMR (300 MHz, $CDCl_3$) δ 10.04 (s, 1H), 8.16 – 8.12 (m, 2H), 7.74 – 7.70 (m, 2H), 7.45 – 7.42 (m, 2H), 7.35 – 7.32 (m, 2H), 4.46 (q, J = 7.1 Hz, 2H), 1.46 (t, J = 7.1 Hz, 3H). ^{13}C NMR (75 MHz, $CDCl_3$) δ 178.5, 177.5, 165.9, 163.3, 141.5, 137.9, 134.5, 133.5, 133.1, 132.5, 130.7, 130.0(2C), 129.0, 127.4, 125.8, 121.9(2C), 107.7, 61.5, 14.1. Mass [M+ H]$^+$: 412.4849.

Methyl 2-((4-chlorophenyl)amino)-4,9-dioxo-4,9-dihydronaphtho[2,3-b]thiophene-3-carboxylate (4j)

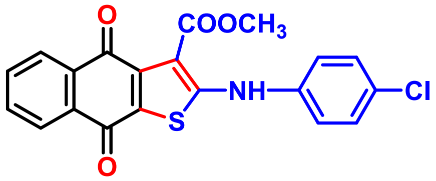

Purified by silica gel column chromatography (eluent: petroleum ether/ethyl acetate = 30:1 to 20:1); red solid, 69% yield, 27.3 mg; m.p. 152-154 °C, ^{1}H NMR (400 MHz, $CDCl_3$) δ 10.03 (s, 1H), 8.14 – 8.11 (m, 2H), 7.73 – 7.71 (m, 2H), 7.45 – 7.41 (m, 2H), 7.35 – 7.32 (m, 2H), 3.99 (s, 3H). ^{13}C NMR (151 MHz, $CDCl_3$) δ 178.6, 177.5, 166.4, 163.7, 141.4, 137.9, 134.5, 133.5, 133.2, 132.5, 130.9, 130.0(2C), 129.2, 127.4, 125.9, 122.1(2C), 107.1, 52.3. HRMS (ESI) m/z: [M -H]$^+$ calcd for $C_{20}H_{11}ClNO_4S$: 396.0096 found 396.0091.

Tert-butyl 2-((4-chlorophenyl)amino)-4,9-dioxo-4,9-dihydronaphtho[2,3-b]thiophene-3-carboxylate (4k)

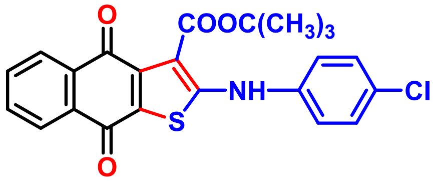

Purified by silica gel column chromatography (eluent: petroleum ether/ethyl acetate = 30:1 to 20:1); red solid, 73% yield, 32.0 mg; m.p. 174-176 °C, ^{1}H NMR (600 MHz, $CDCl_3$) δ 10.02 (s, 1H), 8.14 – 8.11 (m, 2H), 7.72 – 7.70 (m, 2H), 7.43 – 7.41 (m, 2H), 7.34 – 7.32 (m, 2H), 1.66 (s, 9H). ^{13}C NMR (151 MHz, $CDCl_3$) δ 178.5, 177.5, 165.2, 162.7, 141.9, 138.0, 134.7, 133.4, 133.0, 132.6, 130.0(2C), 128.7, 127.1, 125.8, 123.9, 121.6(2C), 109.5, 82.9, 28.1(3C). HRMS (ESI) m/z: [M + Na]$^+$ calcd for $C_{23}H_{18}ClNO_4S$: 462.0542 found 462.0531.

3-Acetyl-2-((4-chlorophenyl)amino)naphtho[2,3-b]thiophene-4,9-dione (4l)

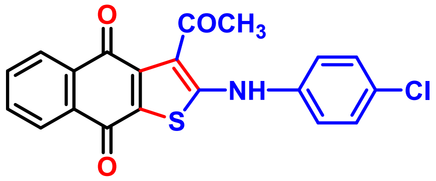

Purified by silica gel column chromatography (eluent: petroleum ether/ethyl acetate = 30:1 to 20:1); red solid, 62% yield, 23.6 mg; m.p. 148-150 °C, ^{1}H NMR (500 MHz, $CDCl_3$) δ 11.42 (s, 1H), 8.17 – 8.14 (m, 2H), 7.77 – 7.75 (m, 2H), 7.45 – 7.44 (m, 2H), 7.37 – 7.35 (m, 2H), 2.71 (s, 3H). ^{13}C NMR (126 MHz, $CDCl_3$) δ 198.5, 180.1,

177.6, 164.4, 141.1, 137.7, 134.2, 133.6 ,132.5, 131.3, 130.4, 130.1(2C), 129.0, 127.5, 125.9, 122.6(2C), 116.1, 31.6. Mass $[M+Na+H]^+$: 405.2555.

Ethyl 2-((4-nitrophenyl)amino)-4,9-dioxo-4,9-dihydronaphtho[2,3-b]thiophene-3-carboxylate (4m)

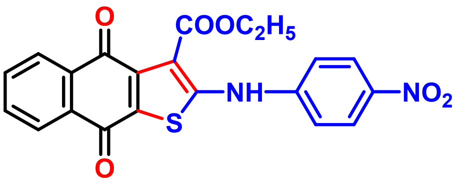

Purified by silica gel column chromatography (eluent: petroleum ether/ethyl acetate = 30:1 to 15:1); orange solid, 49% yield, 20.6 mg; m.p. 180-182 °C, ^{1}H NMR (400 MHz, $CDCl_3$) δ 10.53 (s, 1H), 8.37 – 8.33 (m, 2H), 8.19 – 8.17 (m, 2H), 7.78 – 7.75 (m, 2H), 7.50 – 7.48 (m, 2H), 4.50 (q, J = 7.2 Hz, 2H), 1.48 (t, J = 7.1 Hz, 3H). ^{13}C NMR (101 MHz, CDCl3) δ 178.1, 177.2, 169.9, 165.7, 159.1, 144.6, 143.3, 134.5, 133.8, 133.3, 132.4, 131.1, 127.5, 126.1, 126.0(2C), 118.1(2C), 110.8, 61.9, 14.0. Mass $[M+Na]^+$: 445.1604.

Ethyl 2-(ethylamino)-4,9-dioxo-4,9-dihydronaphtho[2,3-b]thiophene-3-carboxylate (4n)

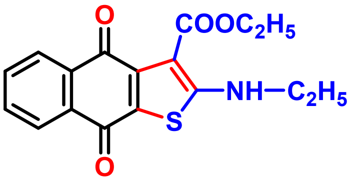

Purified by silica gel column chromatography (eluent: petroleum ether/ethyl acetate = 30:1 to 20:1); deep red solid, 72% yield, 23.6 mg; m.p. 125 – 127 °C, ^{1}H NMR (500 MHz,) δ 8.12 – 8.11 (m, 2H), 8.09 (s, 1H), 7.70 – 7.68 (m, 2H), 4.40 (q, J = 7.1 Hz, 2H), 3.44 – 3.39 (m, 2H), 1.45 – 1.41 (m, 6H). ^{13}C NMR (126 MHz, CDCl3) δ 178.9, 177.2, 169.1, 165.8, 142.8, 134.7, 133.0, 132.9, 132.7, 129.0, 127.2, 125.6, 103.8, 60.9, 42.6, 29.7, 14.2. Mass $[M + H]^+$: 330.1377.

Methyl 2-(ethylamino)-4,9-dioxo-4,9-dihydronaphtho[2,3-b]thiophene-3-carboxylate (4o)

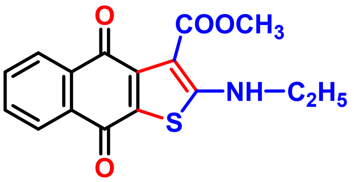

Purified by silica gel column chromatography (eluent: petroleum ether/ethyl acetate = 30:1 to 20:1); deep red solid, 70% yield, 22.0 mg; m.p. 120 – 122 °C, ^{1}H NMR (400 MHz, $CDCl_3$) δ 8.11 – 8.09 (m, 3H,), 7.69 - 7.67 (m, 2H), 3.92 (s, 3H), 3.44 – 3.37 (m, 2H), 1.44 - 1.40 (m, 3H). ^{13}C NMR (151 MHz, $CDCl_3$) δ 179.0, 177.2,

169.3, 166.3, 142.6, 134.6, 133.2, 133.0, 132.6, 129.1, 127.3, 125.7, 103.3, 51.8, 42.7, 14.0. HRMS (ESI) *m/z*: $[M - H]^+$ calcd for $C_{16}H_{12}NO_4S$: 314.0485 found 314.0487.

Tert-butyl 2-(ethylamino)-4,9-dioxo-4,9-dihydronaphtho[2,3-b]thiophene-3-carboxylate (4p)

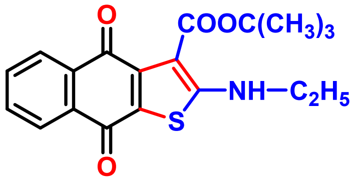

Purified by silica gel column chromatography (eluent: petroleum ether/ethyl acetate = 30:1 to 20:1); deep red solid, 73% yield, 26.0 mg; m.p. 134 - 136 °C, ^{1}H NMR (600 MHz, $CDCl_3$) δ 8.11 – 8.06 (m, 2H), 7.99 – 7.98 (m, 1H), 7.68 – 7.65 (m, 2H), 3.41-3.36 (m, 2H), 1.62 (s, 9H), 1.43 – 1.40 (m, 3H). ^{13}C NMR (151 MHz, $CDCl_3$) δ 178.9, 177.1, 168.8, 165.1, 143.3, 134.9, 133.0, 132.8, 132.8, 128.3, 127.0, 125.6, 105.6, 81.9, 42.6, 28.3(3C), 14.06. HRMS (ESI) *m/z*: $[M - H]^+$ calcd for $C_{19}H_{18}NO_4S$: 356.0955 found 356.0949.

3-Acetyl-2-(ethylamino)naphtho[2,3-b]thiophene-4,9-dione (4q)

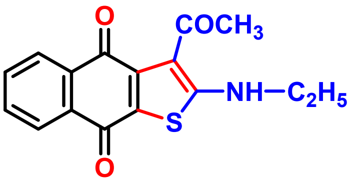

Purified by silica gel column chromatography (eluent: petroleum ether/ethyl acetate = 30:1 to 20:1); deep red solid, 65% yield, 19.4 mg; m.p. 110 – 112 °C, ^{1}H NMR (500 MHz,) δ 9.56 (s, 1H), 8.14 – 8.11 (m, 2H), 7.73 – 7.71 (m, 2H), 3.45 – 3.39 (m, 2H), 2.63 (s, 3H), 1.43 (t, J = 7.3 Hz, 3H). ^{13}C NMR (126 MHz, $CDCl_3$) δ 197.5, 180.5, 177.3, 169.7, 142.2, 134.3, 133.4, 133.3, 132.7, 129.4, 127.3, 125.7, 113.2, 42.8, 31.4, 13.9. Mass $[M+H]^+$: 300.3356.

Ethyl 2-(cyclohexylamino)-4,9-dioxo-4,9-dihydronaphtho[2,3-b]thiophene-3-carboxylate (4r)

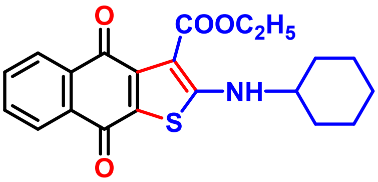

Purified by silica gel column chromatography (eluent: petroleum ether/ethyl acetate = 30:1 to 20:1); red solid, 64% yield, 24.5 mg; m.p. 126 °C – 128 °C, ^{1}H NMR (500 MHz,) δ 8.21 – 8.19 (m, 1H), 8.11 – 8.09 (m, 2H), 7.69 – 7.67 (m, 2H), 4.39 (q, J = 7.1 Hz, 2H), 3.36 – 3.33 (m, 1H), 2.14 – 2.11 (m, 2H), 1.84 – 1.81 (m, 2H), 1.69 – 1.67 (m, 2H), 1.48 – 1.46 (m, 1H), 1.44 – 1.41 (m, 6H). ^{13}C NMR (126 MHz, $CDCl_3$) δ 178.8, 177.2, 168.0, 165.9, 142.9, 134.7, 133.0, 132.9, 132.7, 128.8, 127.2, 125.6, 103.7, 60.8, 57.3, 32.1(2C), 25.3, 24.4(2C), 14.2. Mass$[M+H]^+$: 384.4041.

Methyl 2-(cyclohexylamino)-4,9-dioxo-4,9-dihydronaphtho[2,3-b]thiophene-3-carboxylate (4s)

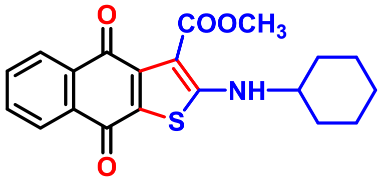

Purified by silica gel column chromatography (eluent: petroleum ether/ethyl acetate = 30:1 to 20:1); red solid, 61% yield, 22.5 mg; m.p. 118 - 120 °C, ^{1}H NMR (600 MHz,) δ 8.25 – 8.24 (m, 1H), 8.11 – 8.09 (m, 2H), 7.69 – 7.67 (m, 2H), 3.92 (s, 3H), 3.36 – 3.33 (m, 1H), 2.15- 2.11 (m, 2H), 1.85- 1.80 (m, 2H), 1.73 – 1.67 (m, 3H), 1.46 – 1.42 (m, 3H). ^{13}C NMR (151 MHz, $CDCl_3$) δ 179.0, 177.2, 168.1, 166.4, 142.7, 134.6, 133.1, 133.0, 132.6, 129.0, 127.3, 125.6, 103.1, 57.3, 51.8, 32.1(2C), 25.3, 24.4(2C). HRMS (ESI) *m*/*z*: $[M - H]^+$ calcd for $C_{20}H_{18}NO_4S$: 368.0955 found 368.0950.

Tert-butyl 2-(cyclohexylamino)-4,9-dioxo-4,9-dihydronaphtho[2,3-b]thiophene-3-carboxylate (4t)

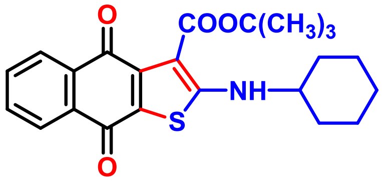

Purified by silica gel column chromatography (eluent: petroleum ether/ethyl acetate = 30:1 to 20:1); red solid, 68% yield, 27.9 mg; m.p. 138 - 140 °C, ^{1}H NMR (600 MHz, $CDCl_3$) δ 8.10 - 8.08 (m, 1H), 8.07 – 8.05 (m, 2H), 7.67 – 7.65 (m, 2H), 3.34 – 3.28 (m, 1H), 2.14 – 2.12 (m, 2H), 1.83 – 1.81 (m, 2H), 1.62 – 1.61 (m, 9H), 1.44 – 1.41 (m, 3H), 1.30 – 1.27 (m, 3H). ^{13}C NMR (151 MHz, $CDCl_3$) δ 178.9, 177.1, 167.6, 165.1, 143.4, 134.9, 132.9(2C), 128.1, 127.0, 125.6, 105.5, 81.8, 57.3, 32.2(2C), 29.7, 28.2(3C), 25.3, 24.5(2C). HRMS (ESI) *m*/*z*: $[M - H]^+$ calcd for $C_{23}H_{24}NO_4S$: 410.1424 found 410.1419.

3-Acetyl-2-(cyclohexylamino)naphtho[2,3-b]thiophene-4,9-dione (4u)

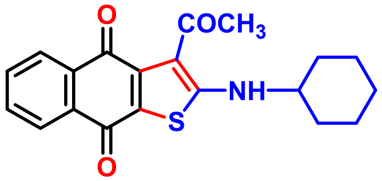

Purified by silica gel column chromatography (eluent: petroleum ether/ethyl acetate = 30:1 to 20:1); deep red solid, 63% yield, 22.2 mg; m.p. 117 -119 °C, ^{1}H NMR (500 MHz, $CDCl_3$) δ 9.74 – 9.72 (m, 1H), 8.14 – 8.11 (m, 2H), 7.73 – 7.71 (m, 2H), 3.39 – 3.33 (m, 1H), 2.63 (s, 3H), 2.14 – 2.10 (m, 2H), 1.86 – 1.81 (m, 2H), 1.71 (s, 1H), 1.49 – 1.44 (m, 2H), 1.28 – 1.27 (m, 3H). ^{13}C NMR (126 MHz, $CDCl_3$) δ 197.4, 180.5, 177.4, 168.5, 142.3, 134.3, 133.3, 133.2, 132.7, 127.3, 125.7, 113.1, 59.5, 38.2, 32.0, 31.2, 29.7(2), 29.4, 22.7. HRMS (ESI) *m*/*z*: $[M + H]^+$ calcd for $C_{20}H_{20}NO_3S$: 354.1166 found 354.1169.

Ethyl 5-hydroxy-4,9-dioxo-2-(phenylamino)-4,9-dihydronaphtho[2,3-b]thiophene-3-carboxylate (4v)

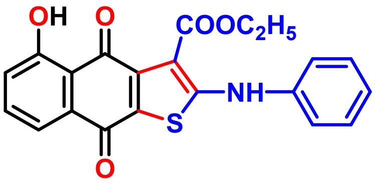

Purified by silica gel column chromatography (eluent: petroleum ether/ethyl acetate = 30:1 to 20:1); red crystalline solid, 81% yield, 31.8 mg, m.p. 148 – 150 °C, ^{1}H NMR (600 MHz,) δ 12.06 (s, 1H), 10.08 (s, 1H), 7.64 – 7.63 (m, 1H), 7.57 – 7.54 (m, 1H), 7.48 – 7.46 (m, 1H), 7.39 – 7.38 (m, 2H), 7.27 – 7.23 (m, 2H), 7.20 – 7.18 (m, 1H), 4.47 – 4.43 (m, 2H), 1.47 – 1.44 (m, 3H). ^{13}C NMR (101 MHz, $CDCl_3$) δ 182.36, 177.94, 165.73, 164.38, 161.06, 142.56, 139.18, 135.59, 134.64, 129.98(2C), 125.82, 123.63, 120.79(2C), 120.73, 120.00, 115.11, 107.80, 61.49, 14.08. HRMS (ESI) *m*/*z*: $[M + H]^+$ calcd for $C_{21}H_{16}NO_5S$: 394.0751 found 394.0750.

^{1}H NMR (300MHz, $CDCl_3$)

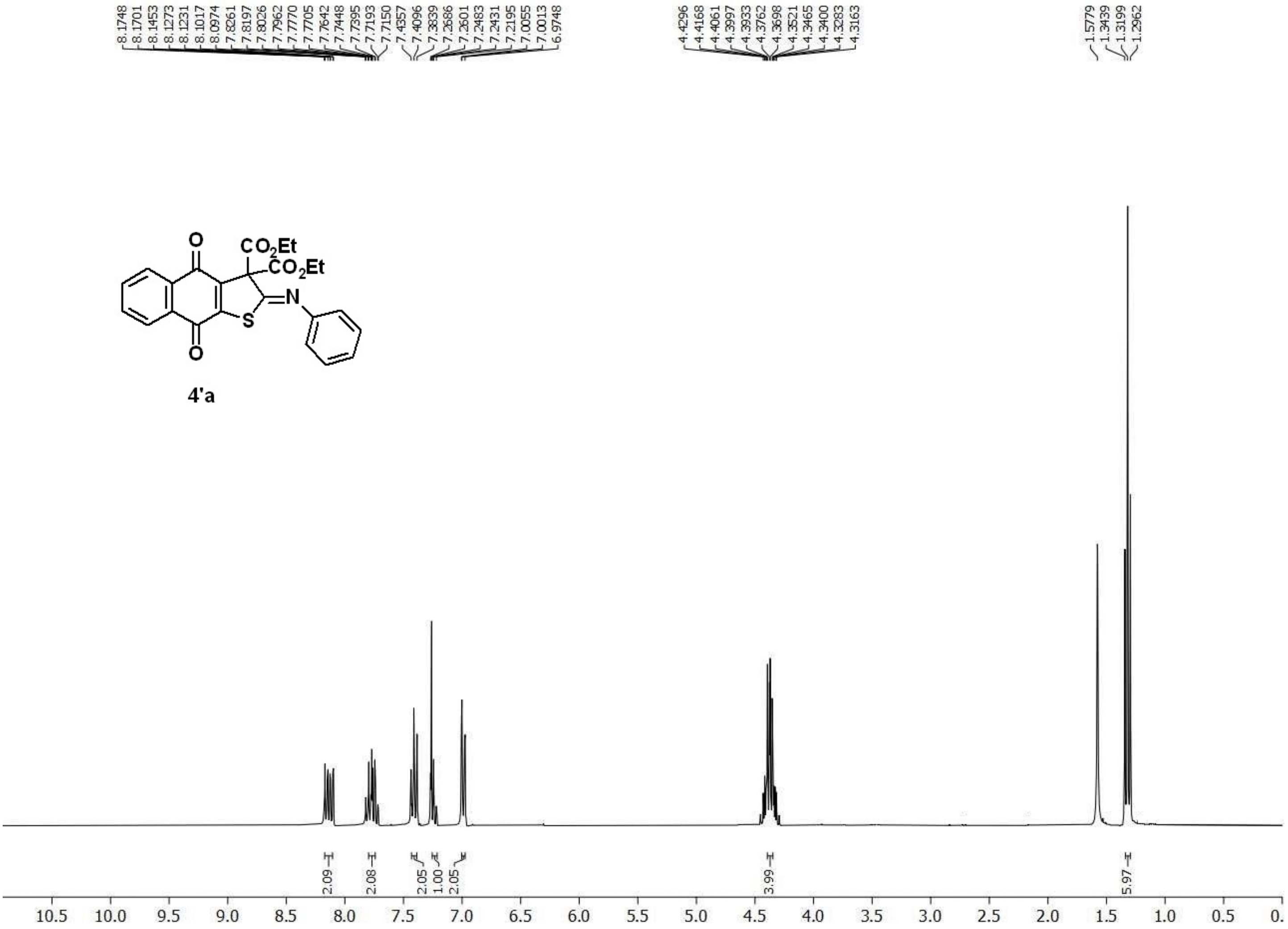

^{13}C NMR (75MHz, $CDCl_3$)

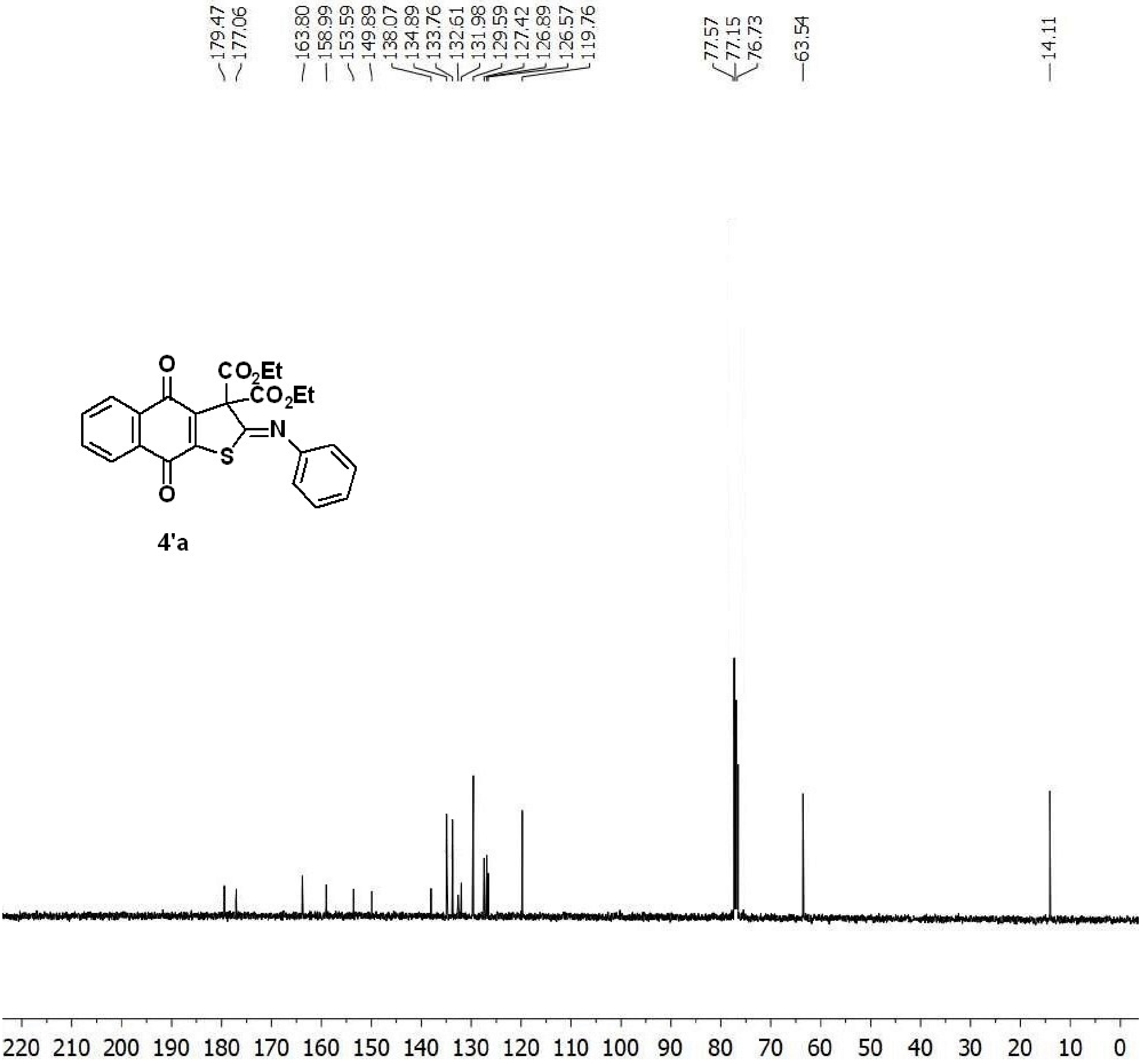

^{1}H NMR (400MHz, $CDCl_3$)

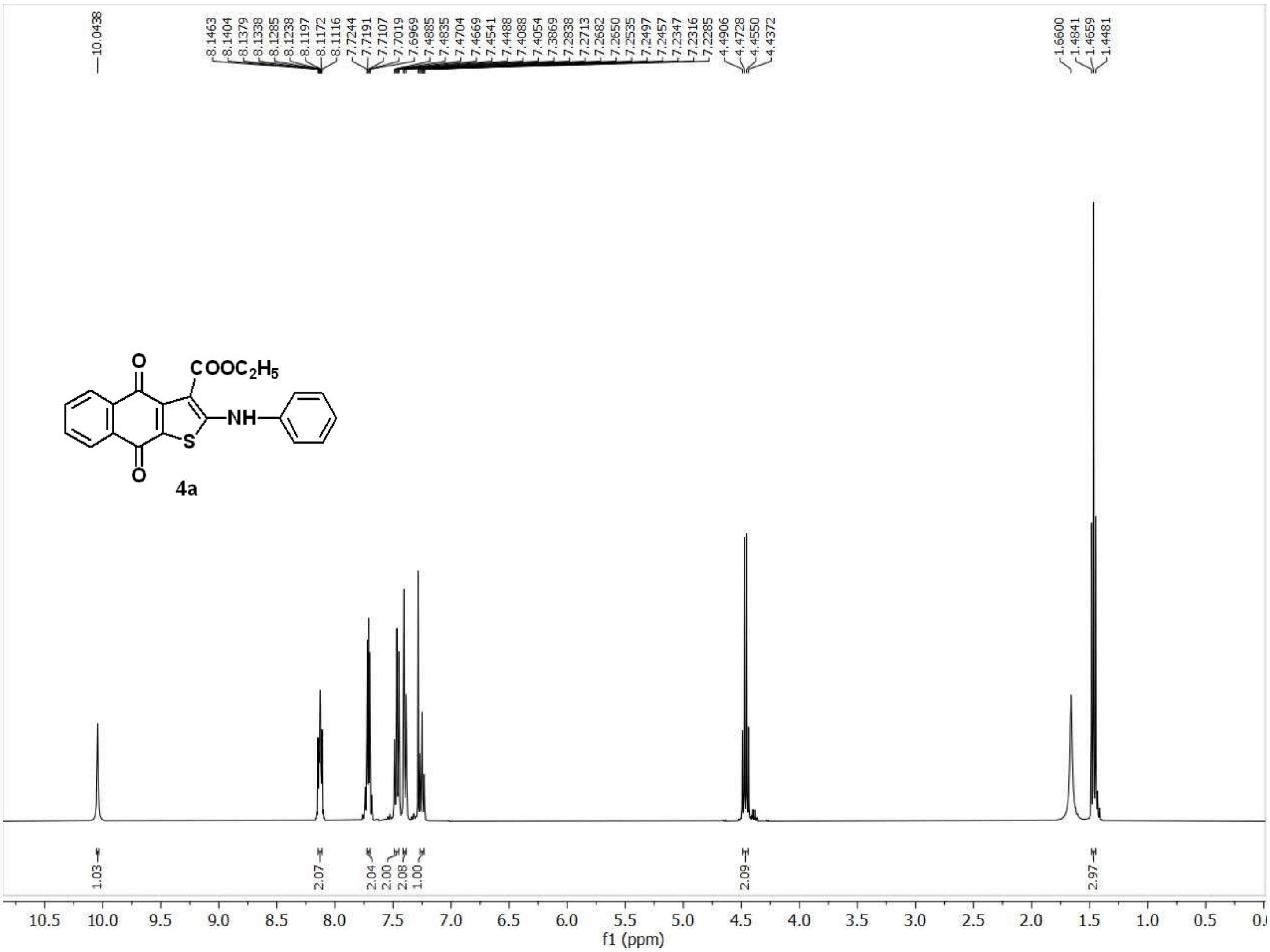

^{13}C NMR (100MHz, $CDCl_3$)

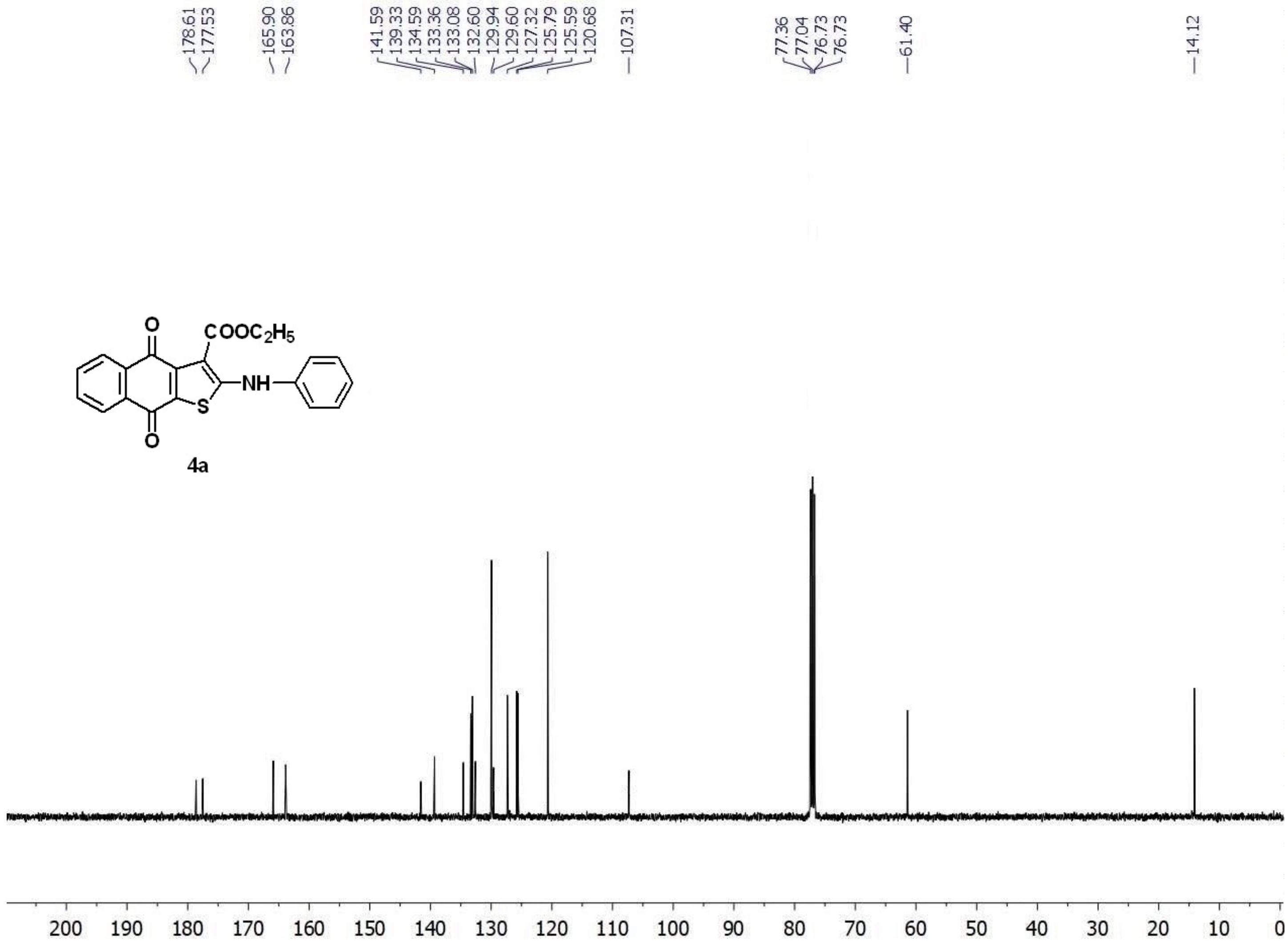

178.61
177.53
165.90
163.86
141.59
139.33
134.59
133.36
133.08
132.60
129.94
129.60
127.32
125.79
125.59
120.68
107.31
77.36
77.04
76.73
76.73
61.40
14.12
COOC2H5
NH
S
O
4a
200
190
180
170
160
150
140
130
120
110
100
90
80
70
60
50
40
30
20
10
0

^{1}H NMR (600MHz, $CDCl_3$)

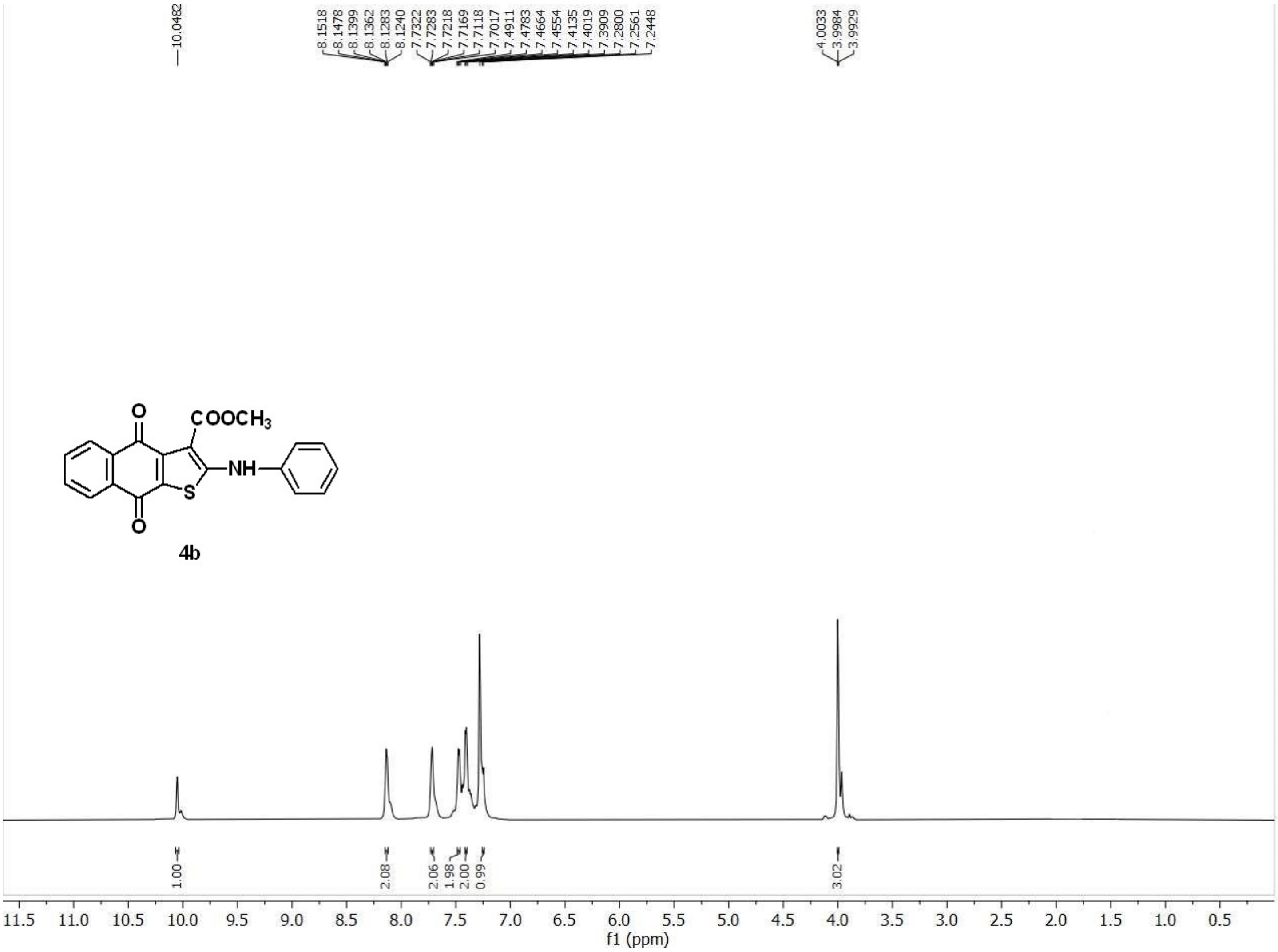

^{13}C NMR (151MHz, $CDCl_3$)

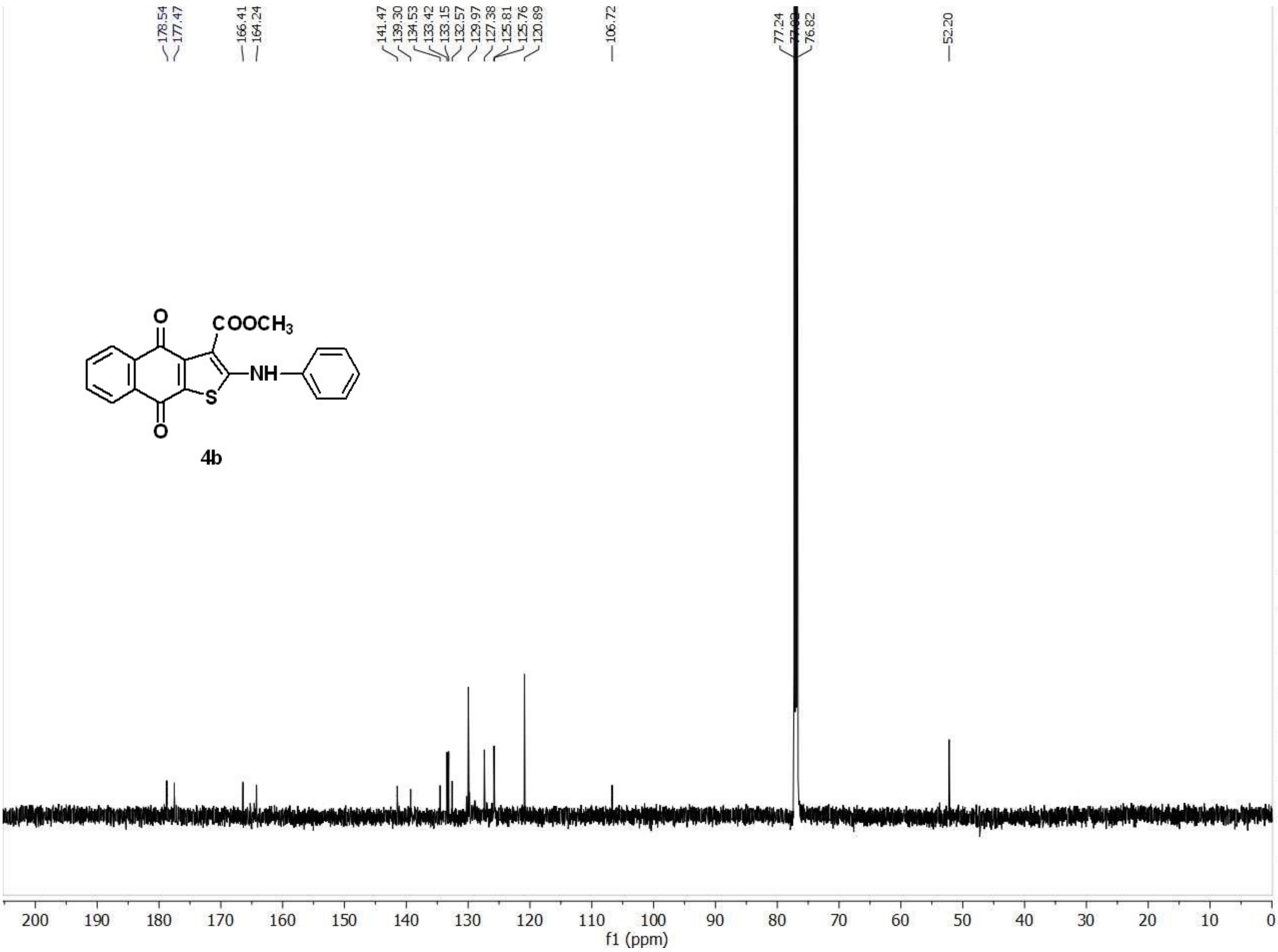

^{1}H NMR (400MHz, $CDCl_3$)

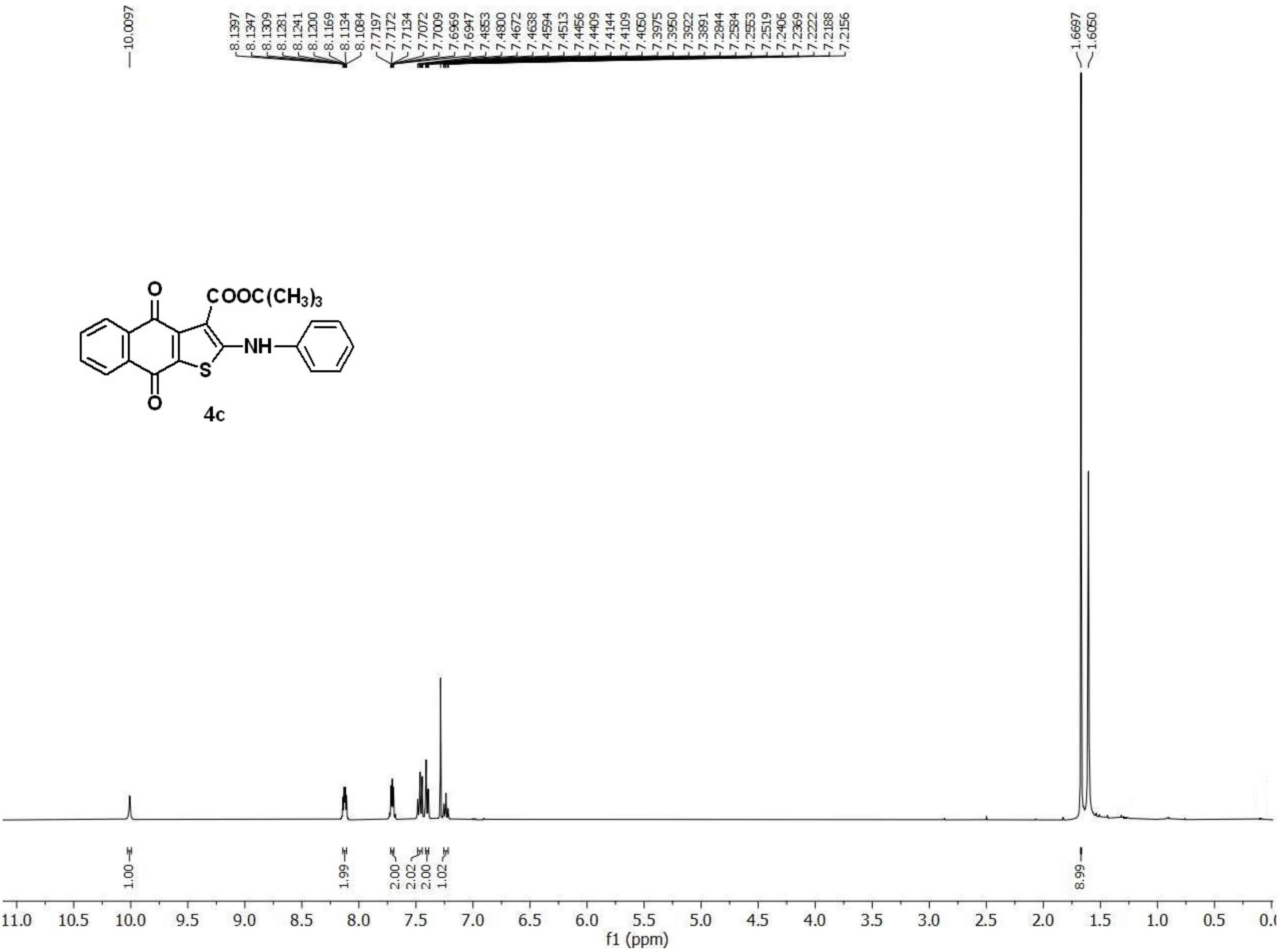

^{13}C NMR (151MHz, $CDCl_3$)

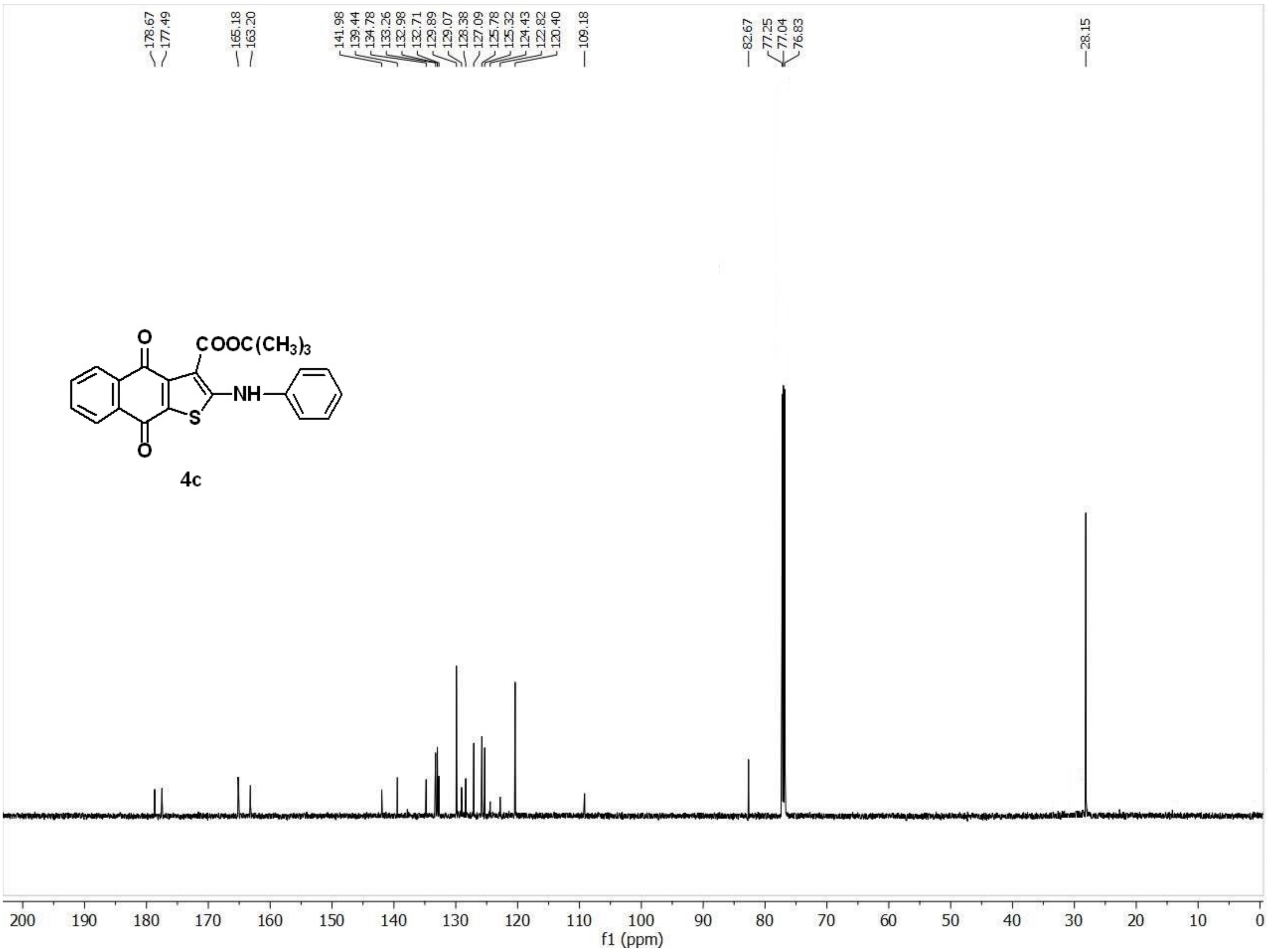

^{1}H NMR (400MHz, $CDCl_3$)

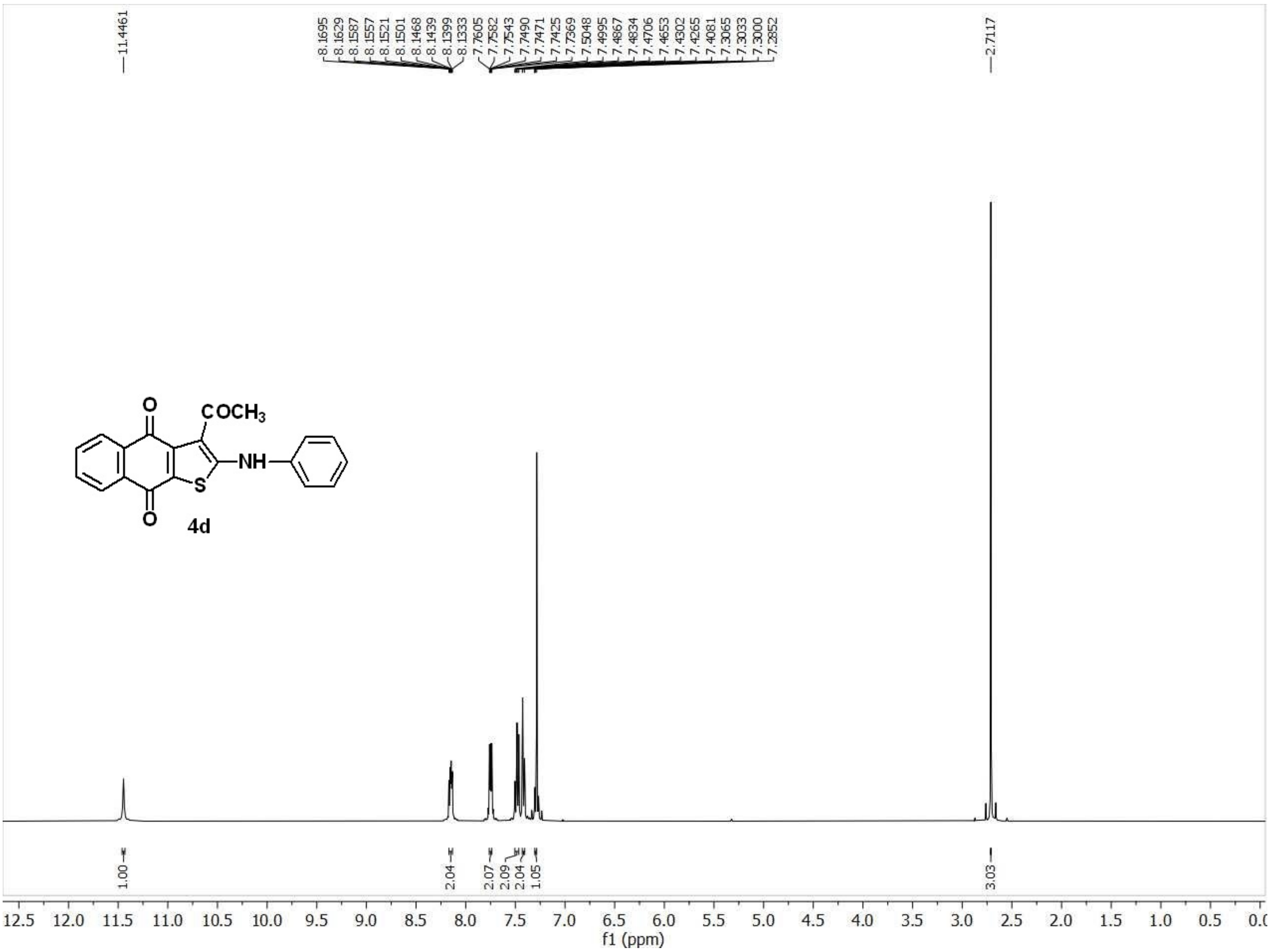

^{13}C NMR (101MHz, $CDCl_3$)

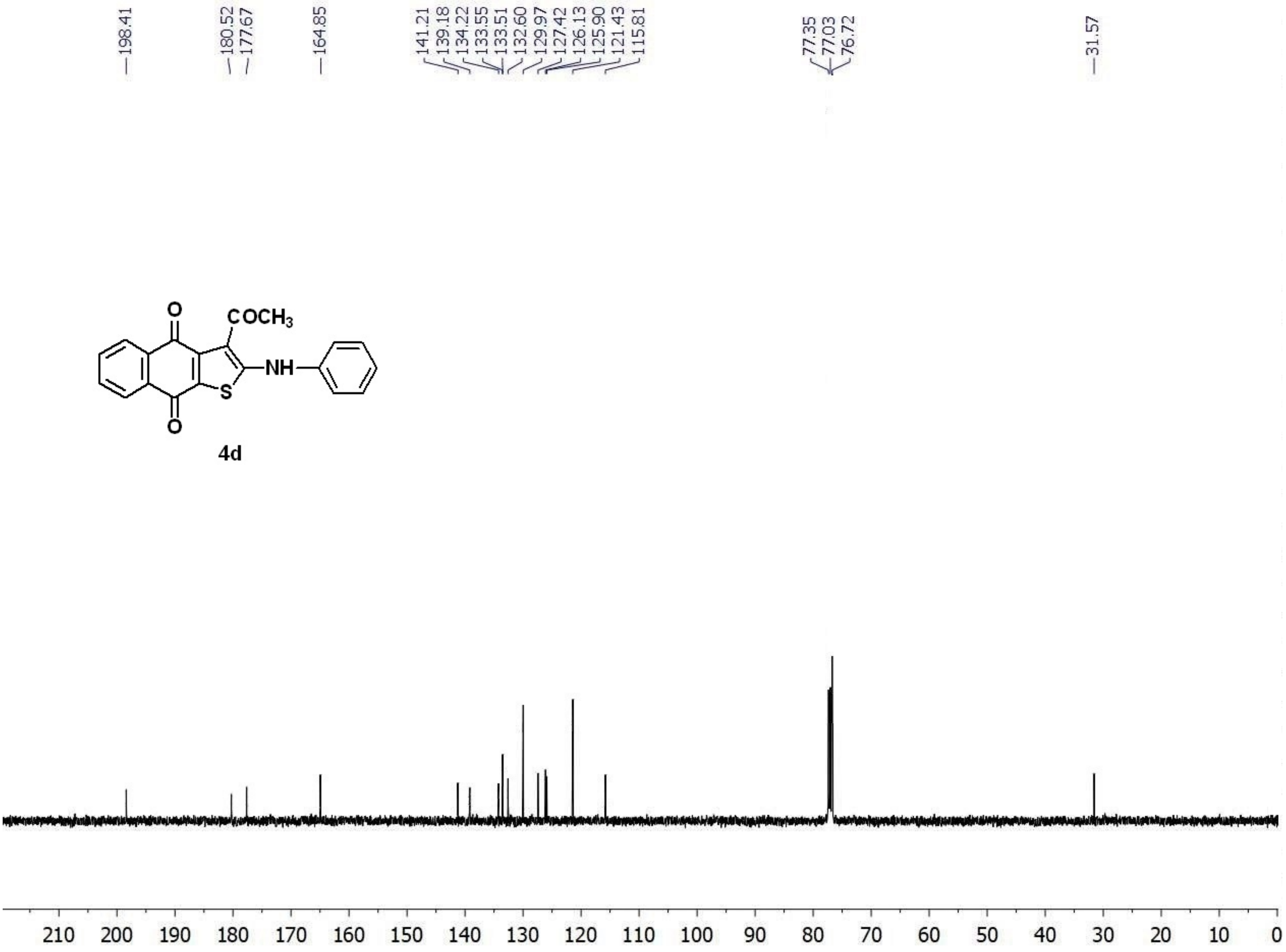

^{1}H NMR (400MHz, $CDCl_3$)

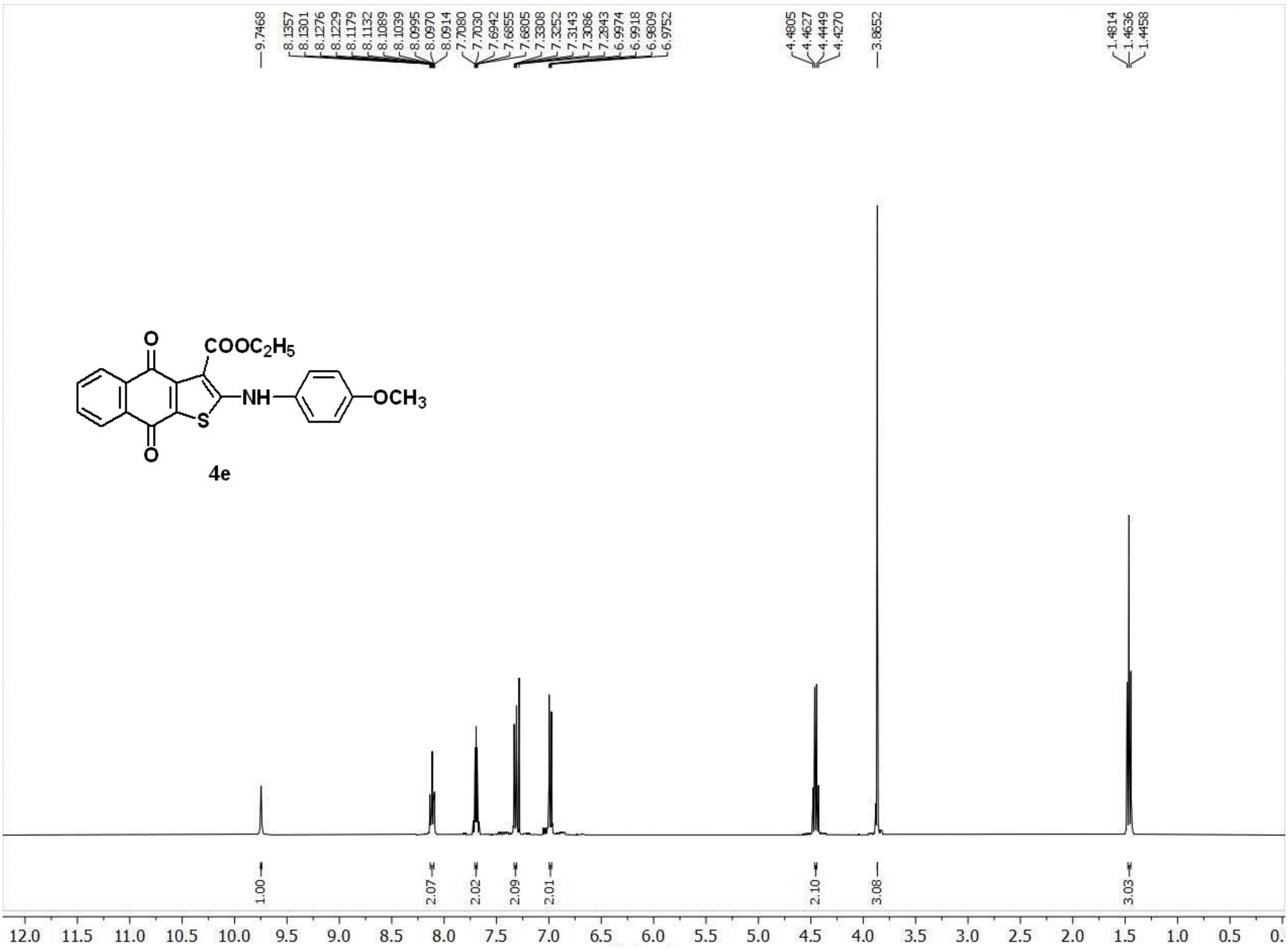

COOC2H5
NH
OCH3
S
O
4e
12.0 11.5 11.0 10.5 10.0 9.5 9.0 8.5 8.0 7.5 7.0 6.5 6.0 5.5 5.0 4.5 4.0 3.5 3.0 2.5 2.0 1.5 1.0 0.5

^{13}C NMR (101MHz, $CDCl_3$)

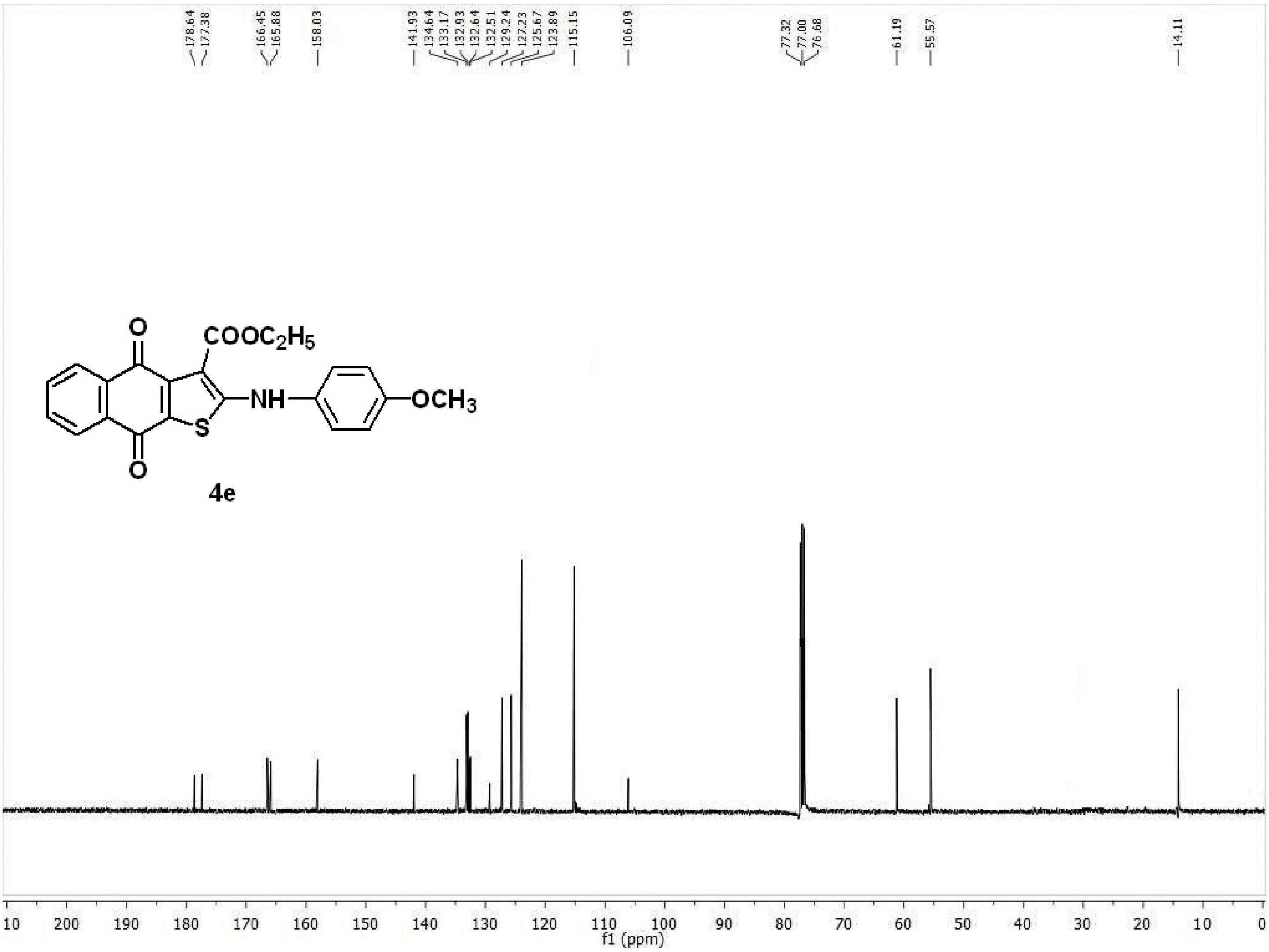

^{1}H NMR (400MHz, $CDCl_3$)

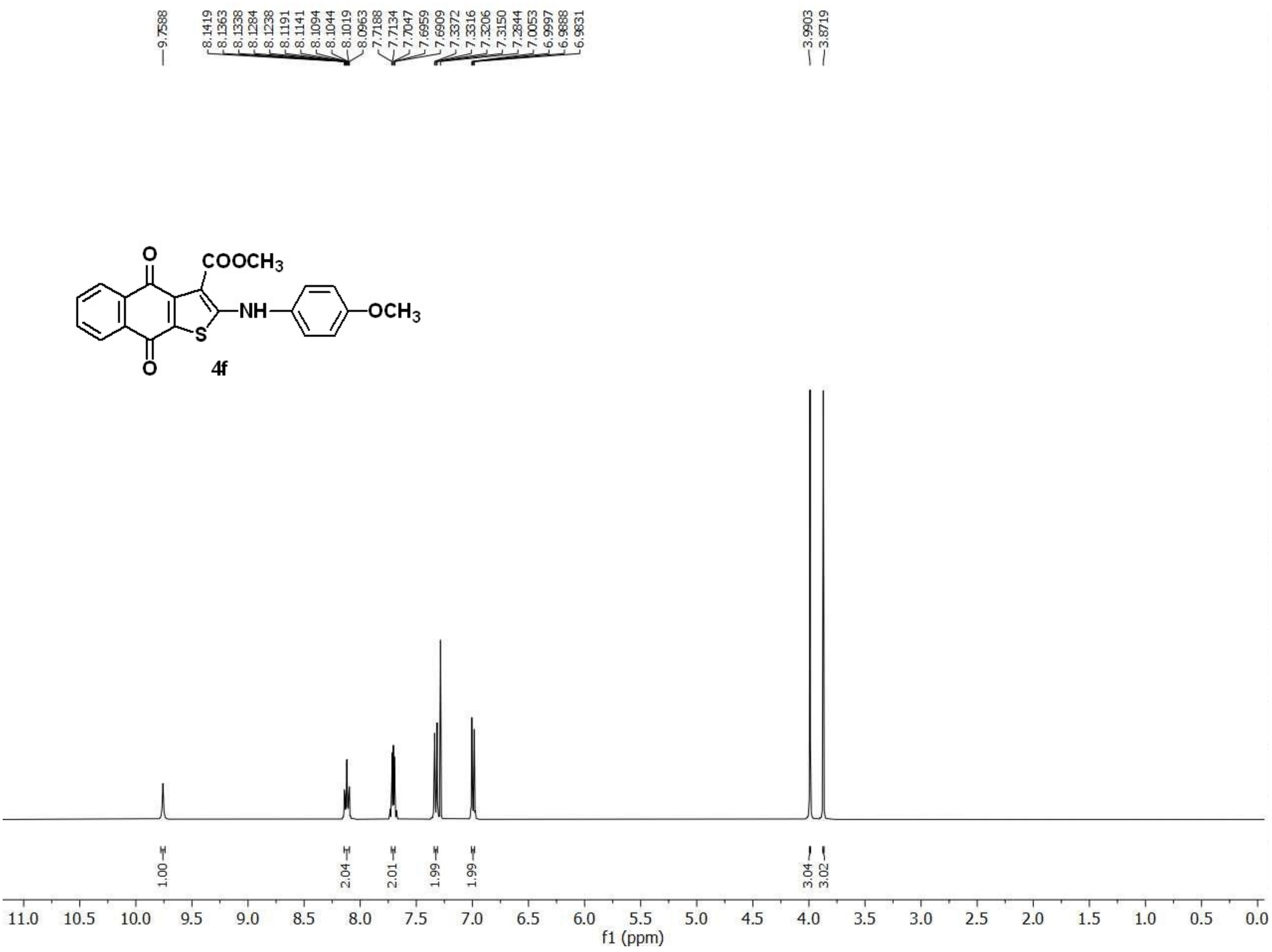

^{13}C NMR (151MHz, $CDCl_3$)

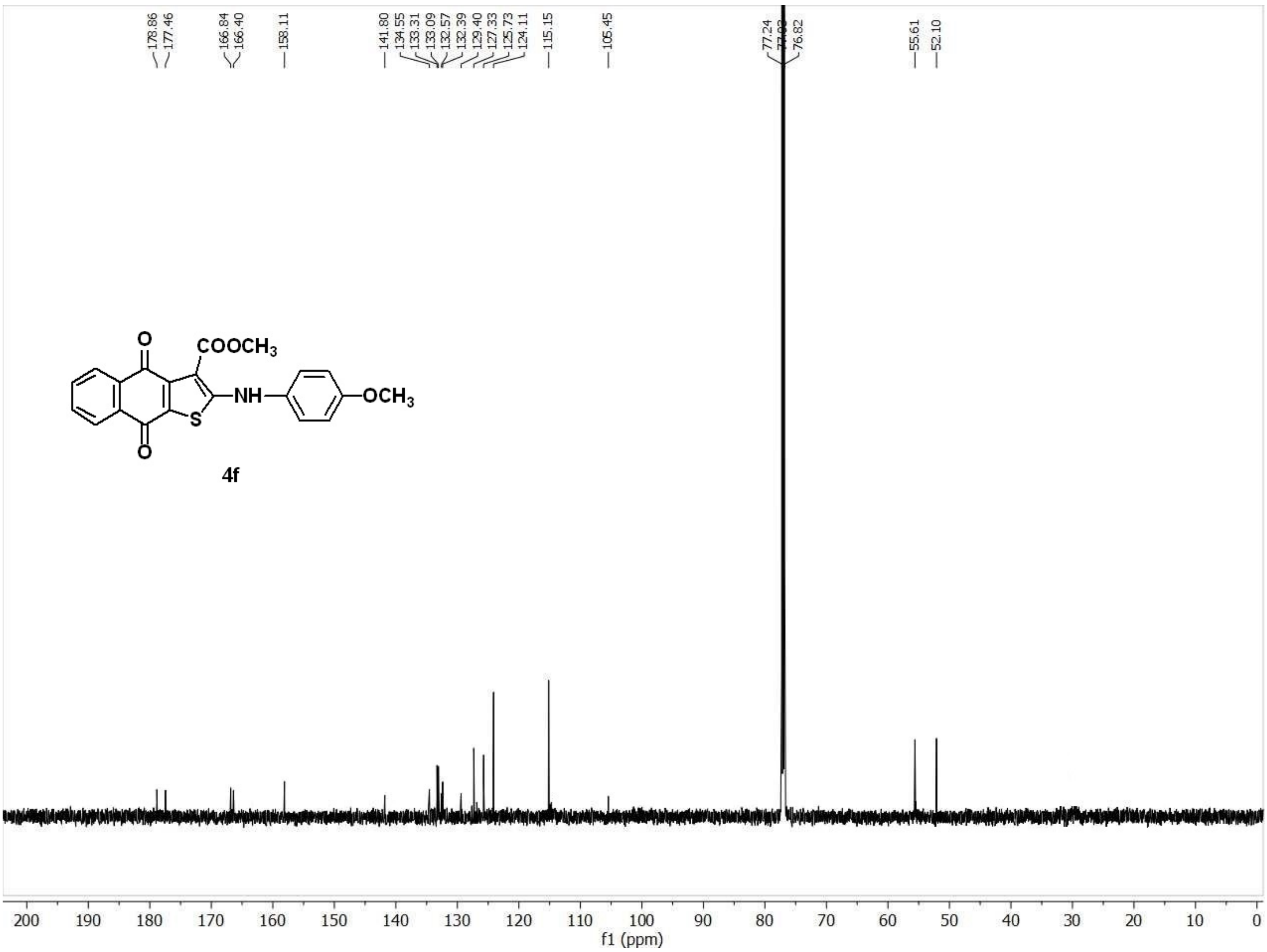

^{1}H NMR (400MHz, $CDCl_3$)

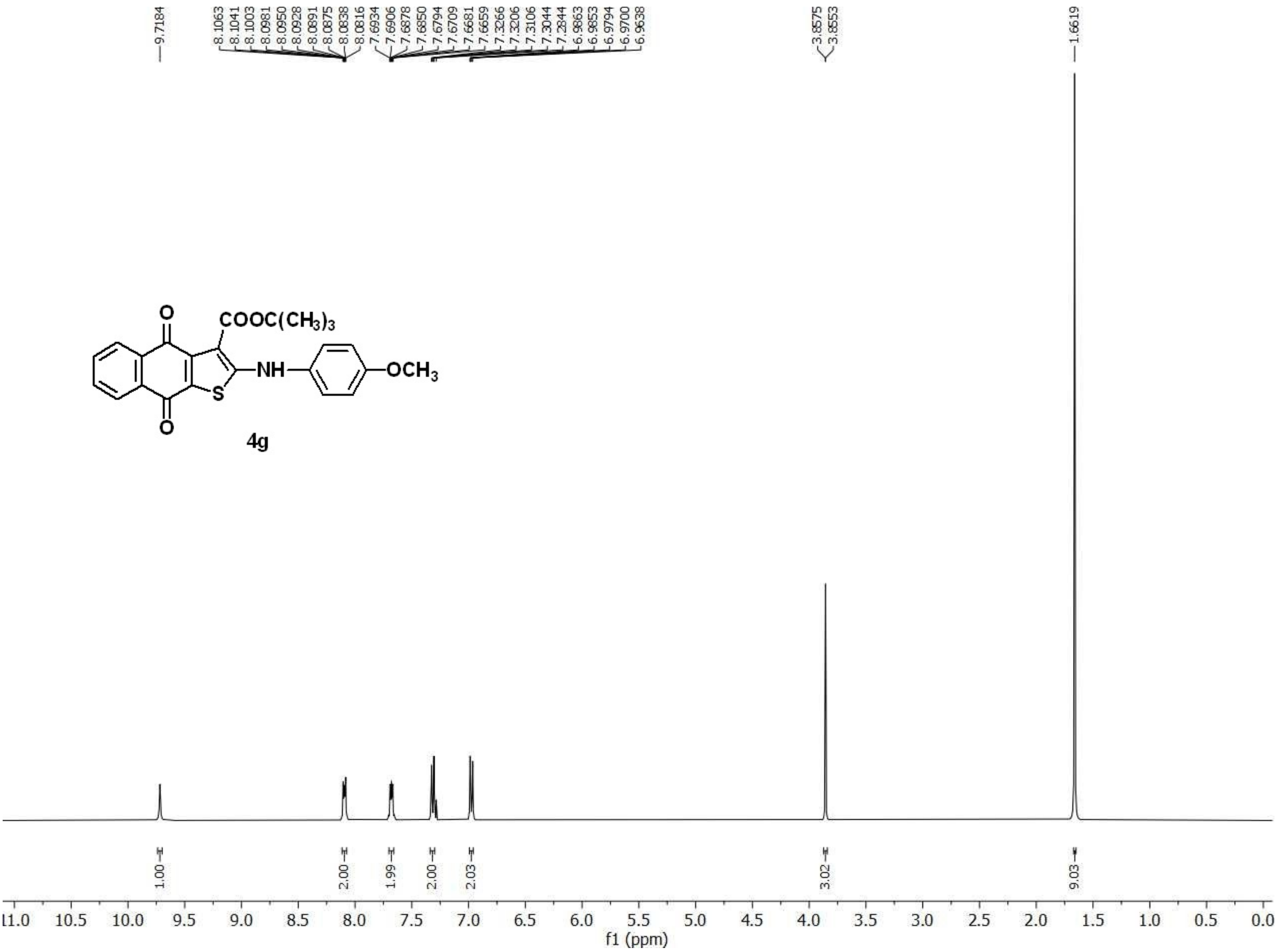

^{13}C NMR (151MHz, $CDCl_3$)

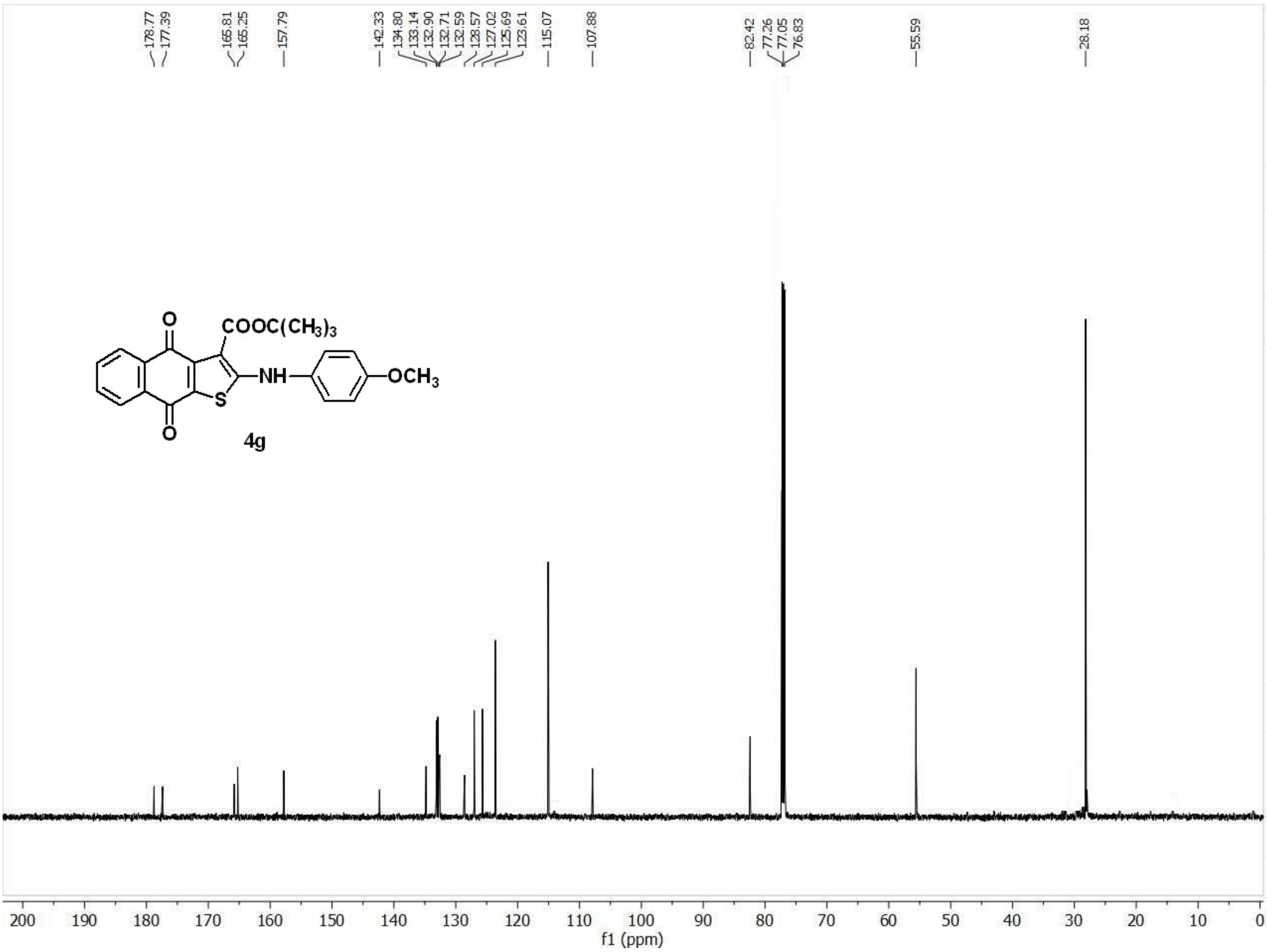

^{1}H NMR (300MHz, $CDCl_3$)

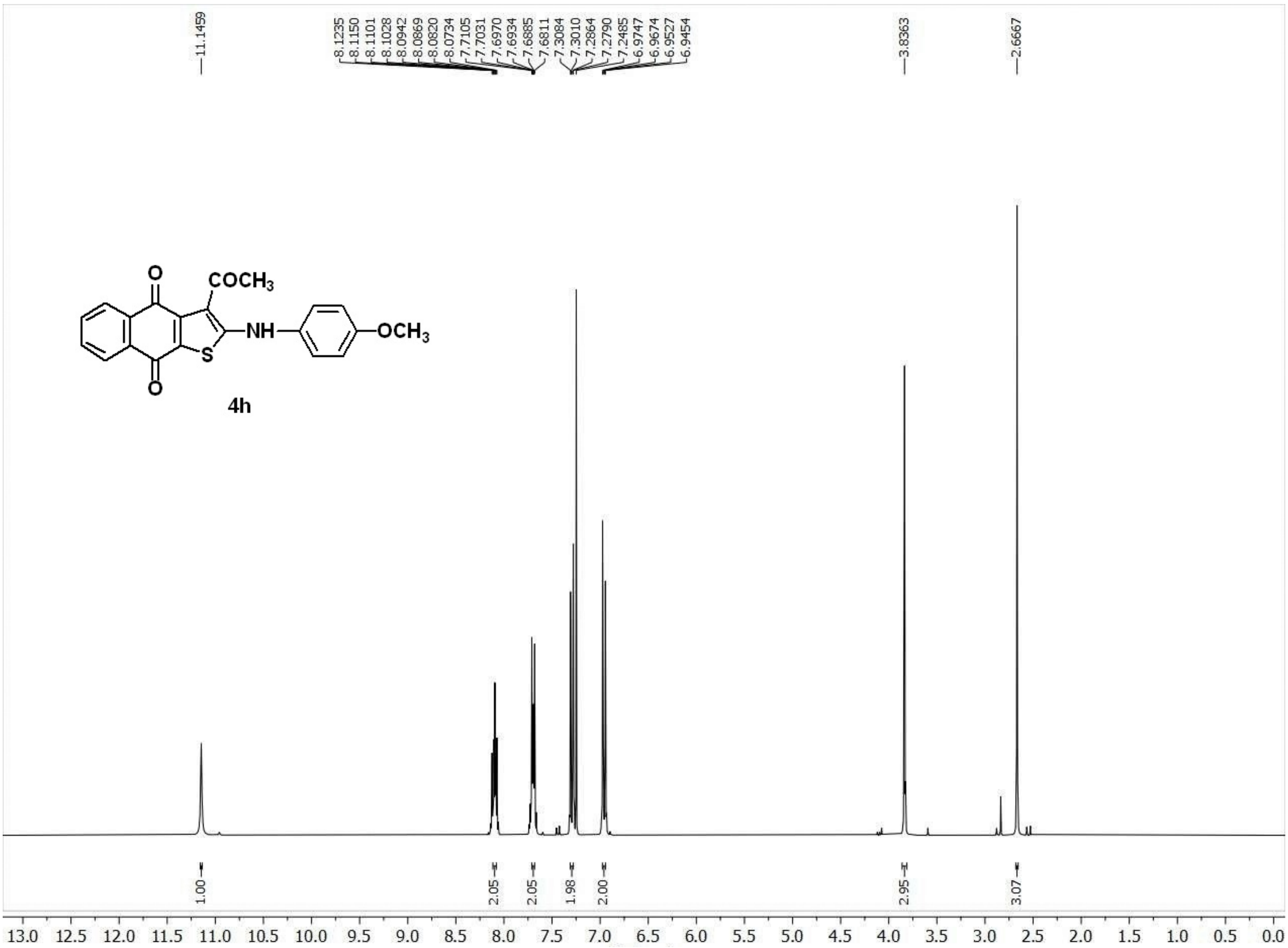

^{13}C NMR (75MHz, $CDCl_3$)

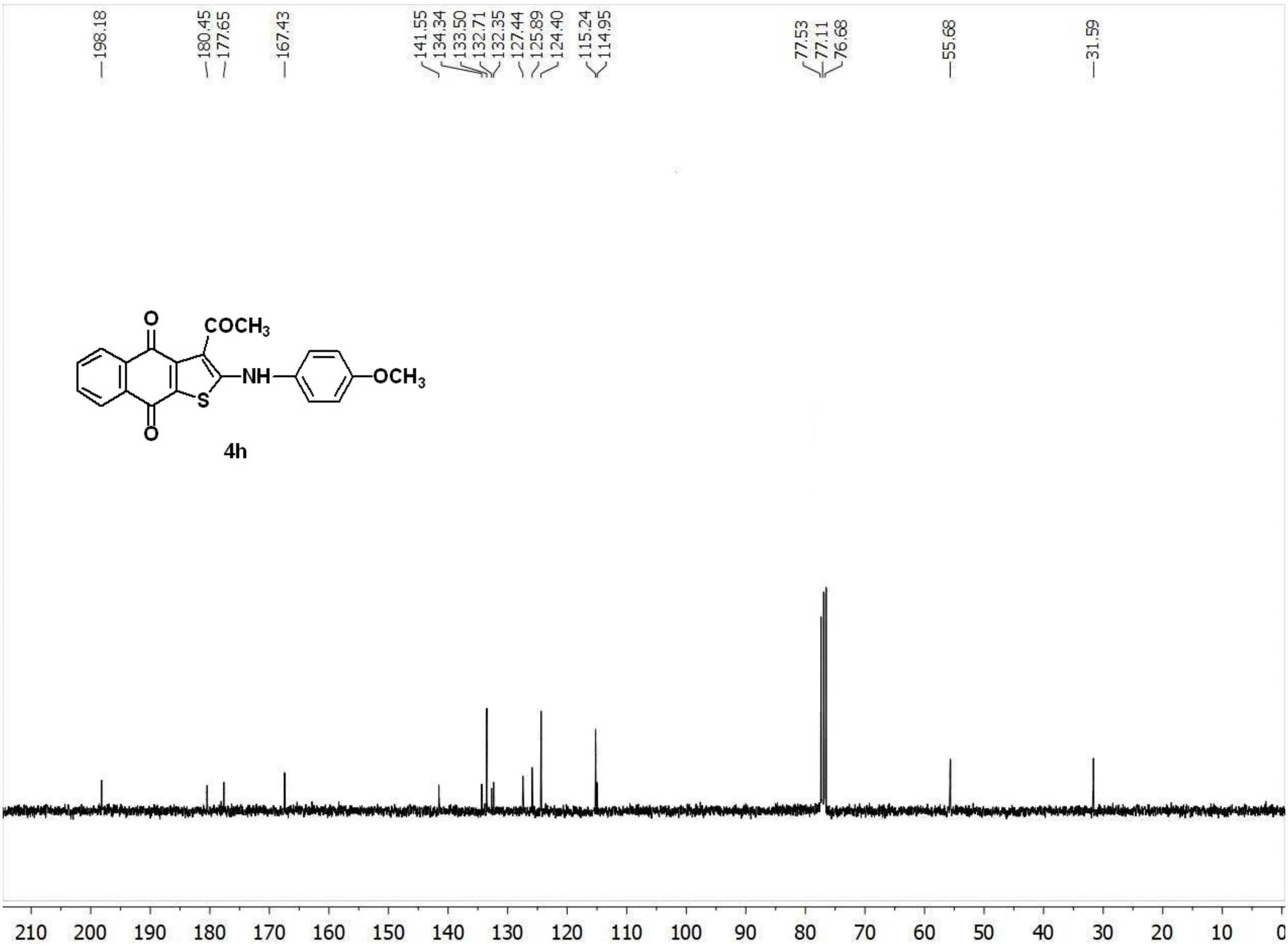

^{1}H NMR (300MHz, $CDCl_3$)

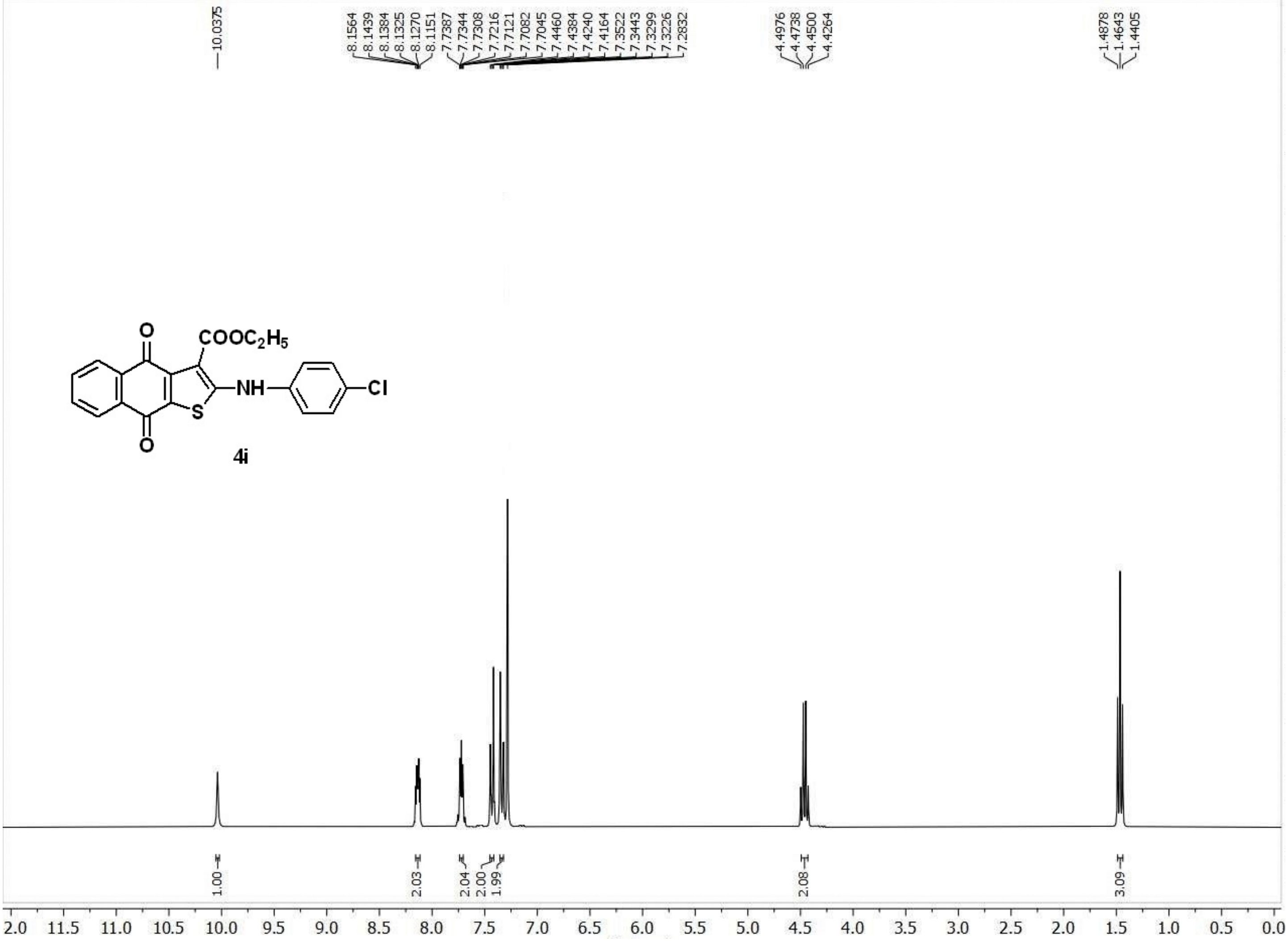

^{13}C NMR (75MHz, $CDCl_3$)

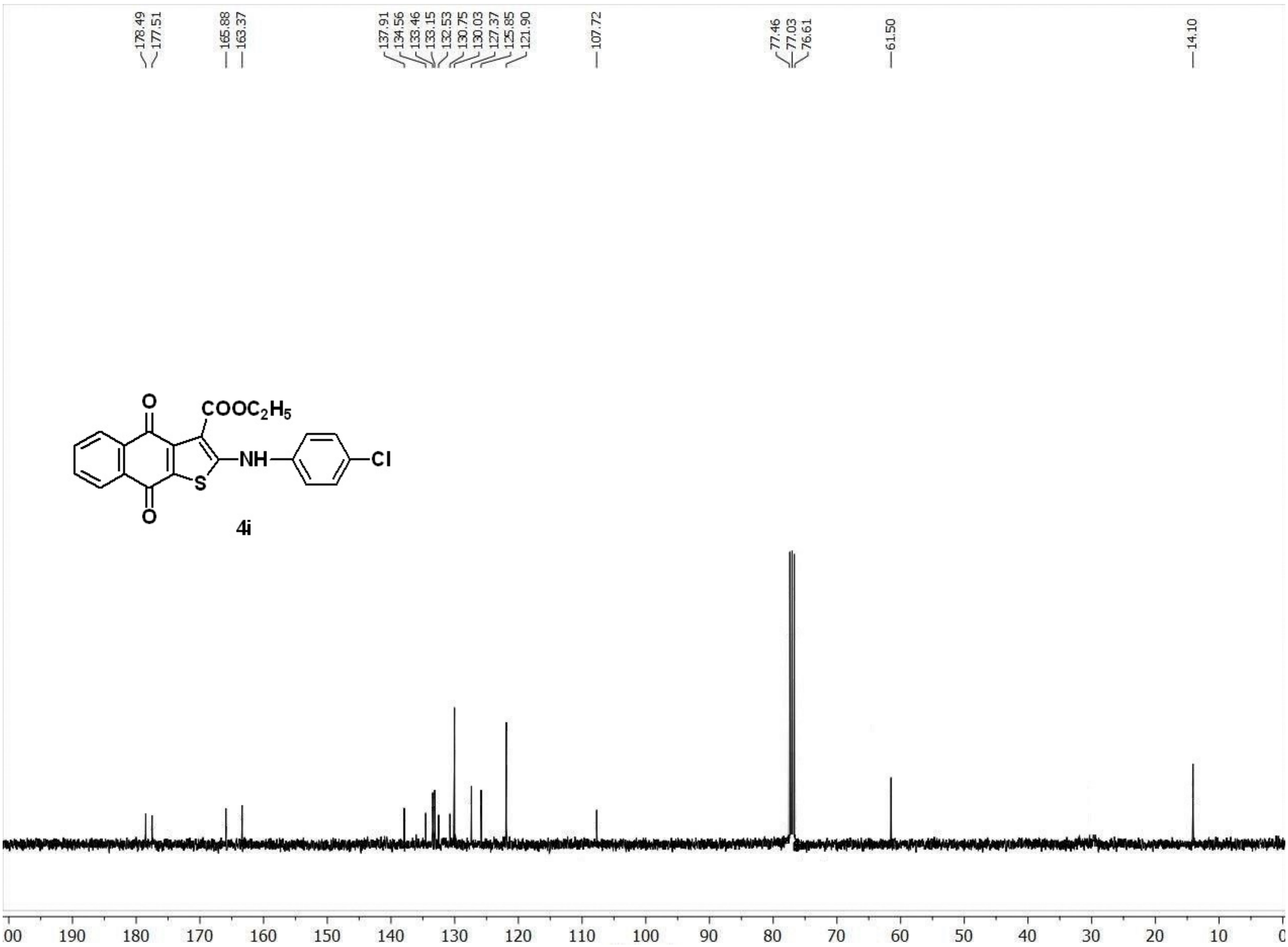

^1H NMR (400MHz, $CDCl_3$)

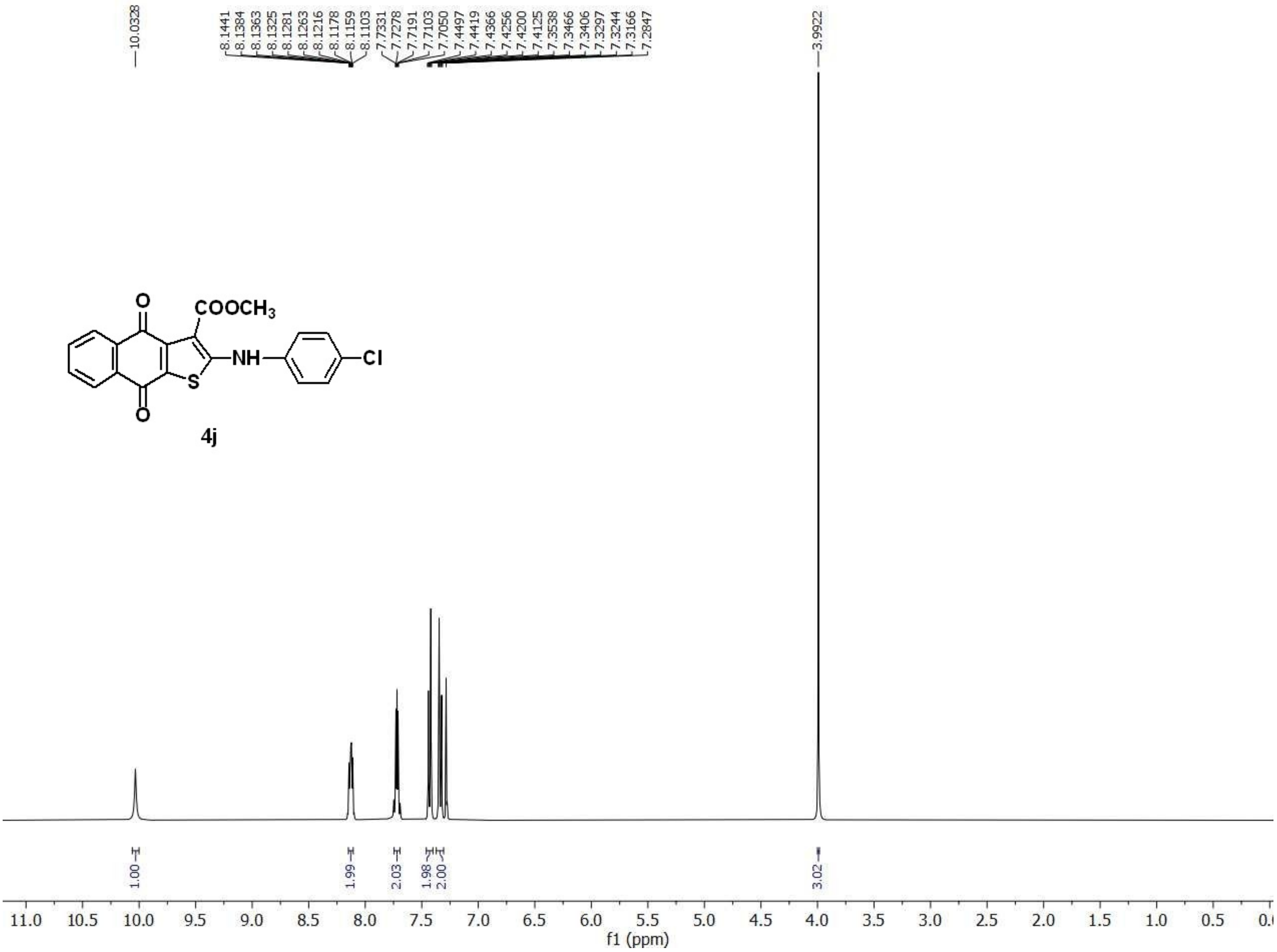

4j

^{13}C NMR (151MHz, $CDCl_3$)

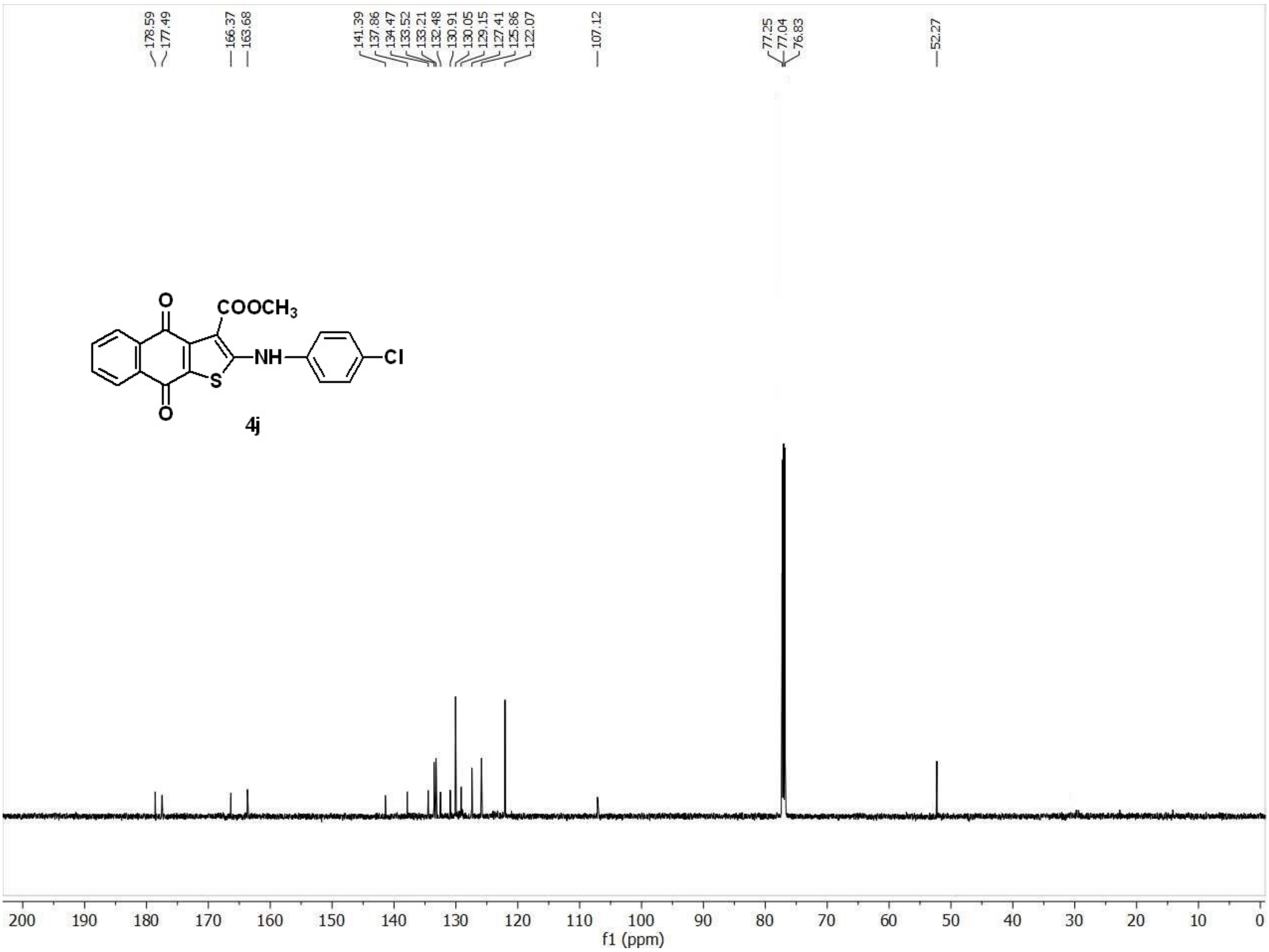

^{1}H NMR (600MHz, $CDCl_3$)

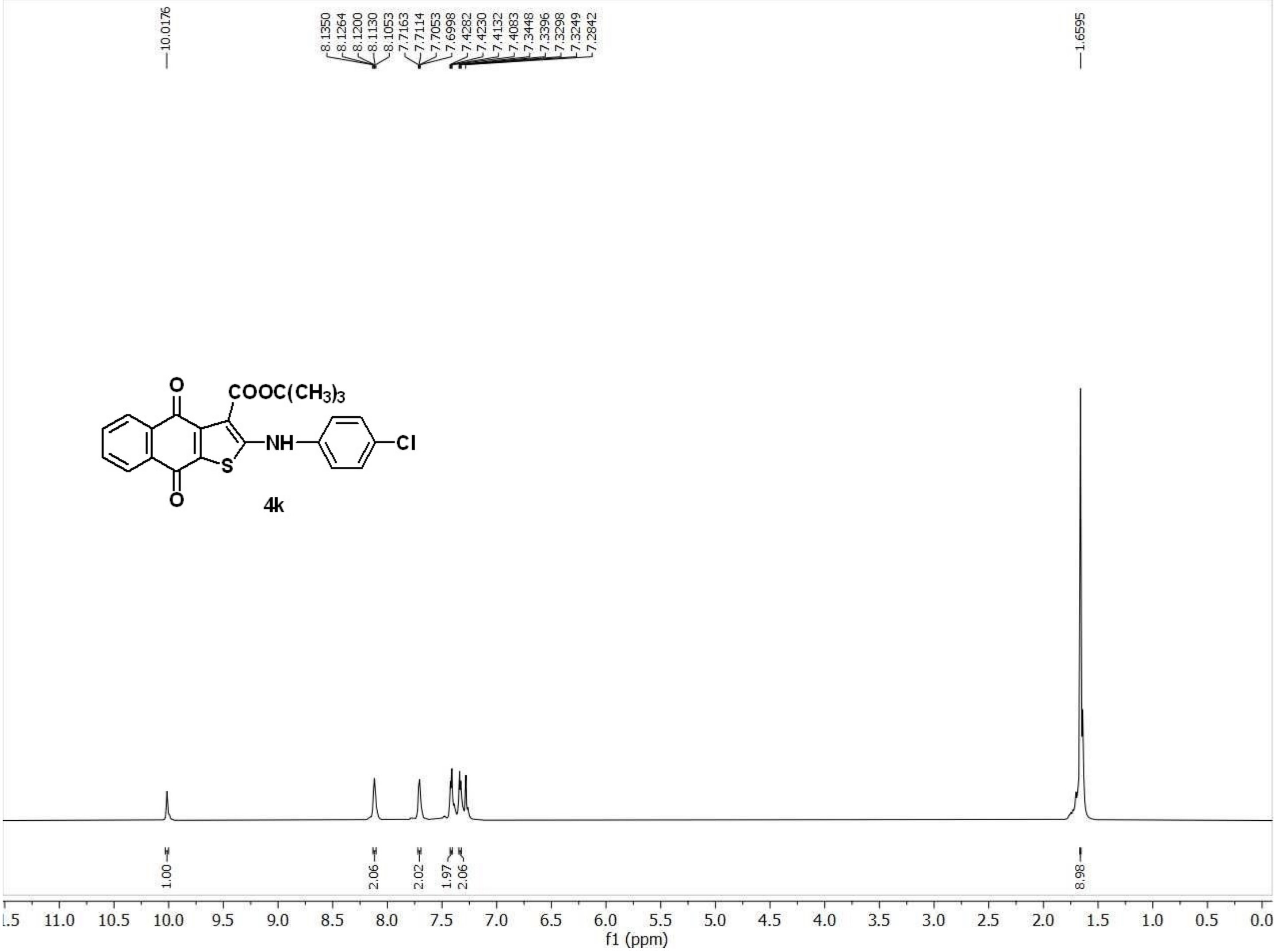

^{13}C NMR (151MHz, $CDCl_3$)

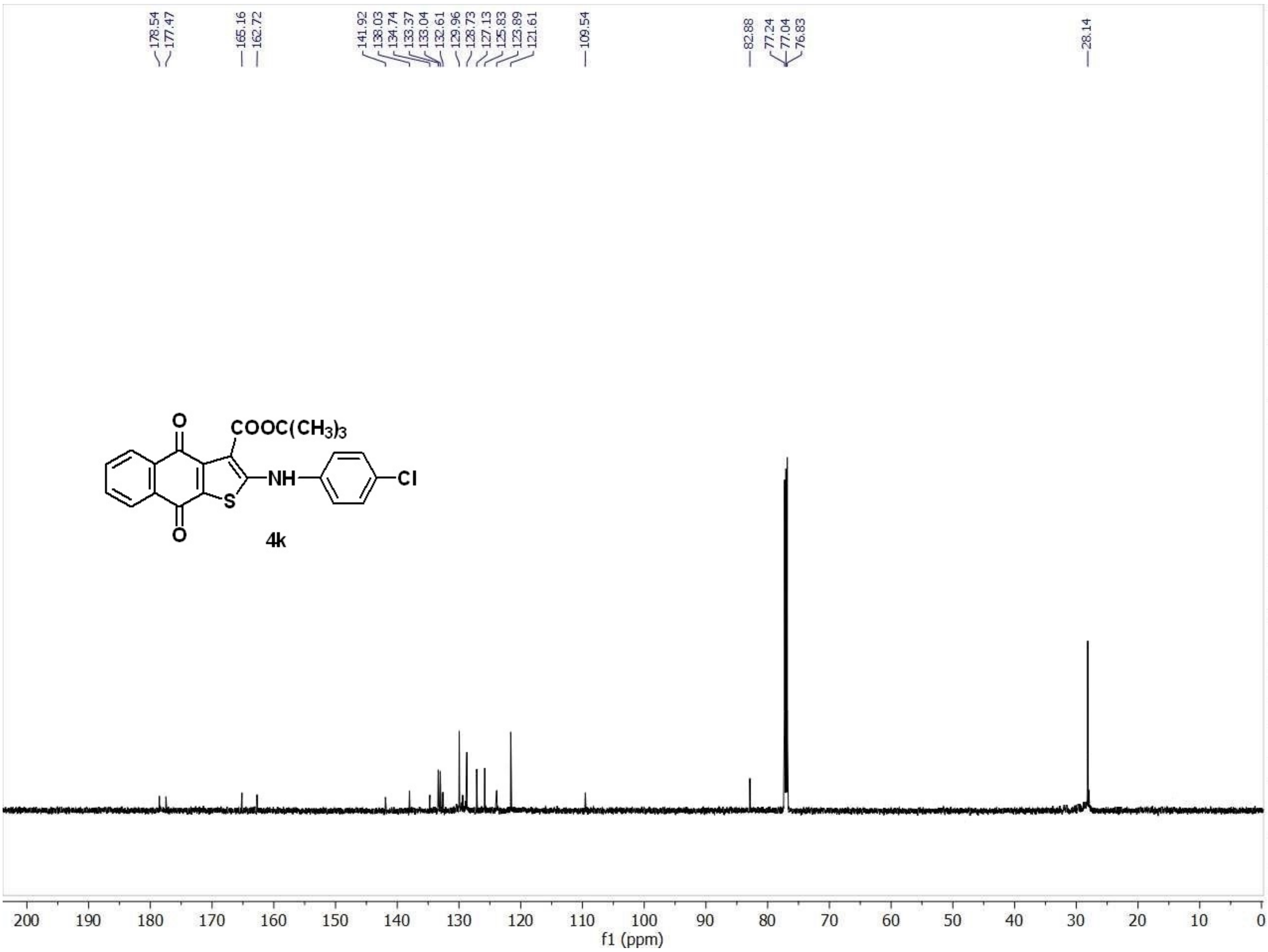

^{1}H NMR (300MHz, $CDCl_3$)

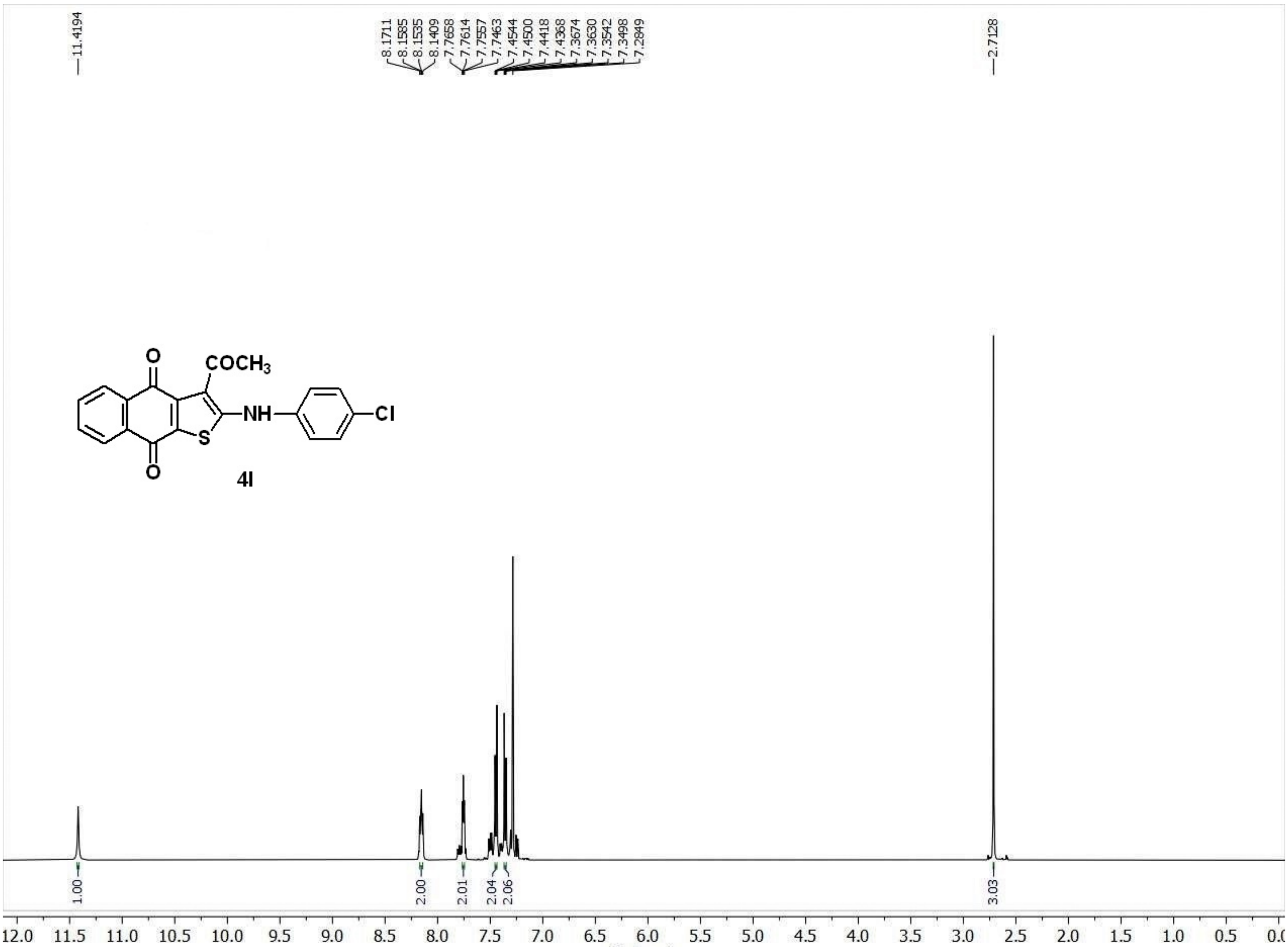

^{13}C NMR (75MHz, $CDCl_3$)

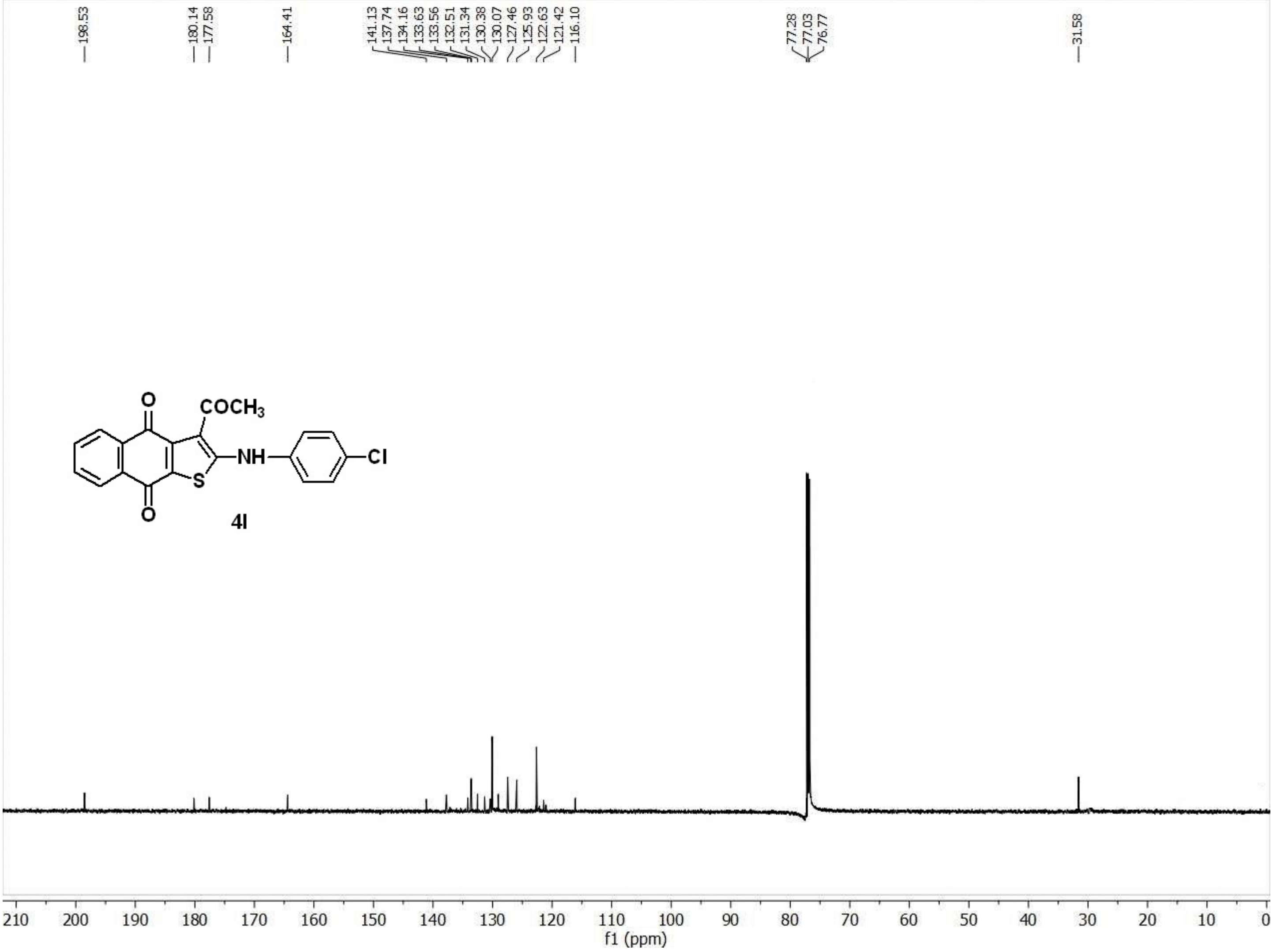

^{1}H NMR (400MHz, $CDCl_3$)

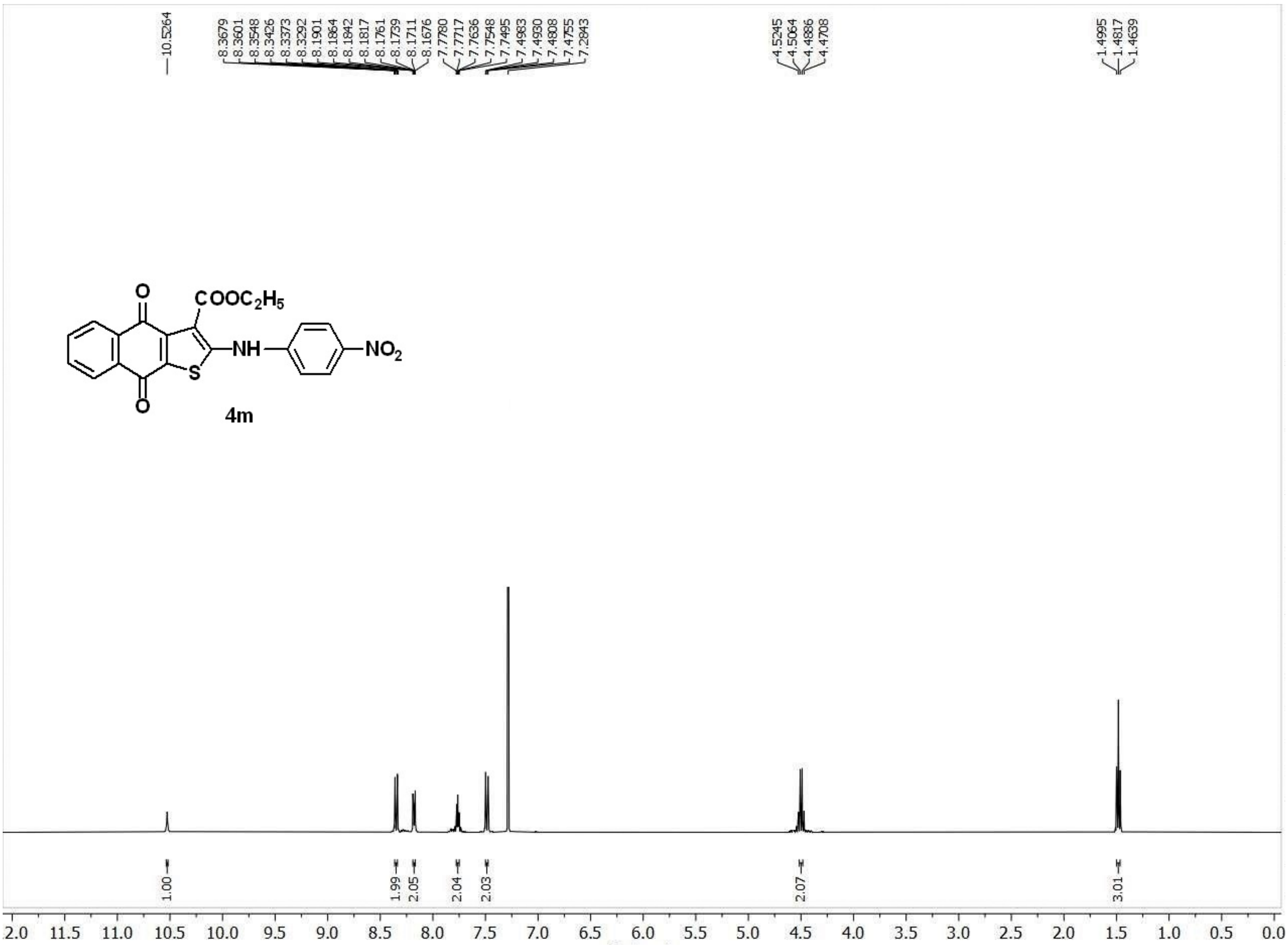

^{13}C NMR (101MHz, $CDCl_3$)

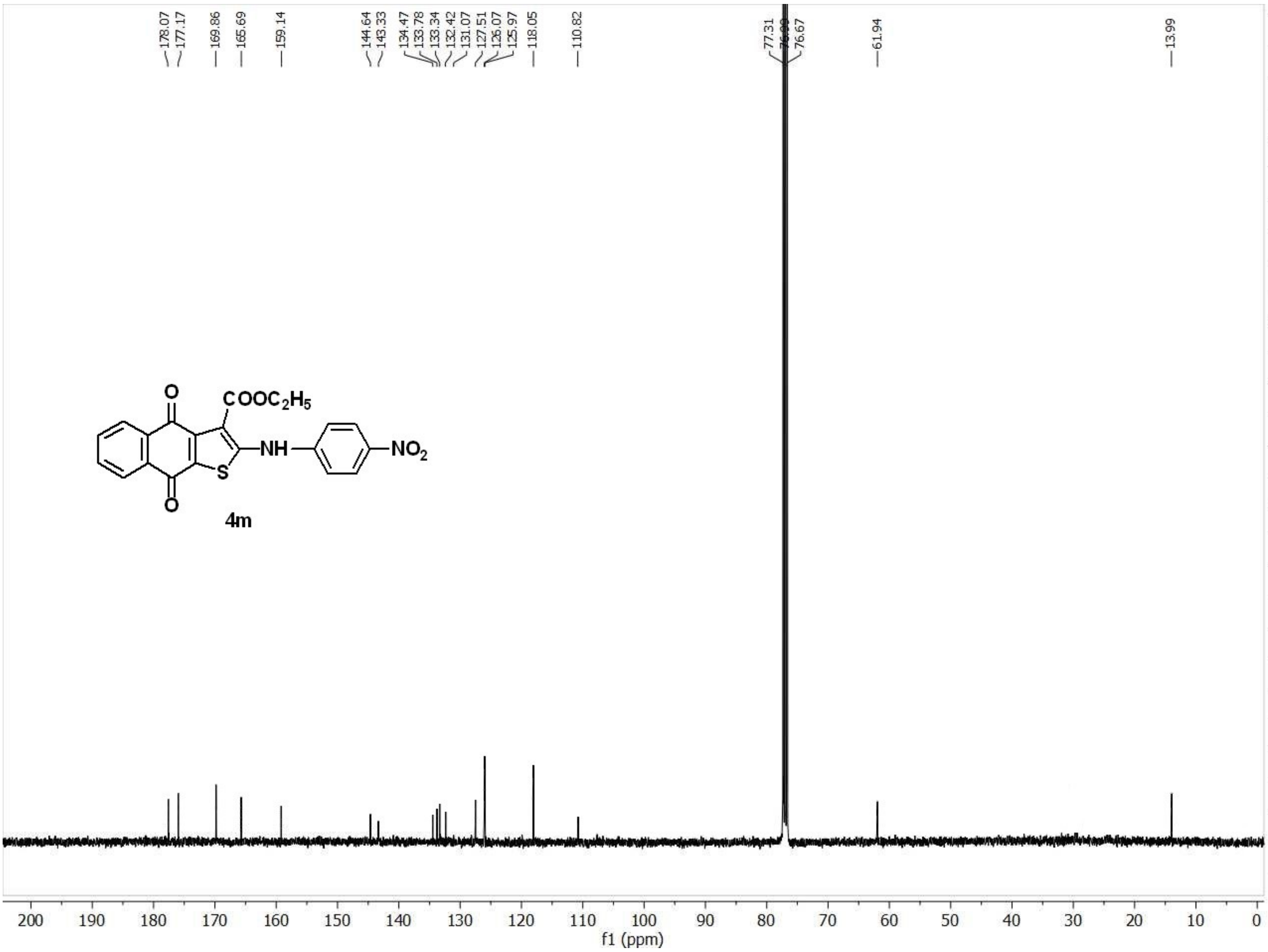

^{1}H NMR (500MHz, $CDCl_3$)

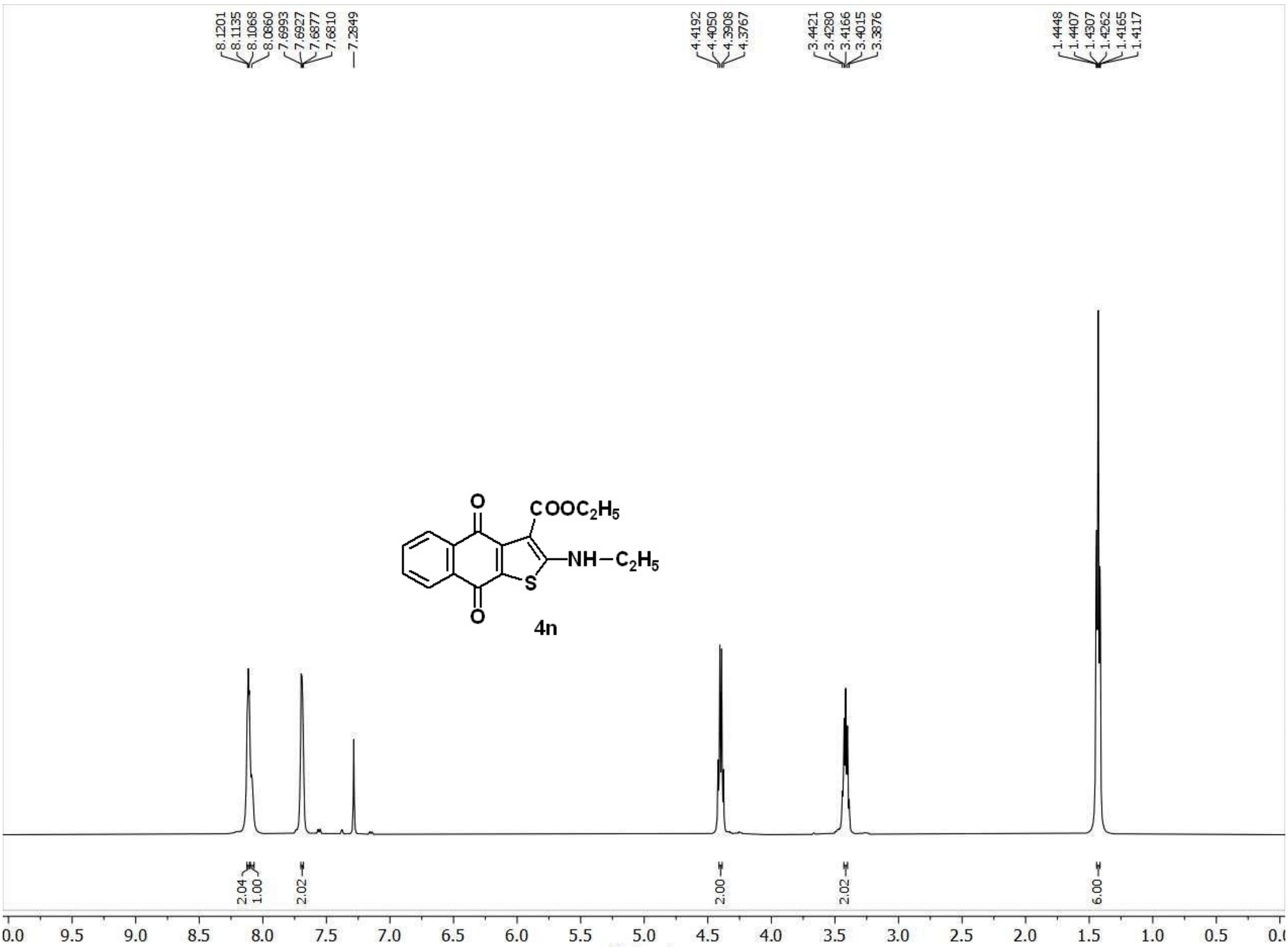

^{13}C NMR (126MHz, $CDCl_3$)

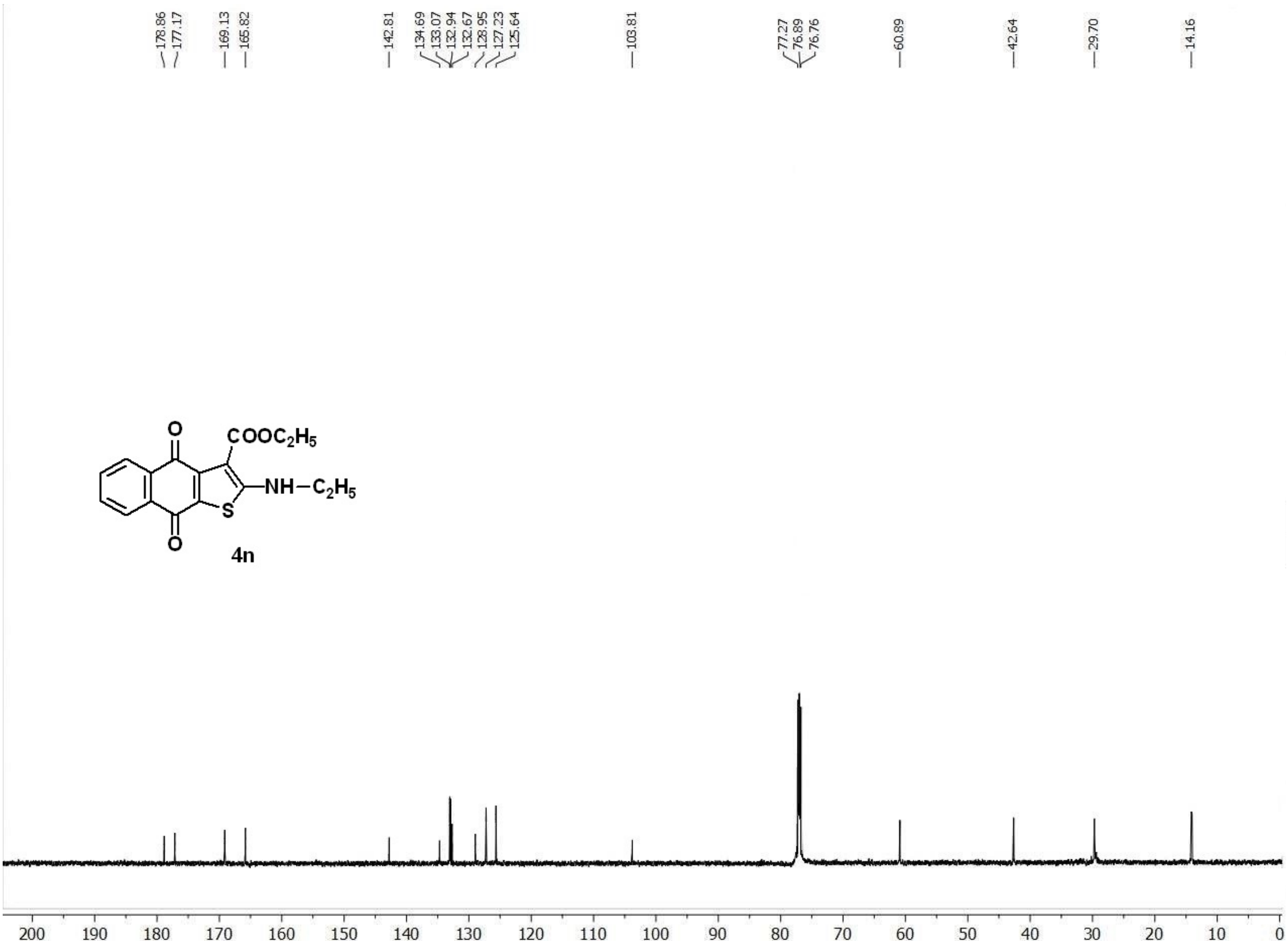

^{1}H NMR (400MHz, $CDCl_3$)

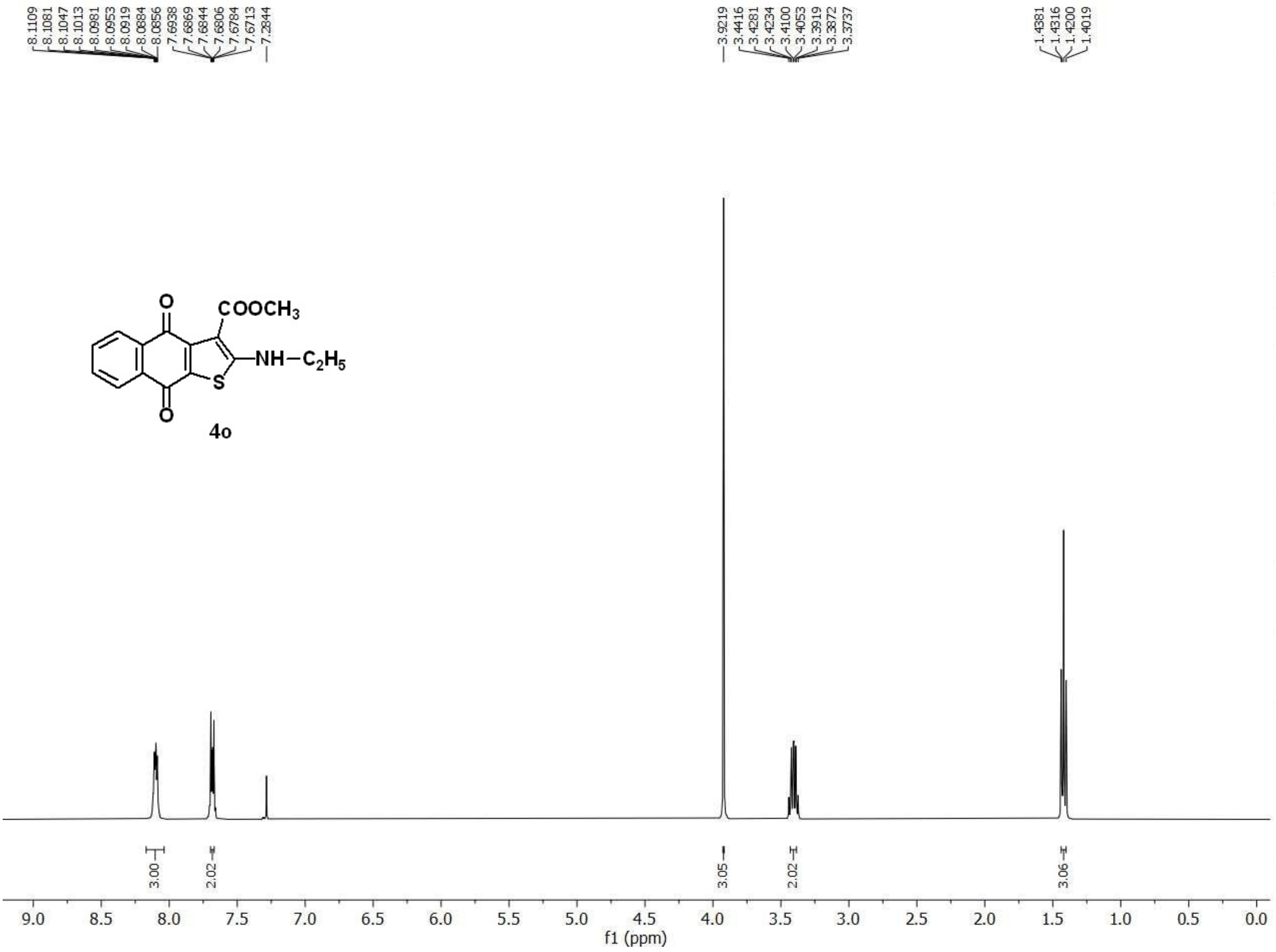

^{13}C NMR (151MHz, $CDCl_3$)

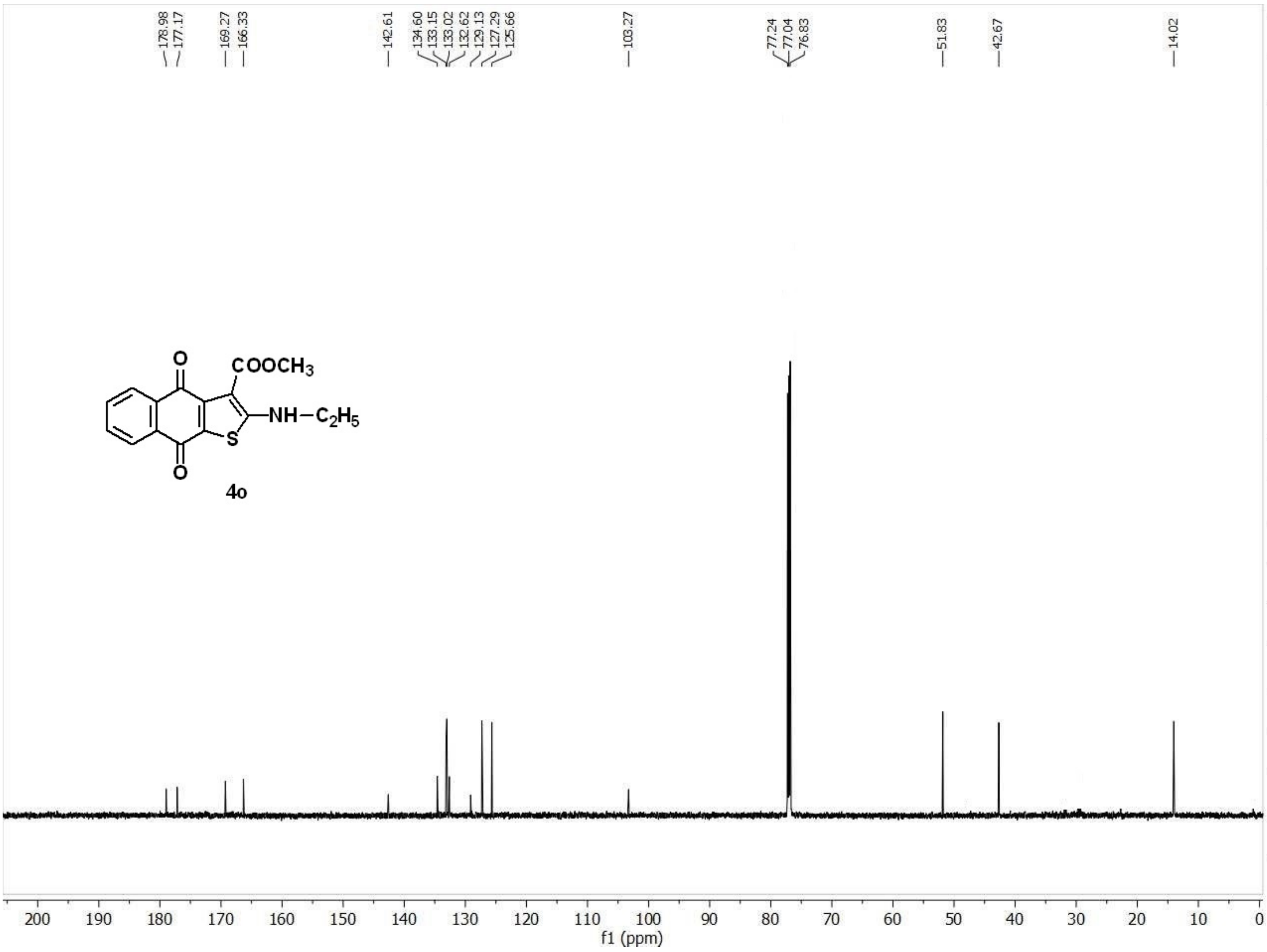

^{1}H NMR (600MHz, $CDCl_3$)

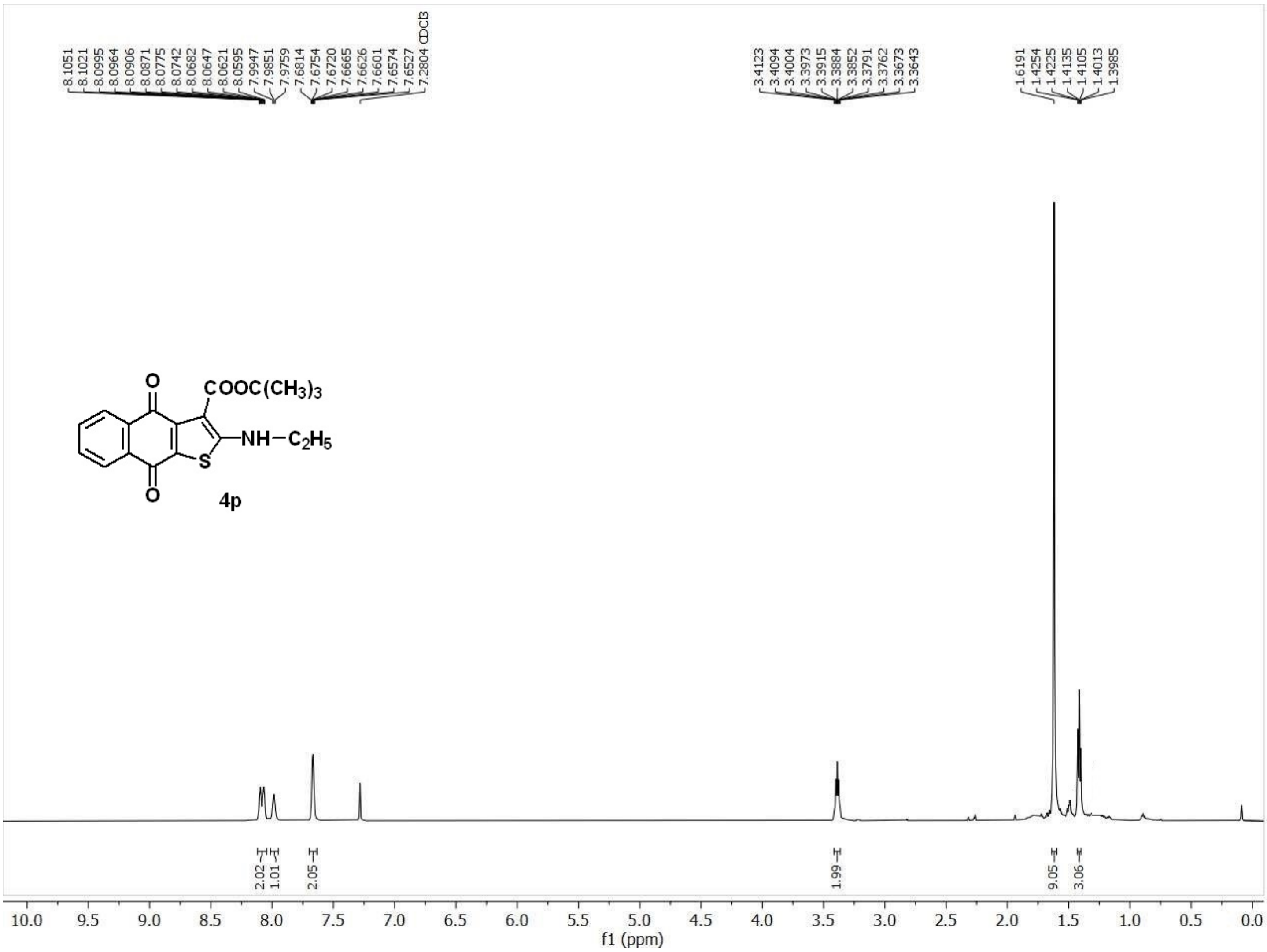

^{13}C NMR (151MHz, $CDCl_3$)

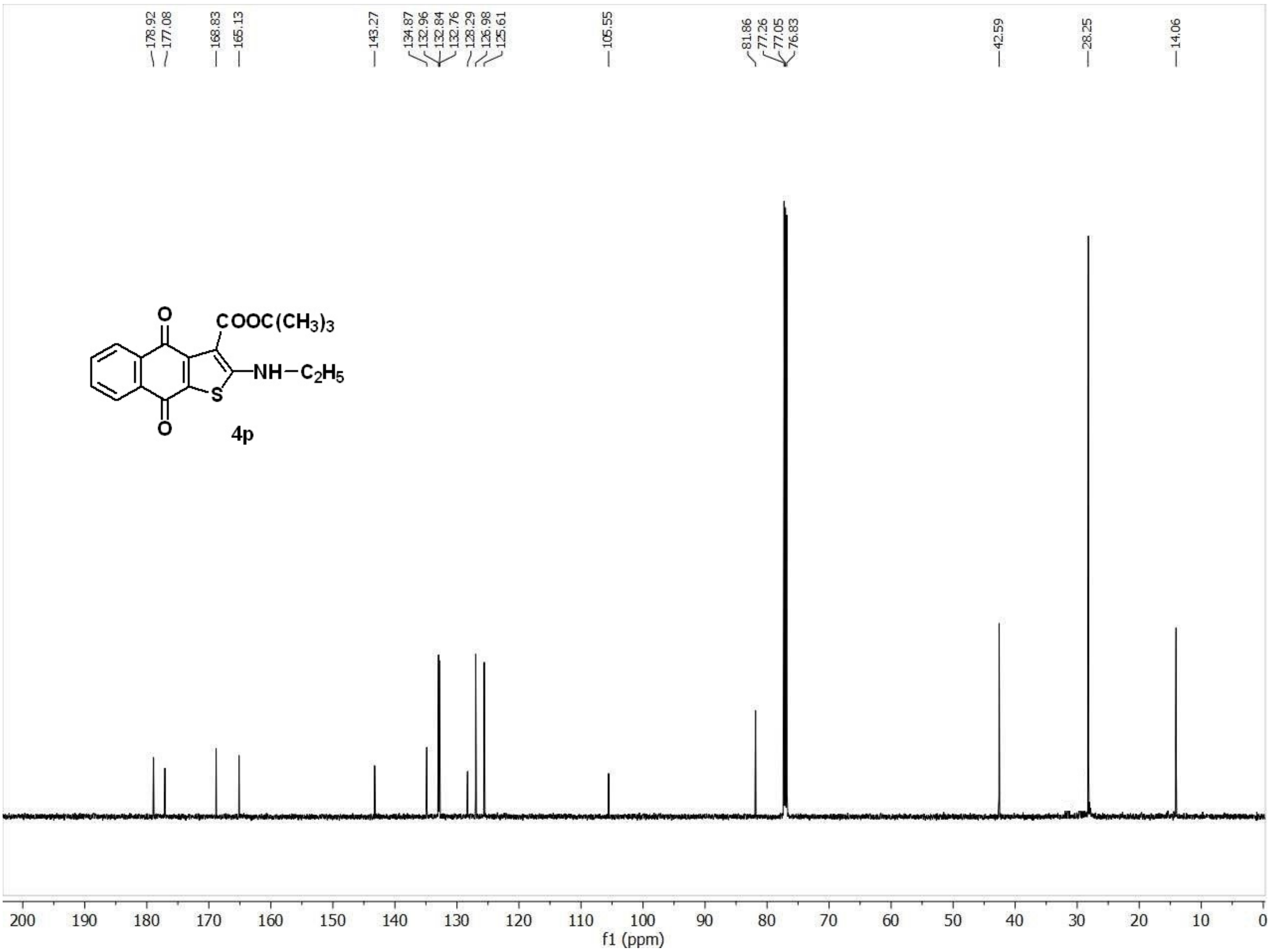

^{1}H NMR (500MHz, $CDCl_3$)

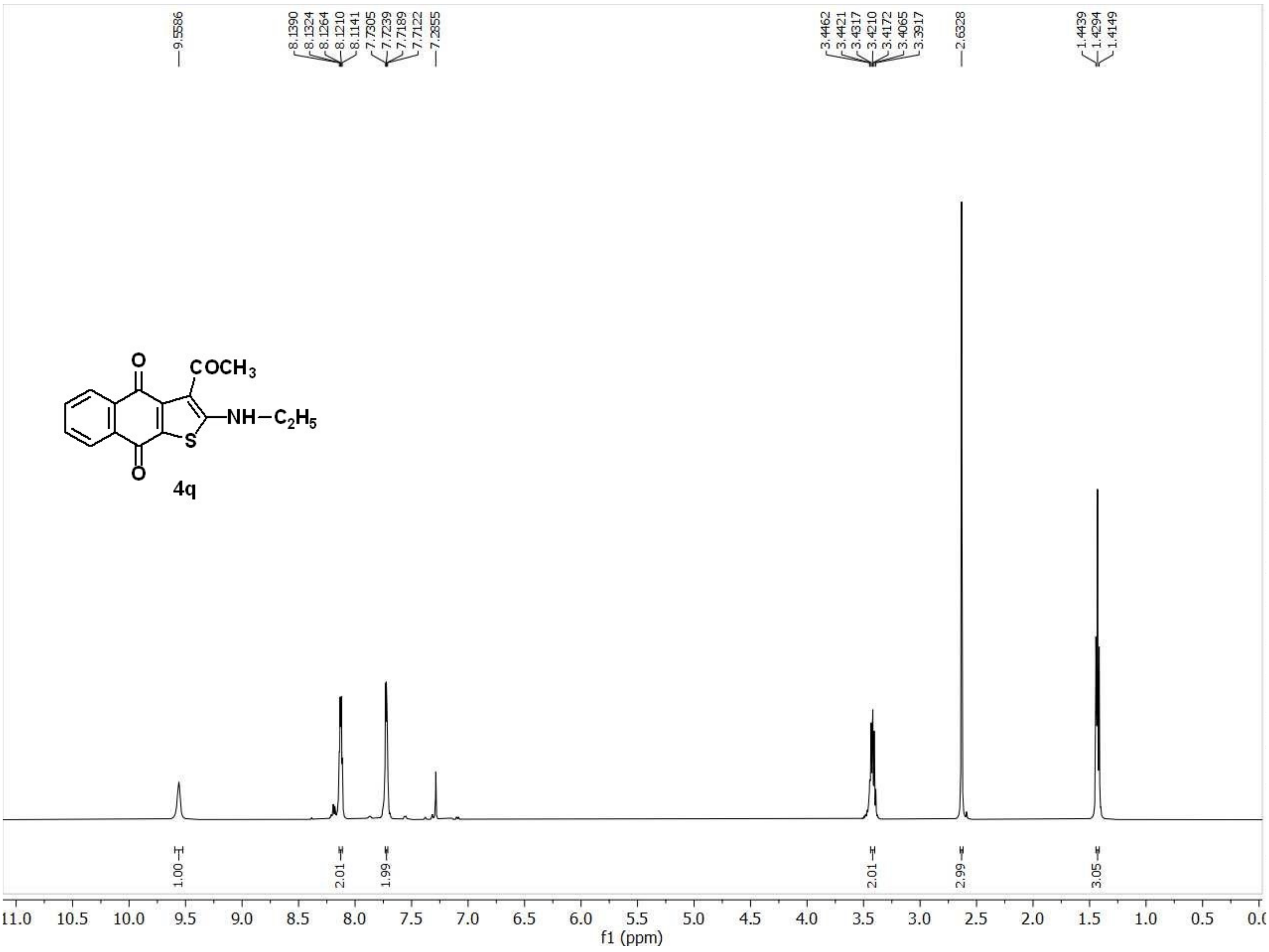

^{13}C NMR (126MHz, $CDCl_3$)

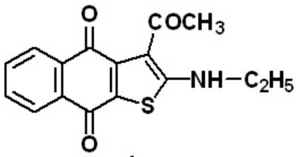

4q

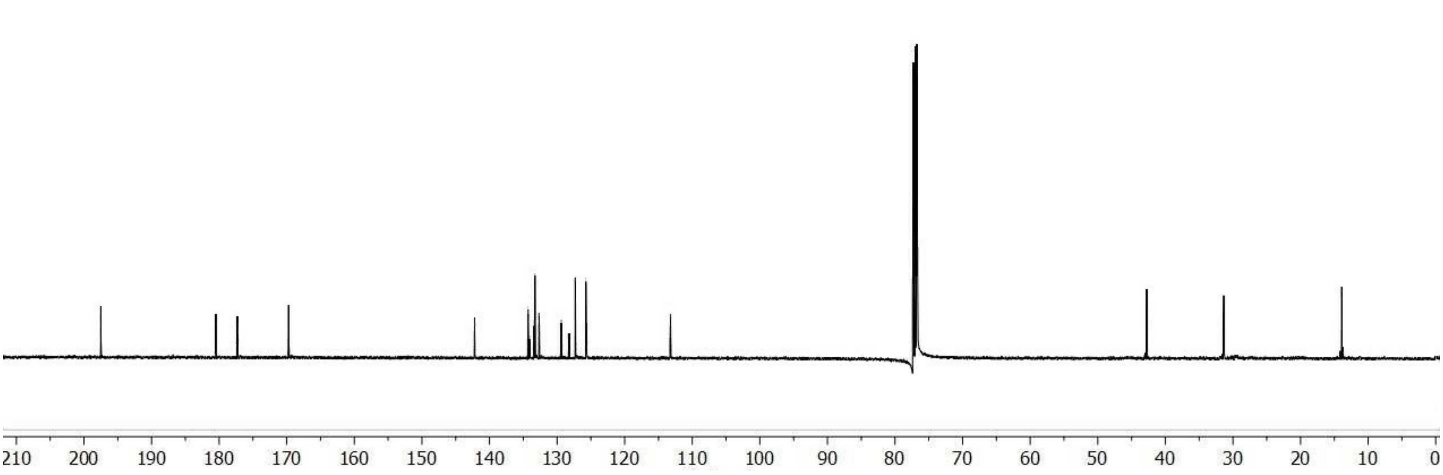

^{1}H NMR (500MHz, $CDCl_3$)

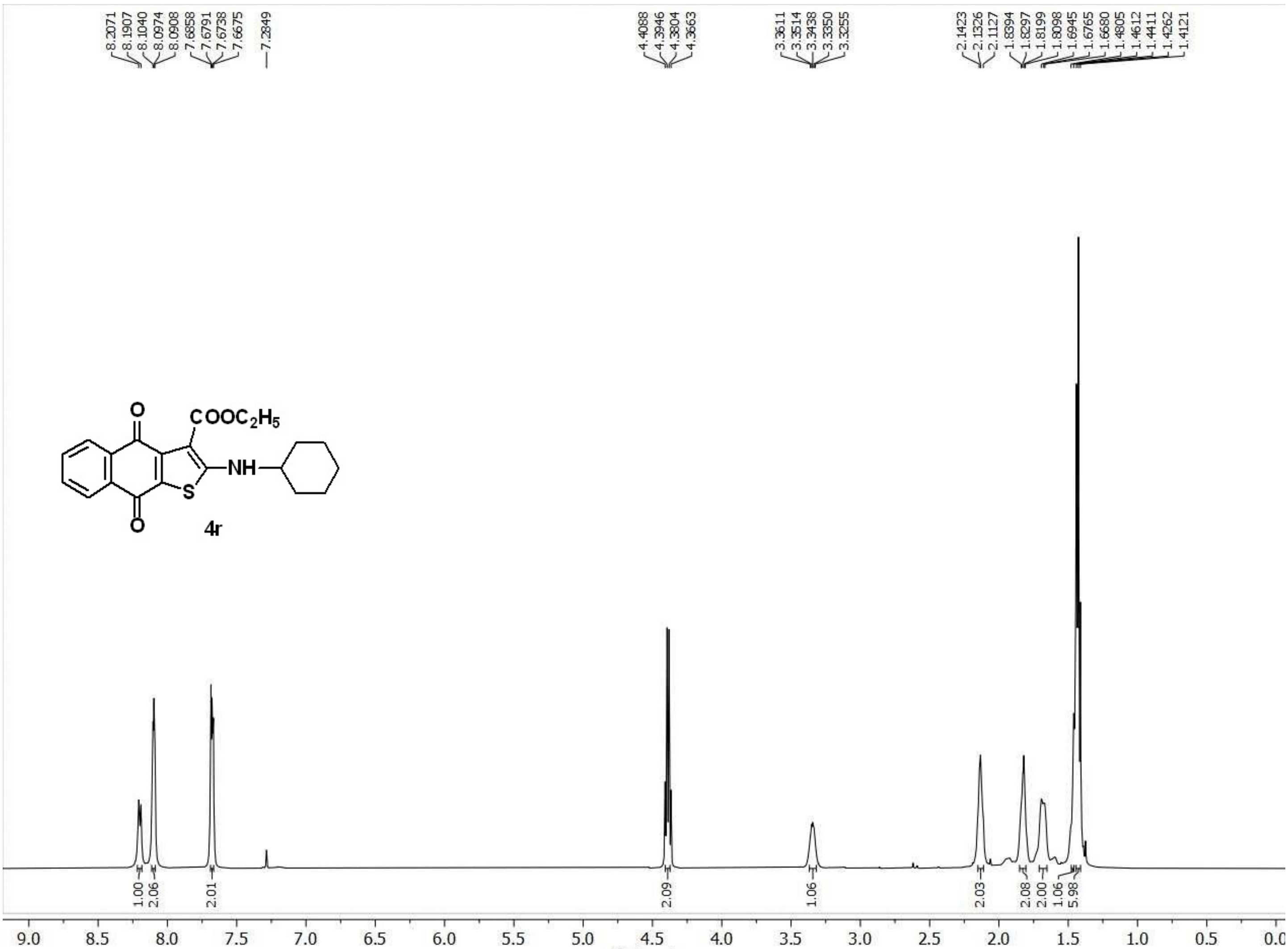

^{13}C NMR (126MHz, $CDCl_3$)

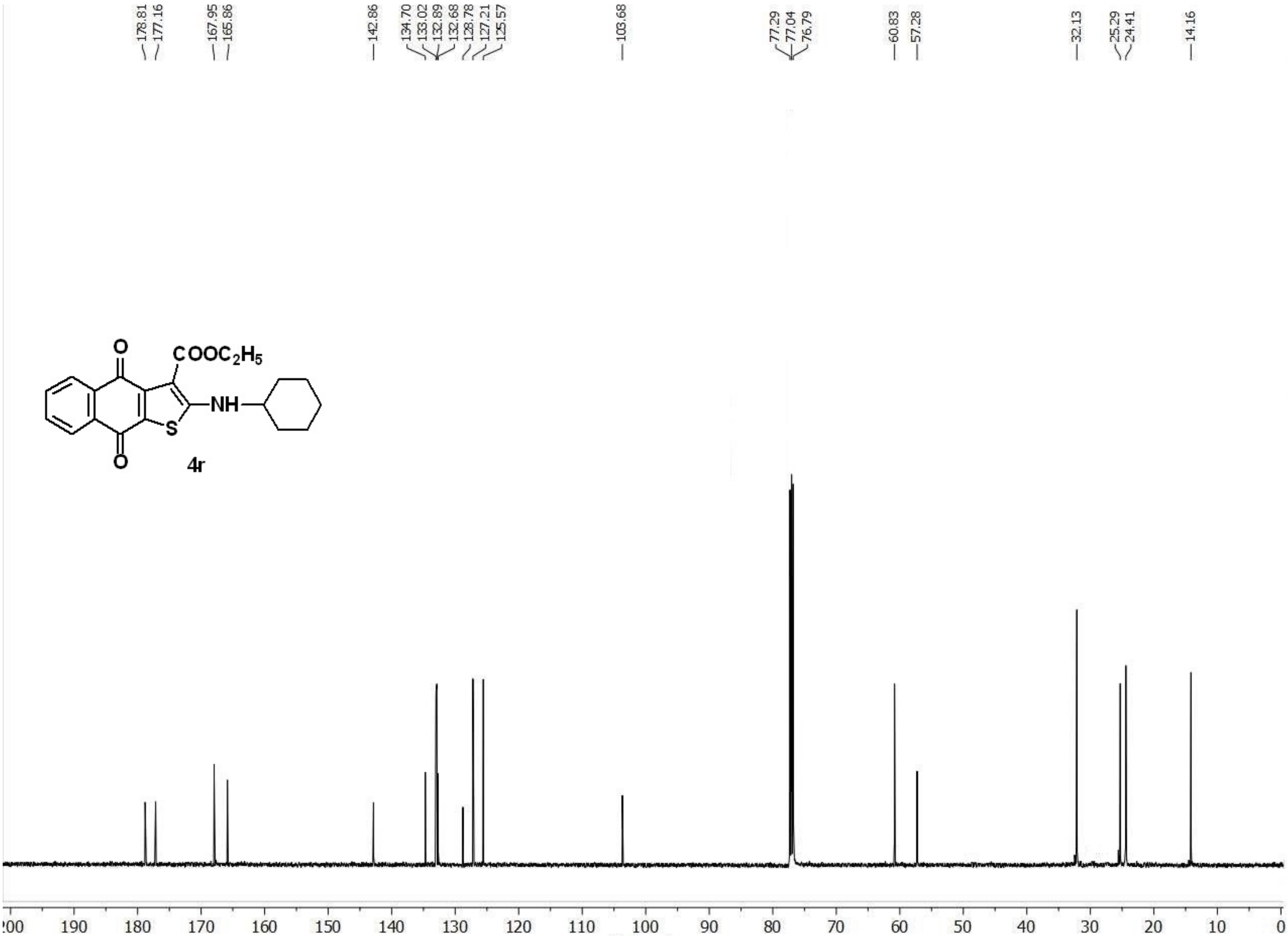

^{1}H NMR (600MHz, $CDCl_3$)

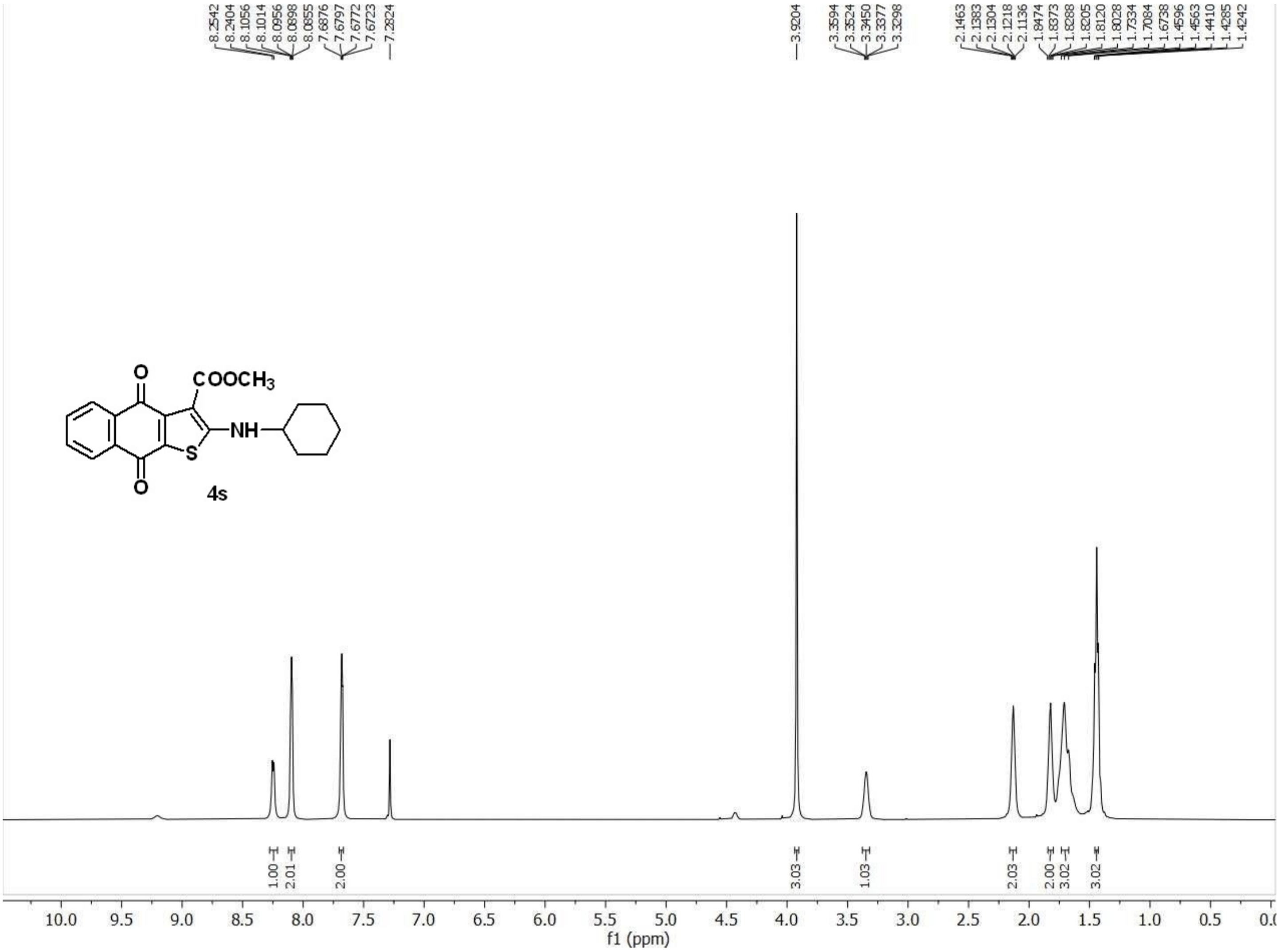

^{13}C NMR (151MHz, $CDCl_3$)

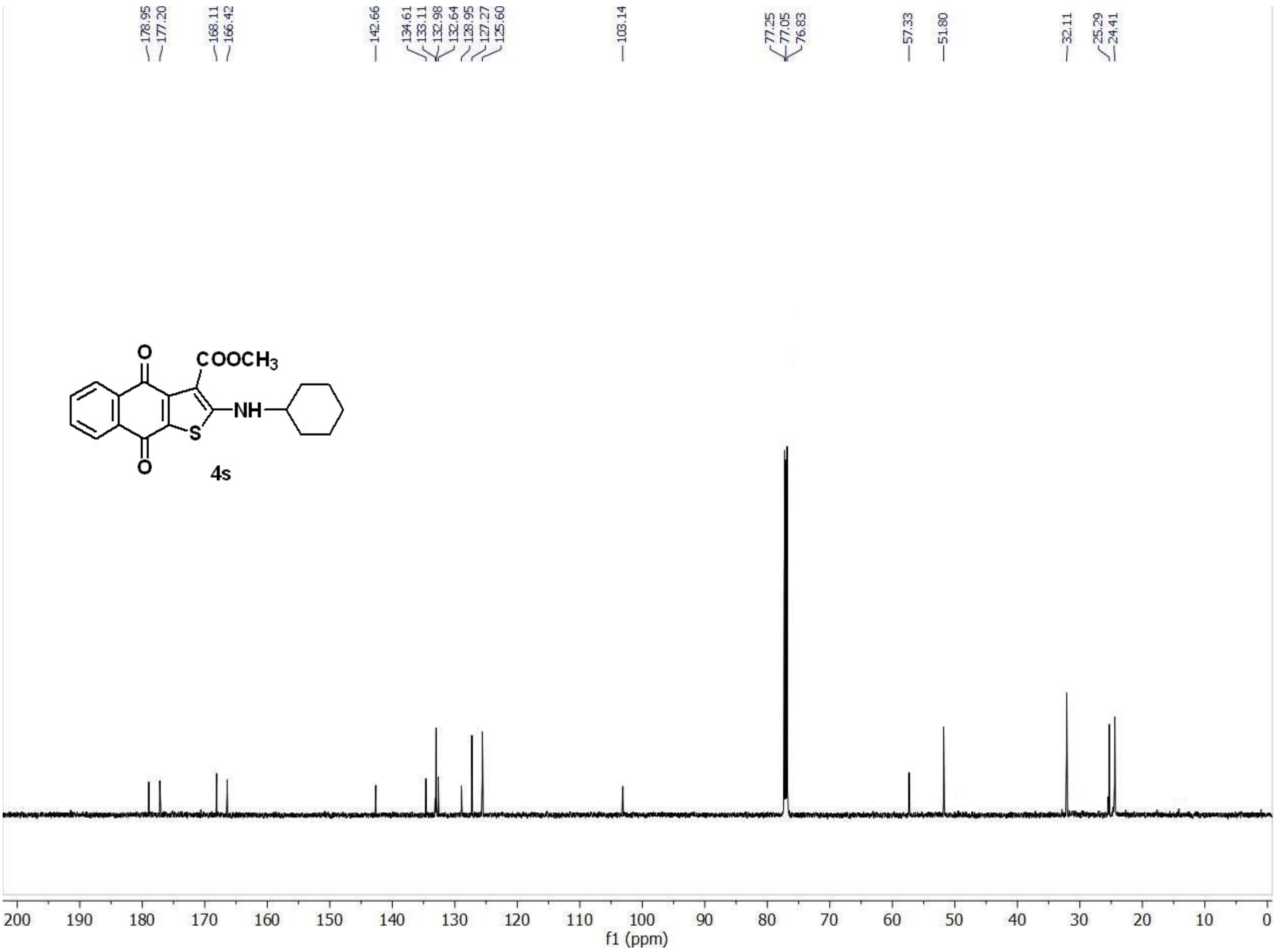

^{1}H NMR (600MHz, $CDCl_3$)

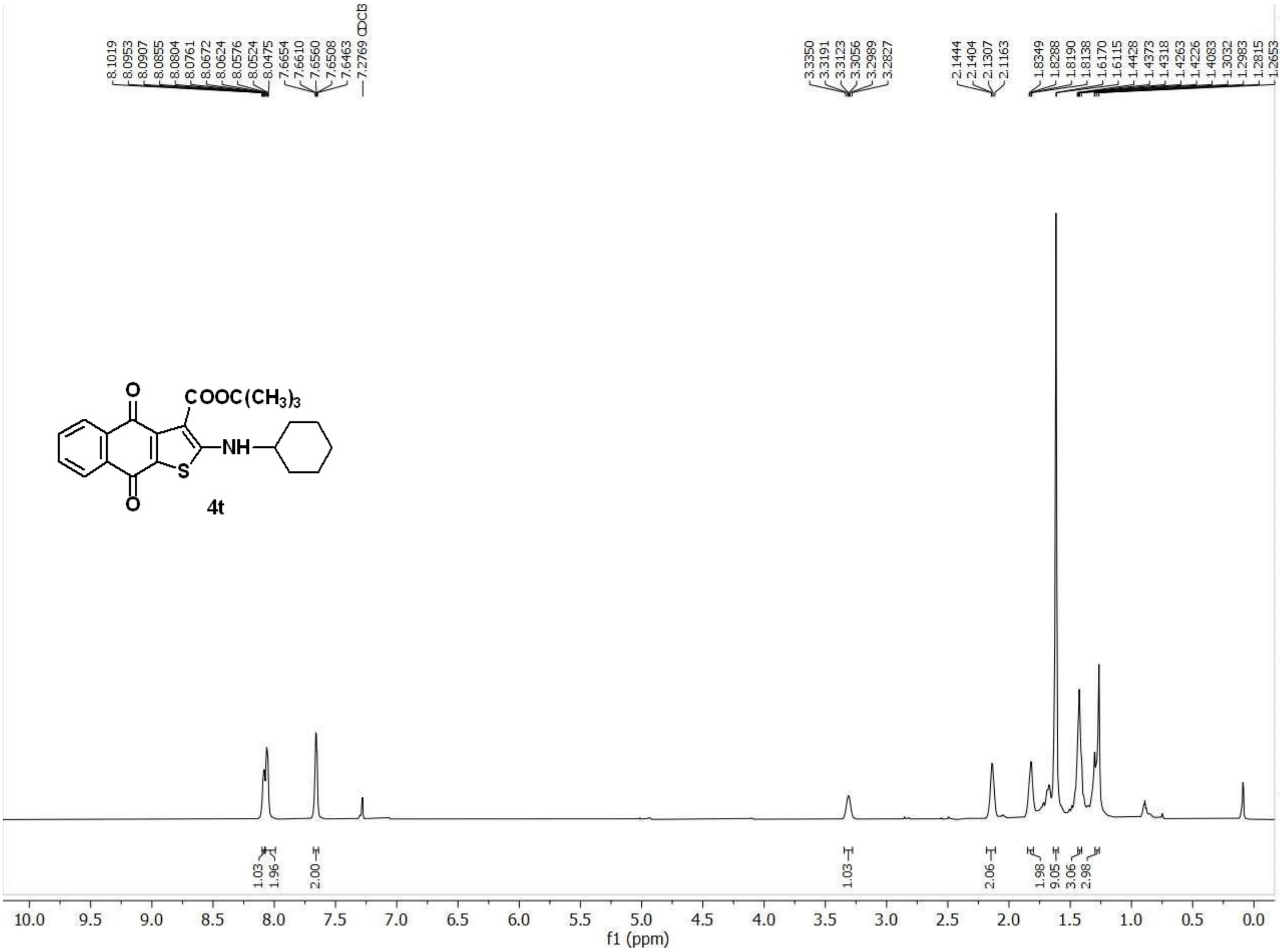

^{13}C NMR (151MHz, $CDCl_3$)

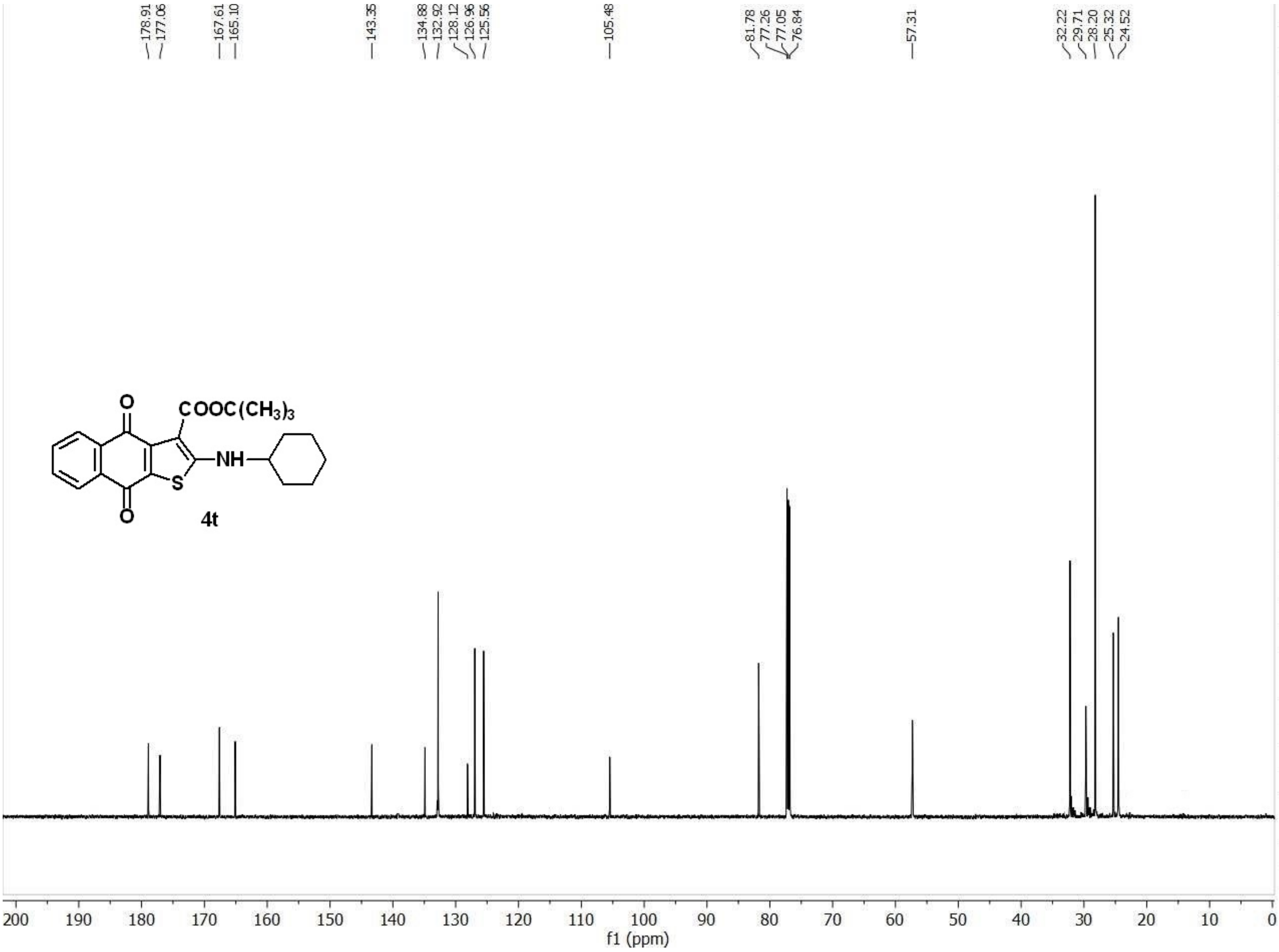

^{1}H NMR (500MHz, $CDCl_3$)

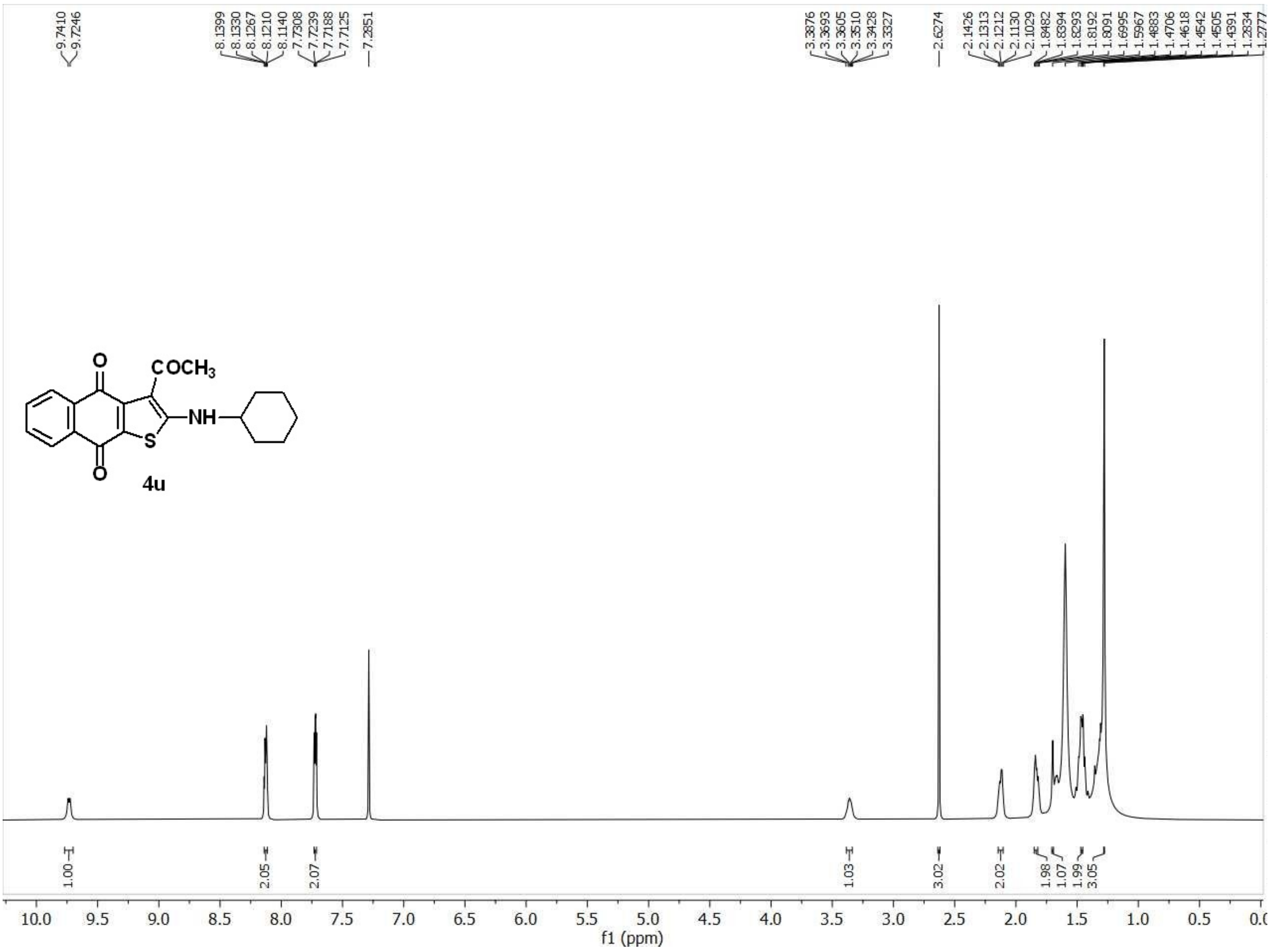

^{13}C NMR (126MHz, $CDCl_3$)

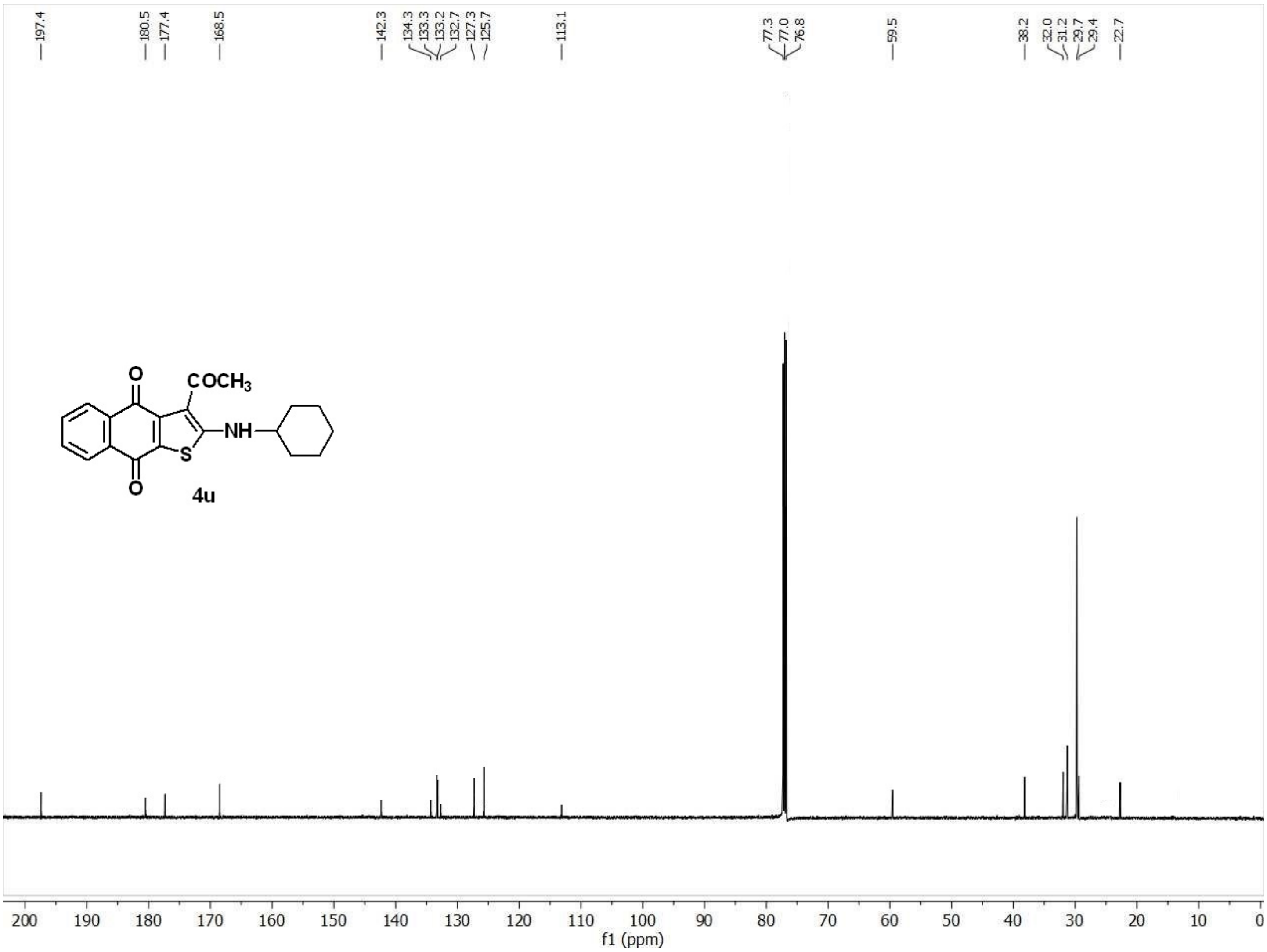

^{1}H NMR (600MHz, $CDCl_3$)

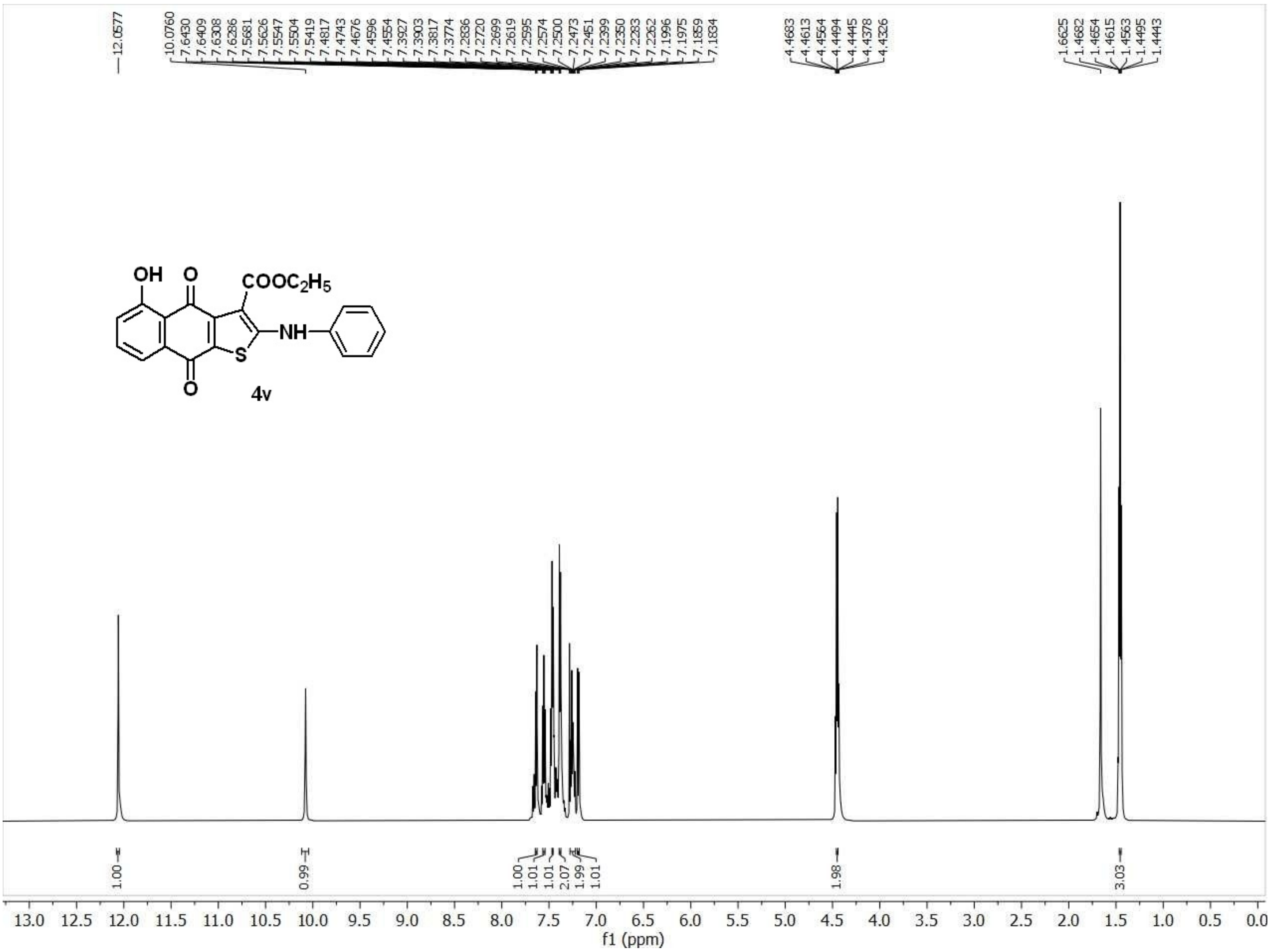

^{13}C NMR (101MHz, $CDCl_3$)

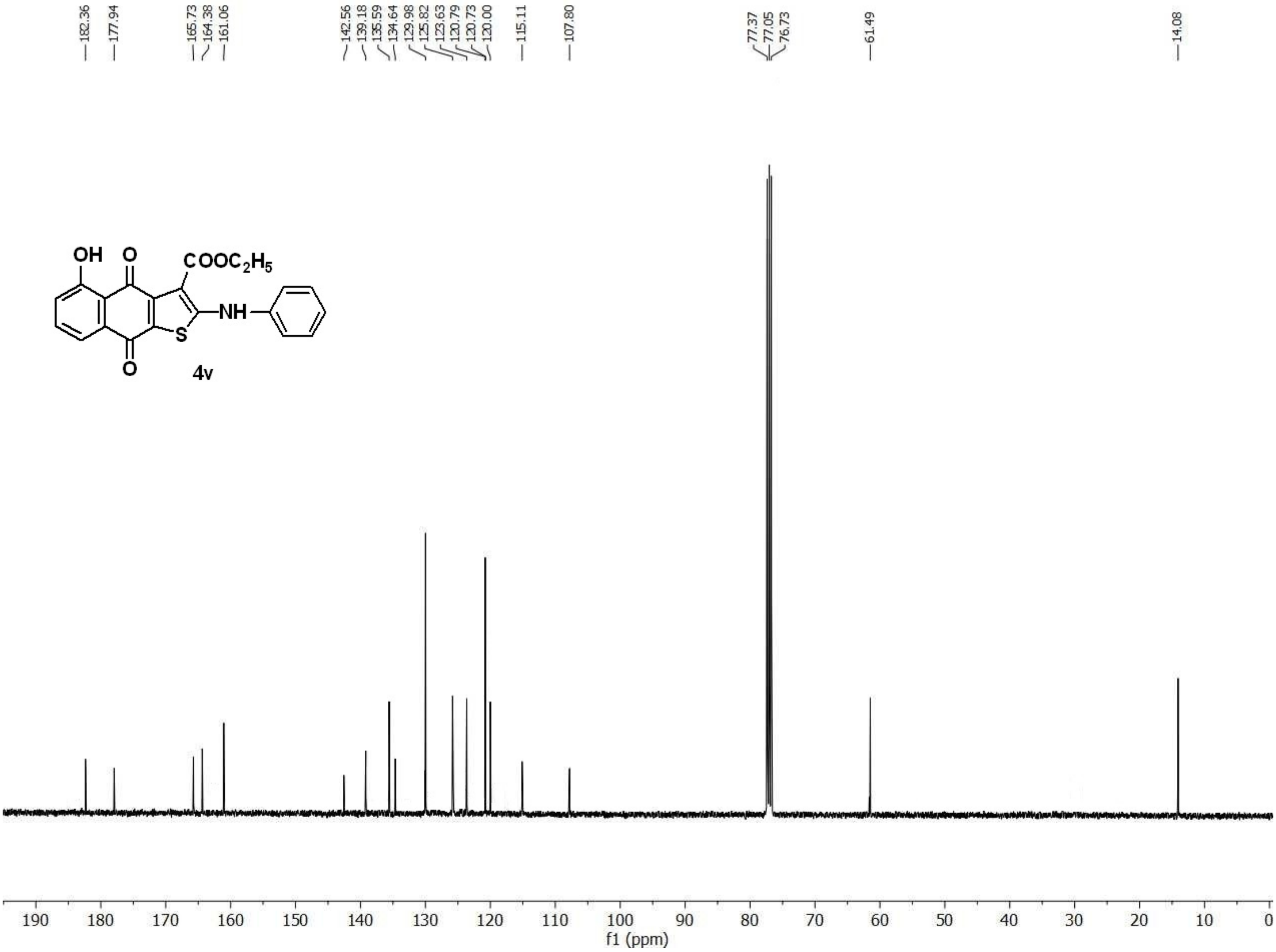

HRMS, LCMS, and Mass Spectral data

Sample Information

**Name**	ArD-CD-S86	**Data File Path**	D:\MassHunter\Data\OCT-2022\ArD-CD-S86.d
**Sample ID**		**Acq. Time (Local)**	11-10-2022 2.10.57 PM (UTC+05:30)
**Instrument**	LCMS QTOF	**Method Path (Acq)**	D:\MassHunter\Methods\6545XT checkout\Methods\TRAINING\MS SCAN_AB_POS_100-1000_4000-100-120.m
**MS Type**	QTOF	**Version (Acq SW)**	6200 series TOF/6500 series Q-TOF B.09.00 (B9044.0)
**Inj. Vol. (ul)**	1	**IRM Status**	Success
**Position**	P1-E9	**Method Path (DA)**	
**Plate Pos.**		**Target Source Path**	
**Operator**		**Result Summary**	

Sample Spectra

+ Scan (rt: 0.304 min)

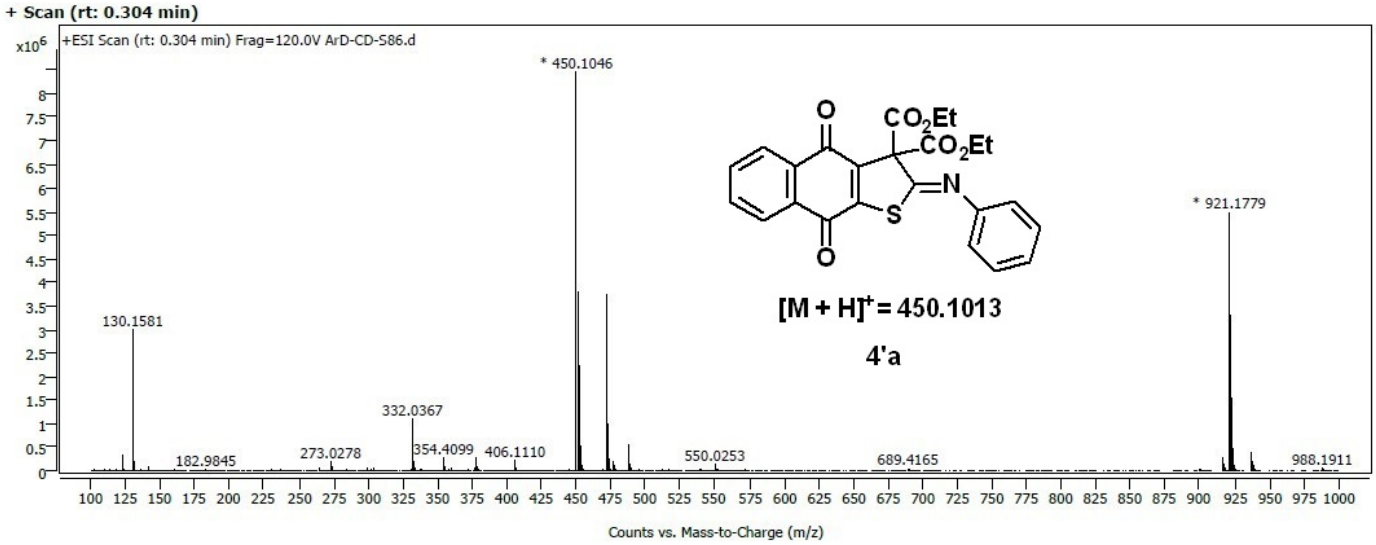

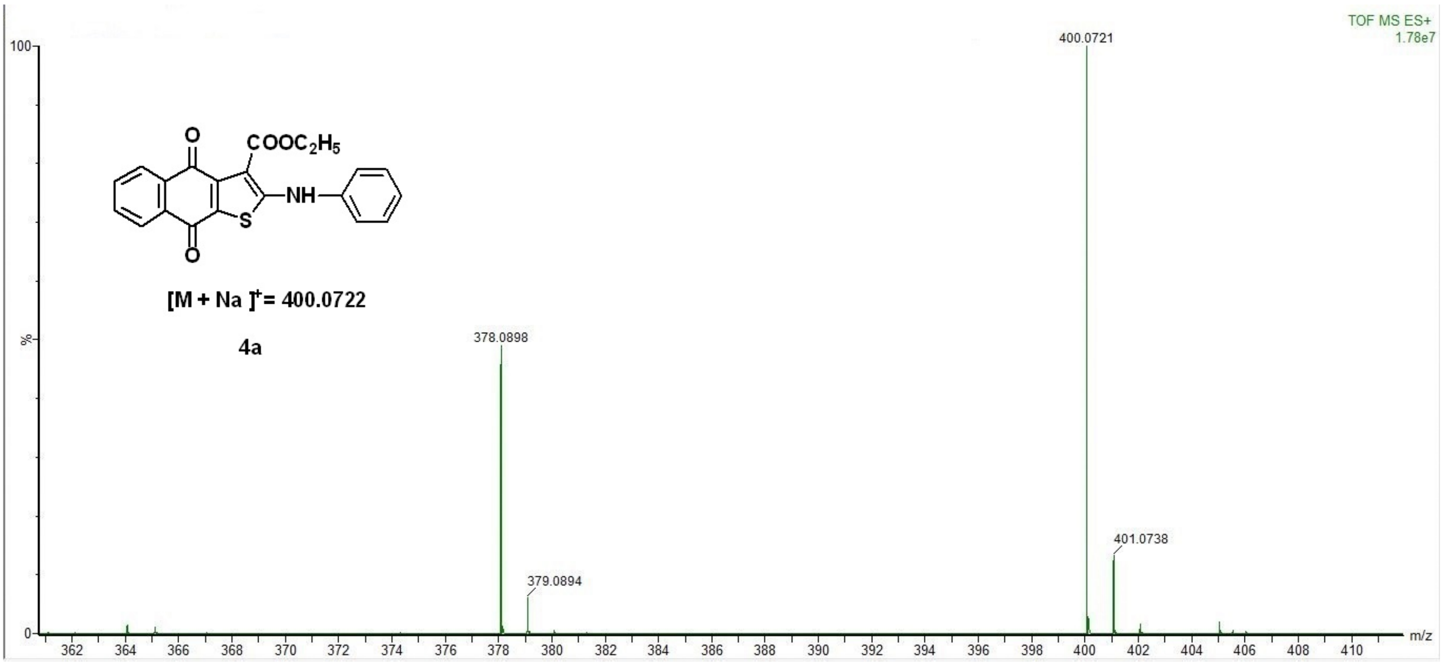

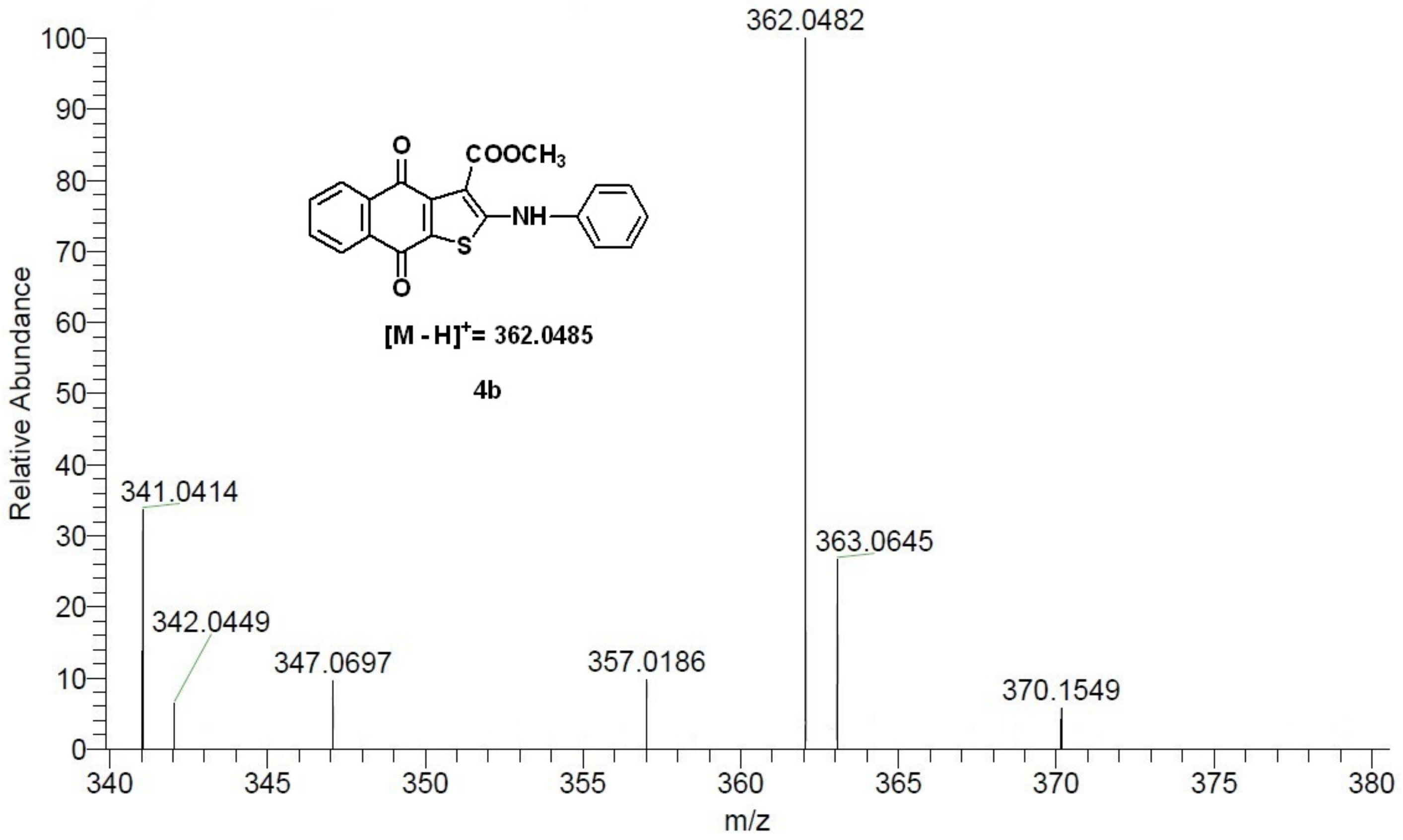
362.0482
COOCH3
NH
S
[M - H]+= 362.0485
4b
341.0414
363.0645
342.0449
347.0697
357.0186
370.1549
Relative Abundance
m/z

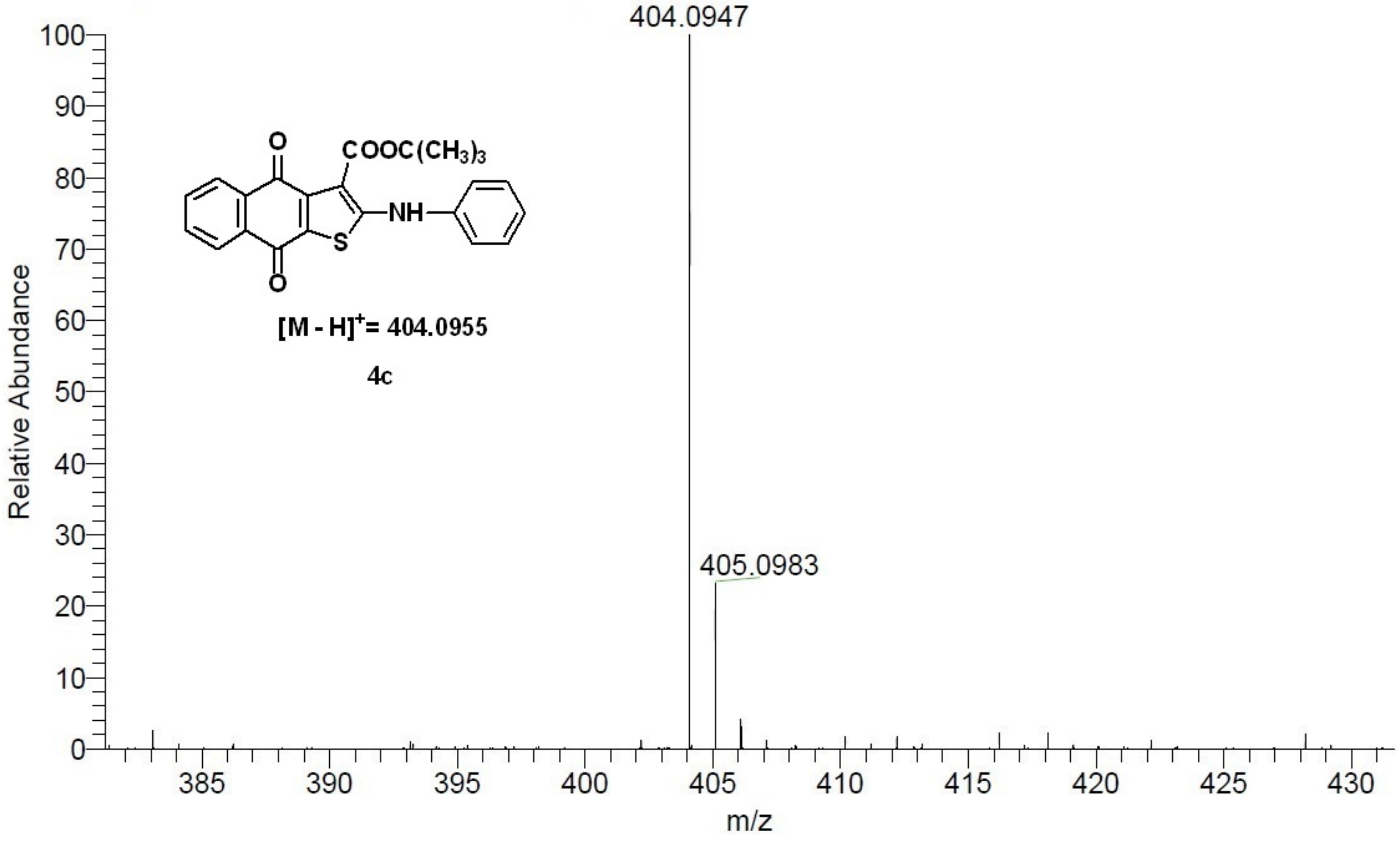
404.0947
COOC(CH3)3
NH
S
[M - H]+= 404.0955
4c
405.0983
Relative Abundance
m/z

Sample Information

**Name**	ArD-CD-S98	**Data File Path**	D:\MassHunter\Data\OCT-2022\ArD-CD-S98.d
**Sample ID**		**Acq. Time (Local)**	11-10-2022 2.07.13 PM (UTC+05:30)
**Instrument**	LCMS QTOF	**Method Path (Acq)**	D:\MassHunter\Methods\6545XT checkout\Methods\TRAINING\MS SCAN_AB_POS_100-1000_4000-100-120.m
**MS Type**	QTOF	**Version (Acq SW)**	6200 series TOF/6500 series Q-TOF B.09.00 (B9044.0)
**Inj. Vol. (ul)**	1	**IRM Status**	Success
**Position**	P1-E8	**Method Path (DA)**	
**Plate Pos.**		**Target Source Path**	
**Operator**		**Result Summary**	

Sample Spectra

+ Scan (rt: 0.311 min)

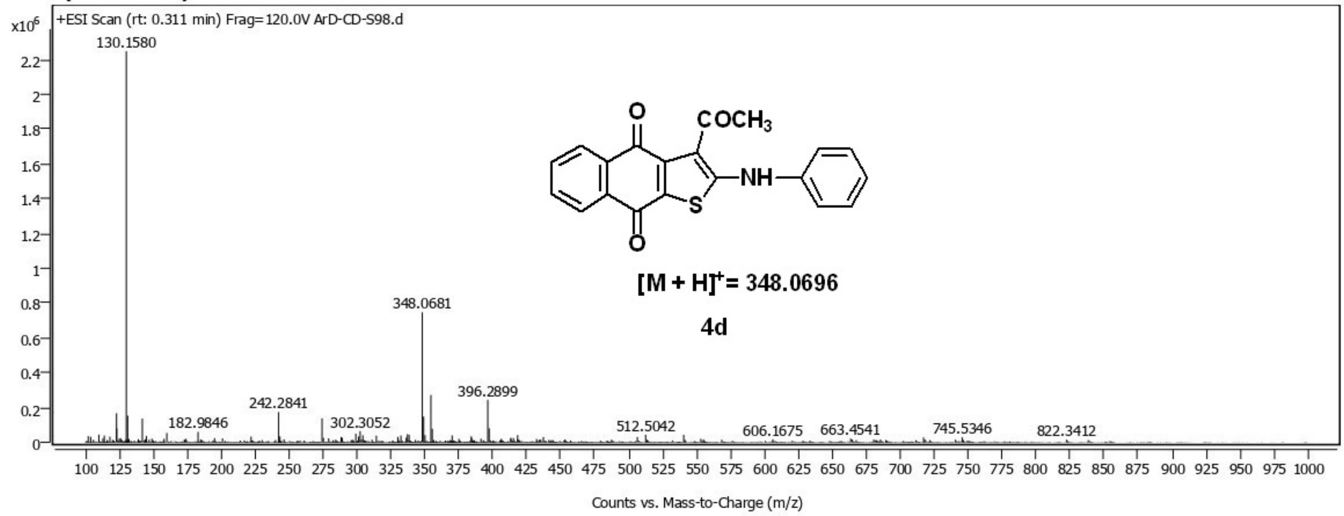

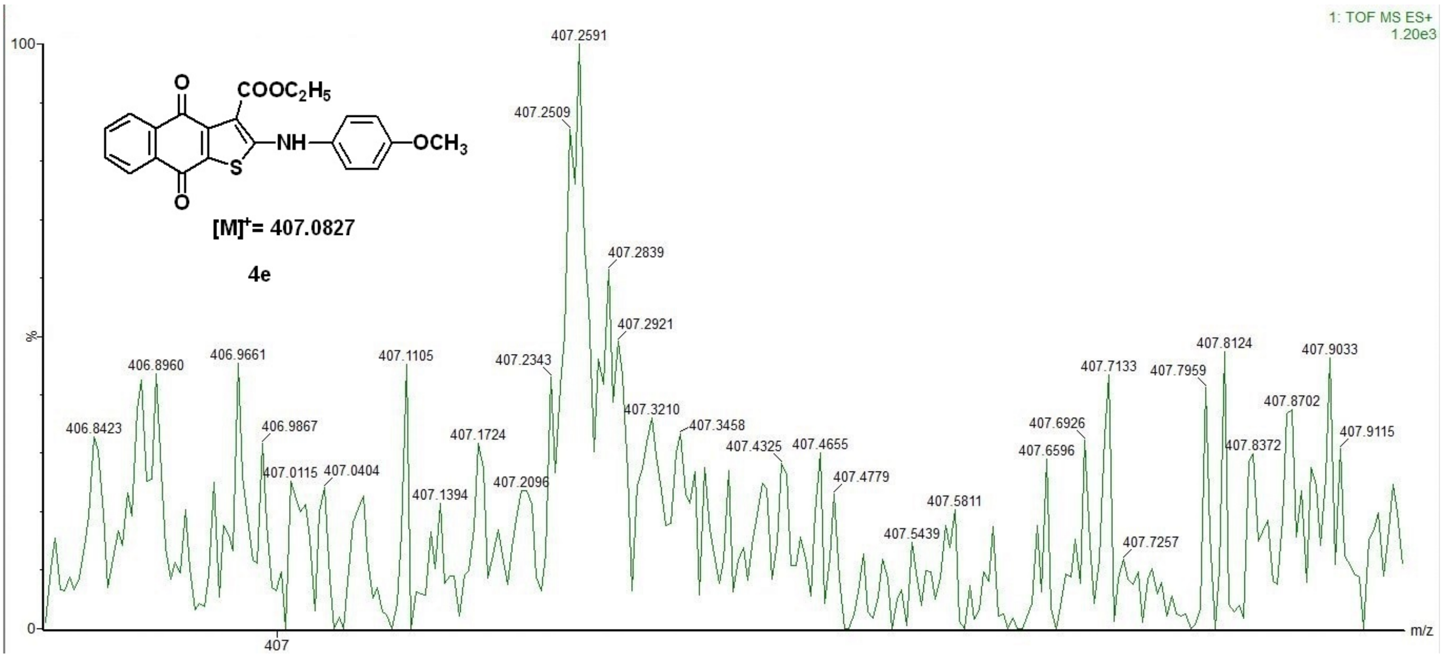

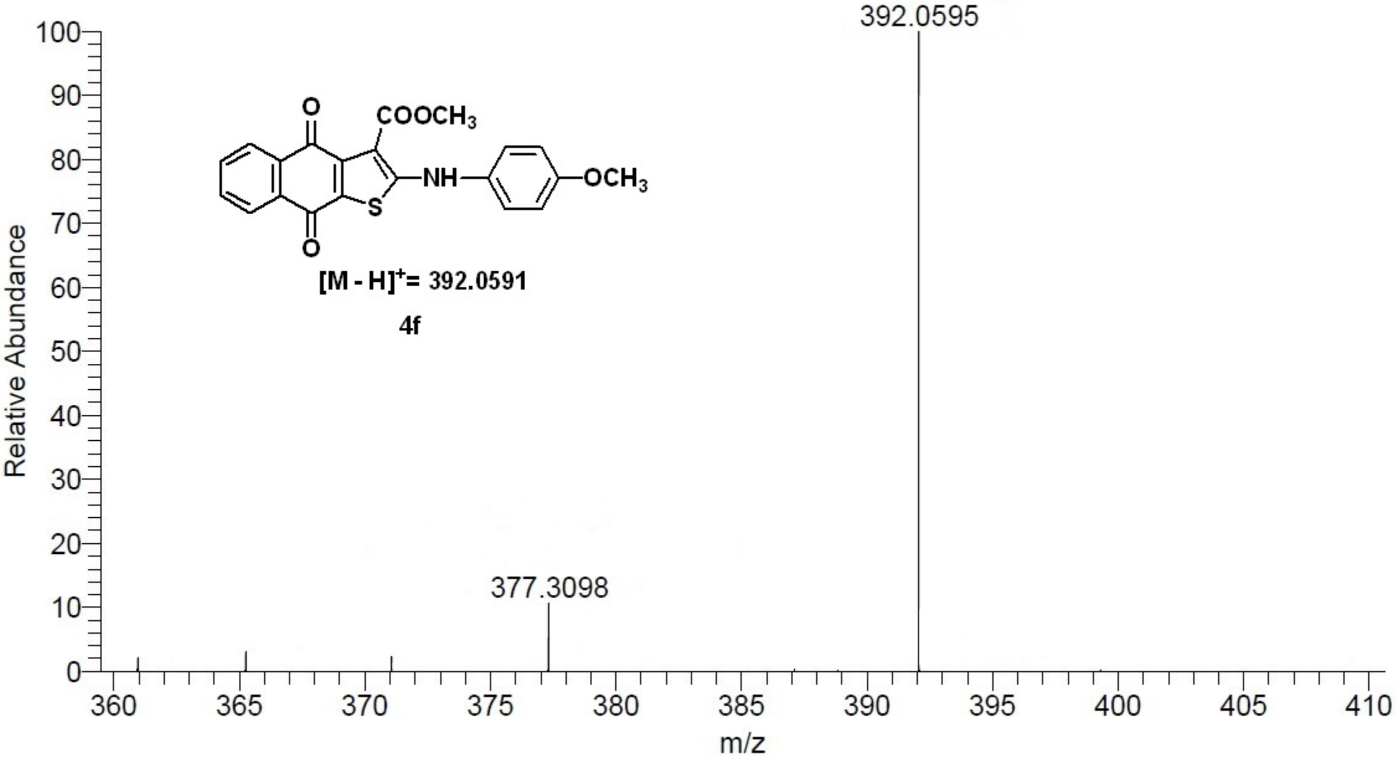

392.0595
377.3098
Relative Abundance
m/z
COOCH3
NH
OCH3
S
[M - H]+= 392.0591
4f

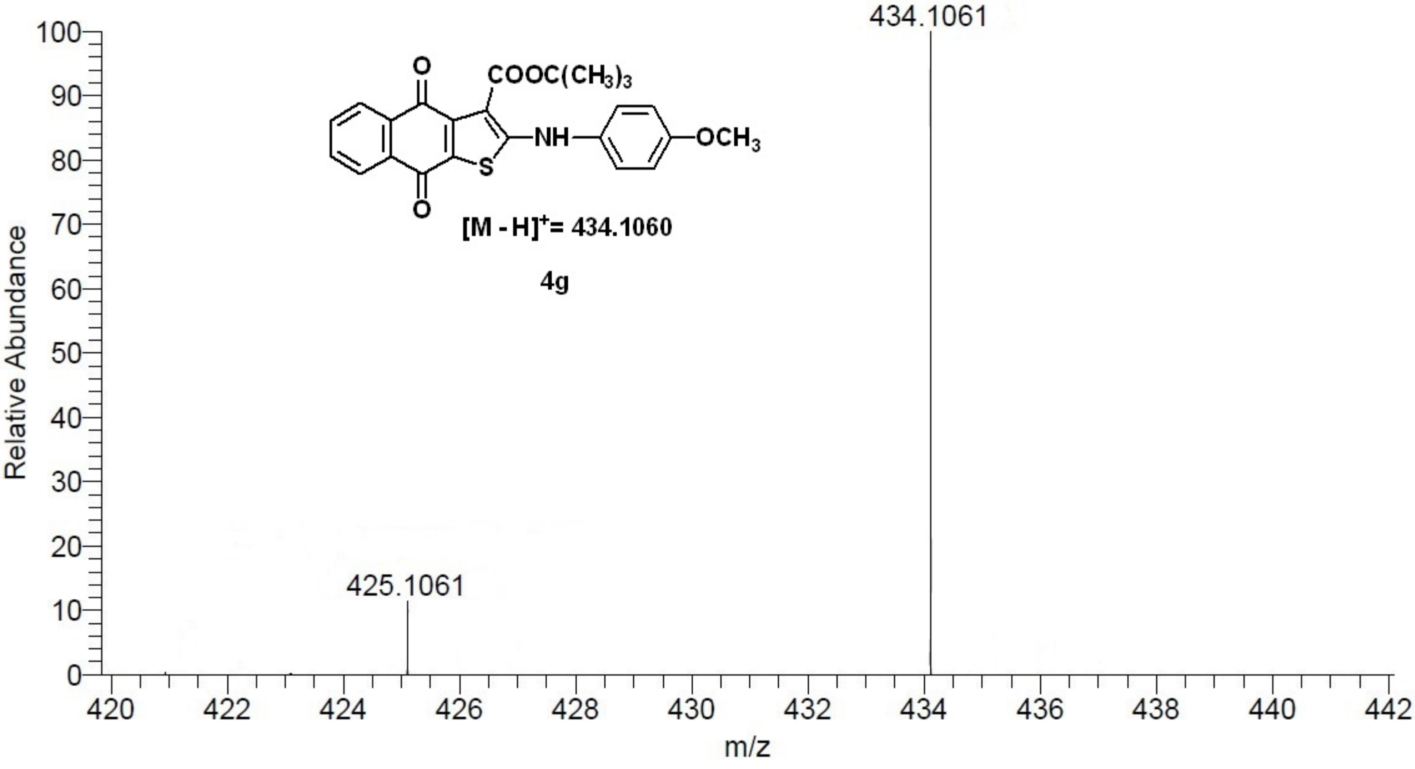

434.1061
425.1061
Relative Abundance
m/z
COOC(CH3)3
NH
OCH3
S
[M - H]+= 434.1060
4g

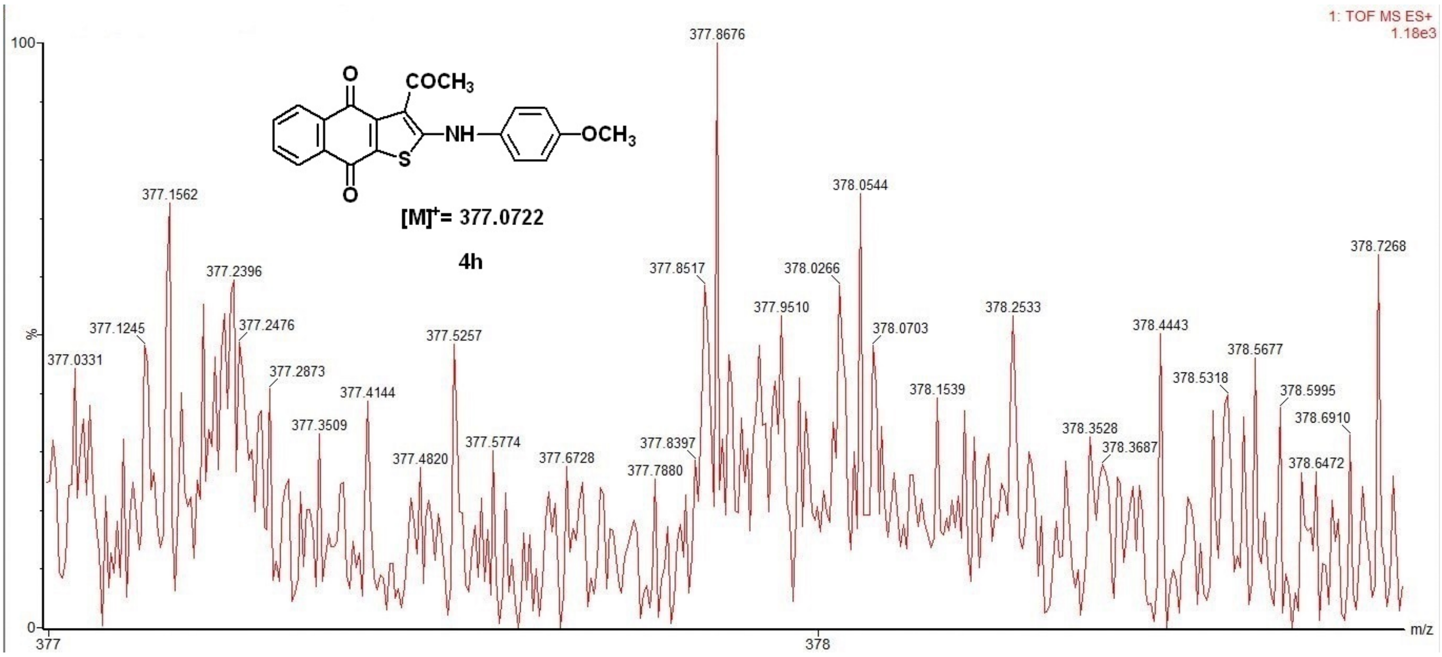

1: TOF MS ES+
1.18e3
[M]+= 377.0722
4h
377.8676
378.0544
377.1562
378.7268
377.2396
377.8517
378.0266
377.9510
378.2533
377.1245
377.2476
378.0703
378.4443
377.5257
378.5677
377.0331
377.2873
378.5318
377.4144
378.1539
378.5995
377.3509
378.6910
378.3528
377.5774
377.8397
378.3687
377.4820
377.6728
378.6472
377.7880
m/z

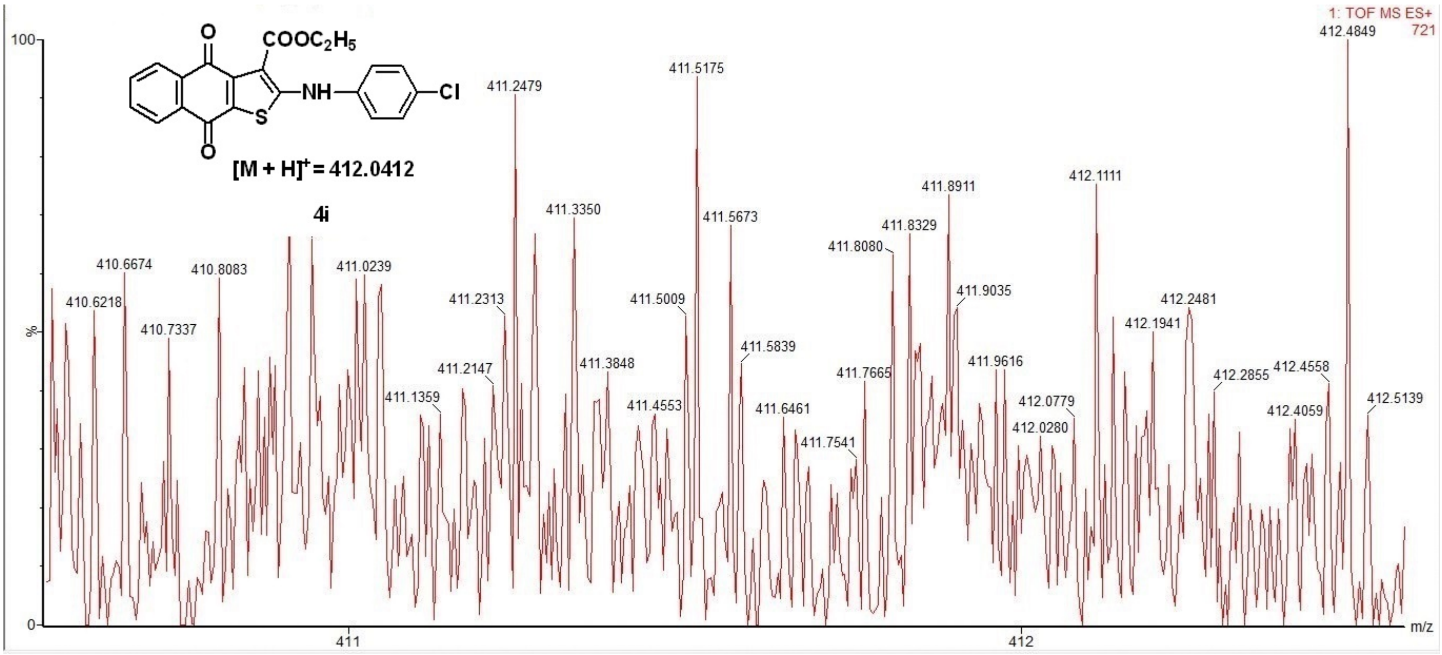

1: TOF MS ES+
721
412.4849
[M + H]+ = 412.0412
4i
411.5175
411.2479
412.1111
411.8911
411.3350
411.5673
411.8329
411.8080
410.6674
410.8083
411.0239
411.9035
410.6218
411.2313
411.5009
412.2481
412.1941
410.7337
411.5839
411.2147
411.3848
411.9616
411.7665
412.2855
412.4558
411.1359
412.0779
412.5139
411.4553
411.6461
412.4059
412.0280
411.7541
m/z

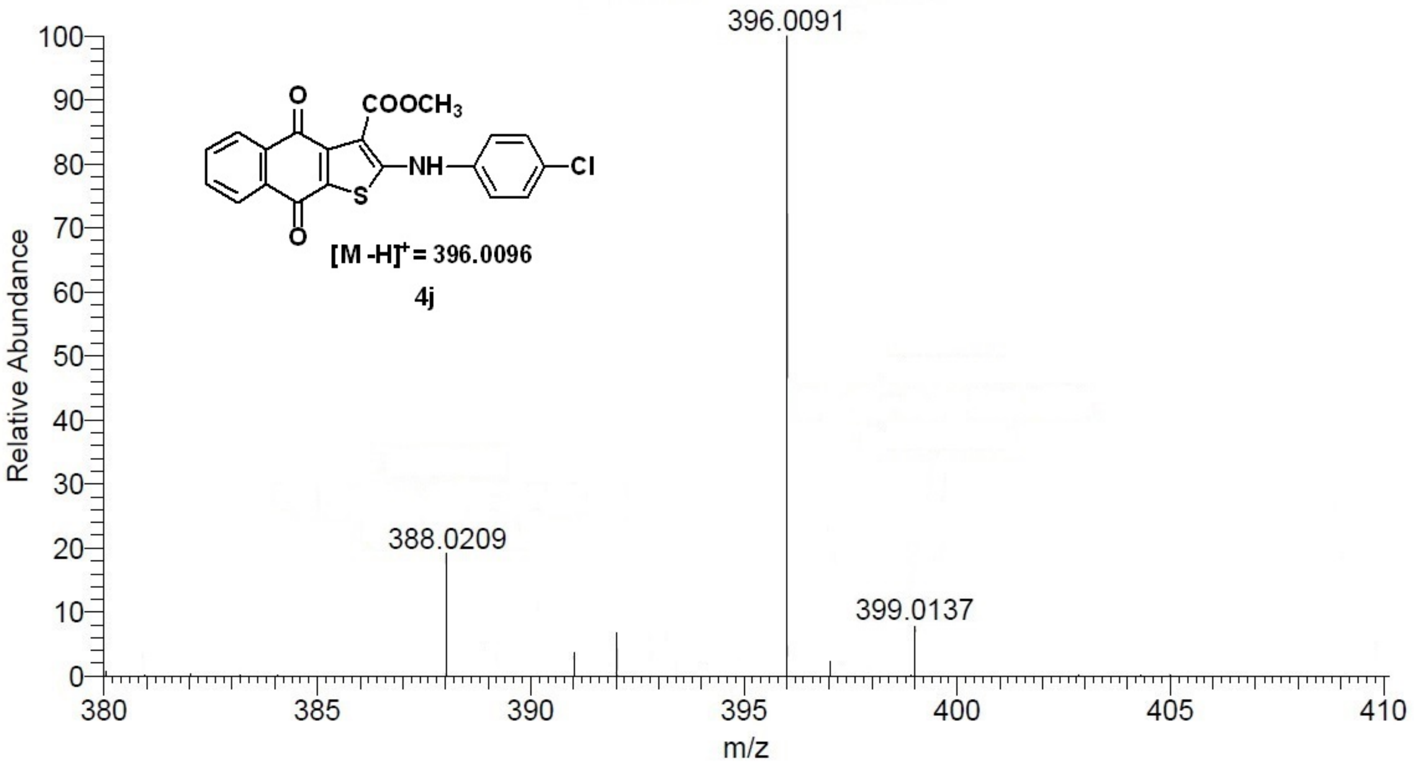
396.0091
COOCH3
NH
Cl
S
[M -H]+ = 396.0096
4j
388.0209
399.0137
Relative Abundance
m/z

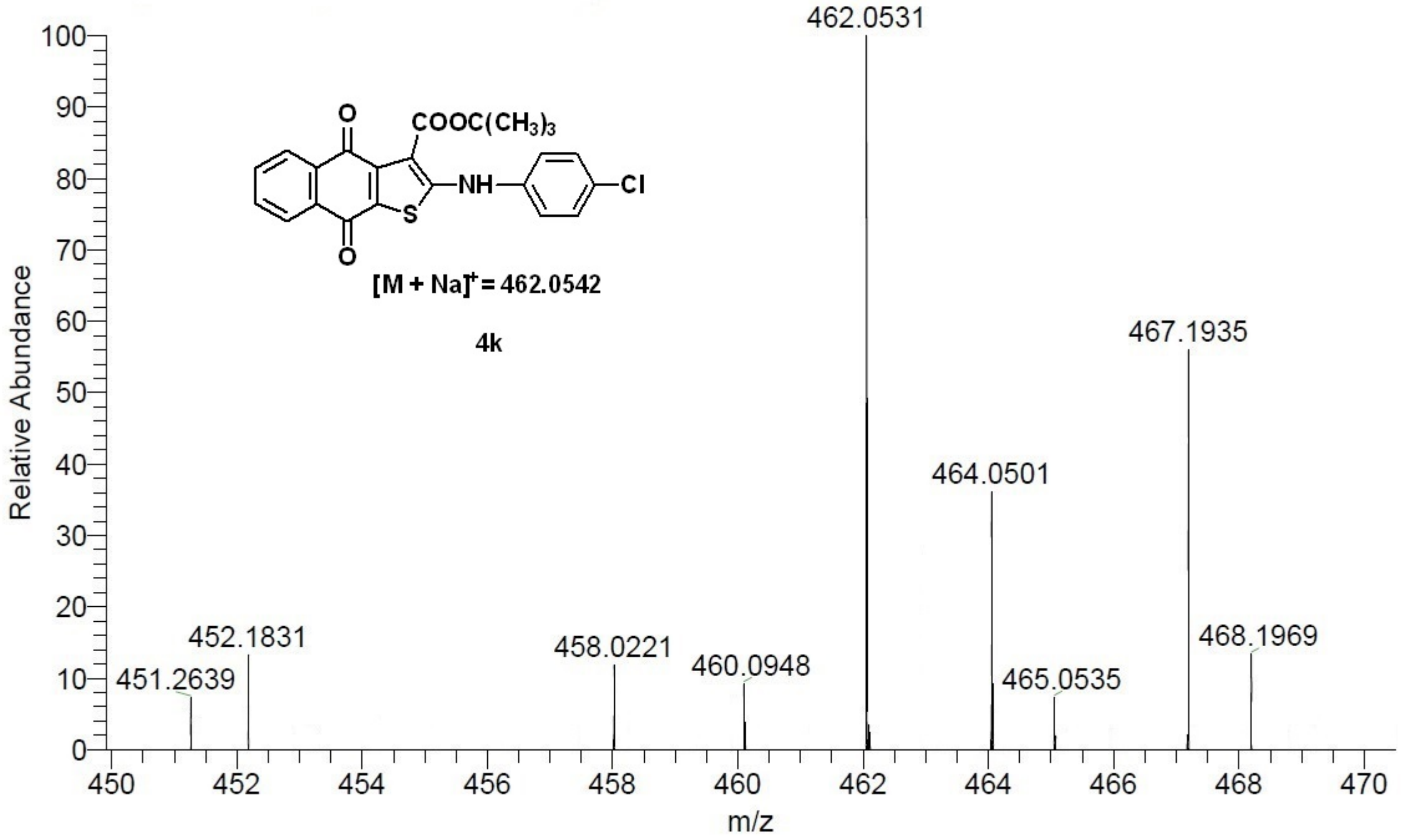
462.0531
COOC(CH3)3
NH
Cl
S
[M + Na]+ = 462.0542
4k
467.1935
464.0501
452.1831
451.2639
458.0221
460.0948
465.0535
468.1969
Relative Abundance
m/z

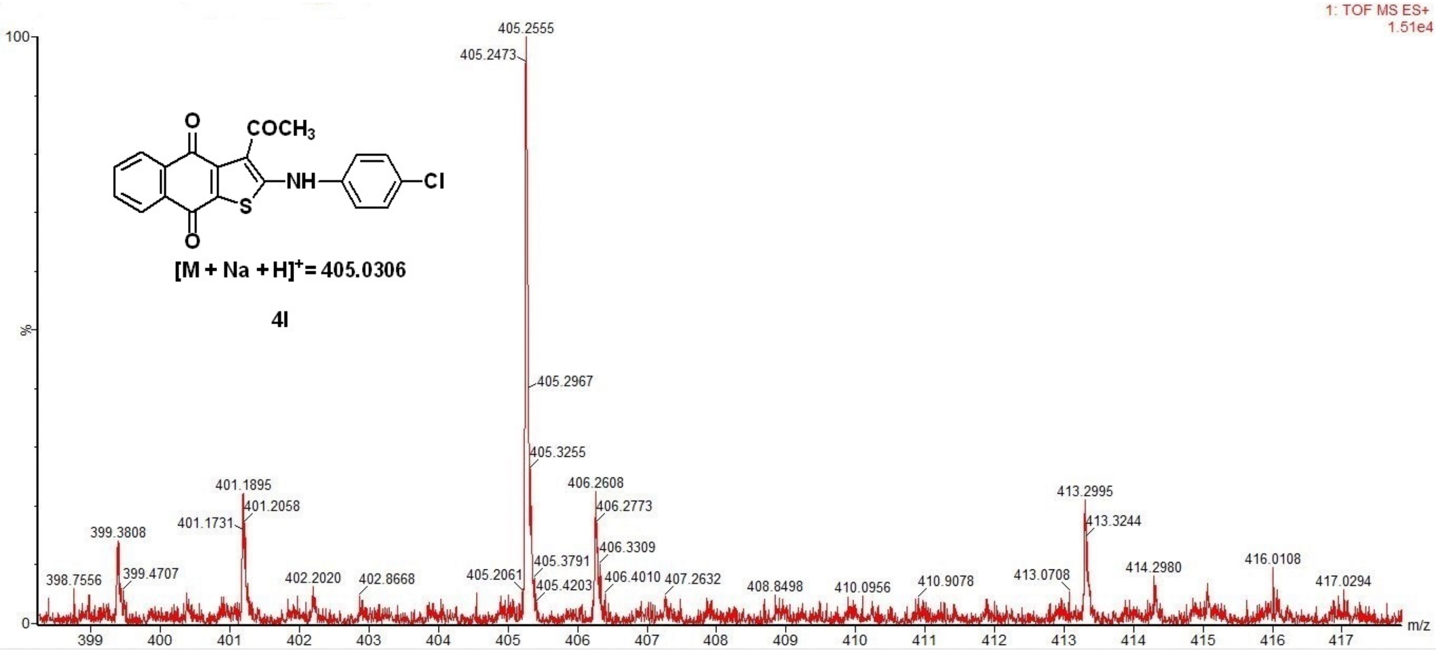
1: TOF MS ES+
1.51e4
COCH3
NH
Cl
S
O
[M + Na + H]+ = 405.0306
4l
405.2555
405.2473
405.2967
405.3255
406.2608
406.2773
401.1895
401.2058
401.1731
399.3808
413.2995
413.3244
%
m/z

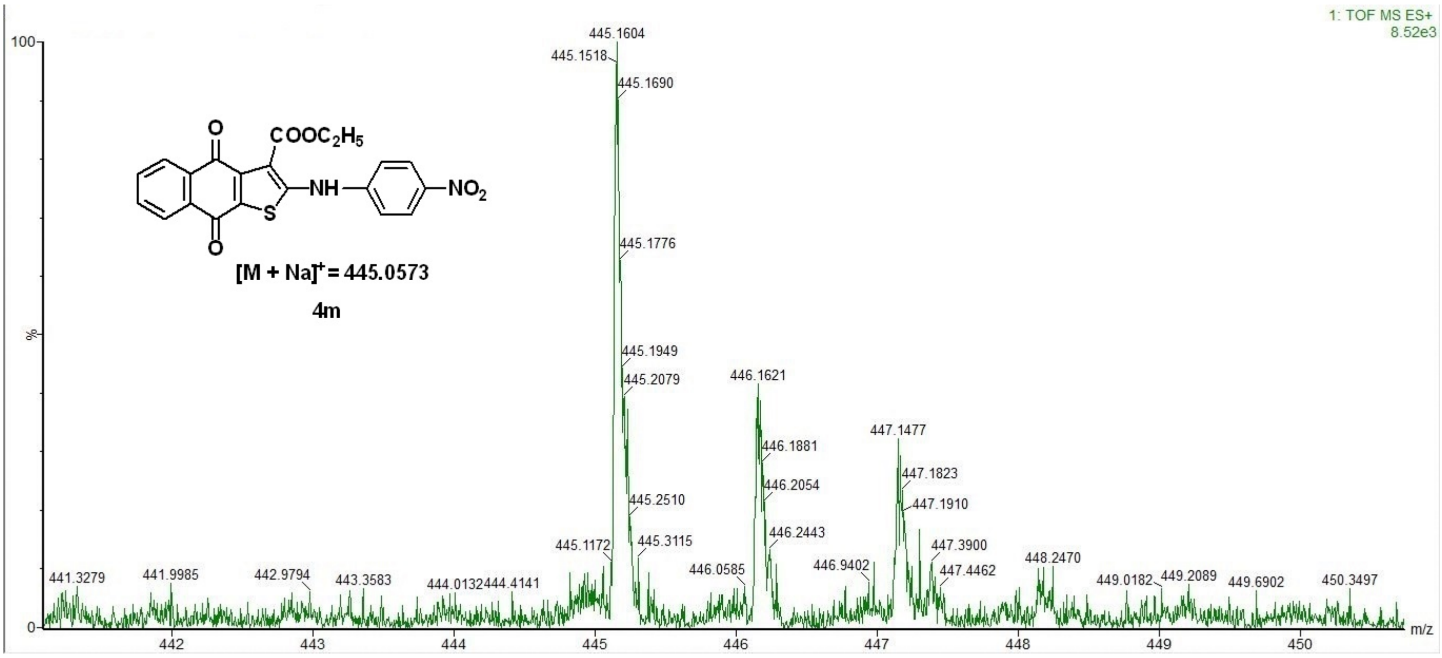
1: TOF MS ES+
8.52e3
COOC2H5
NH
NO2
S
O
[M + Na]+ = 445.0573
4m
445.1604
445.1518
445.1690
445.1776
446.1621
447.1477
%
m/z

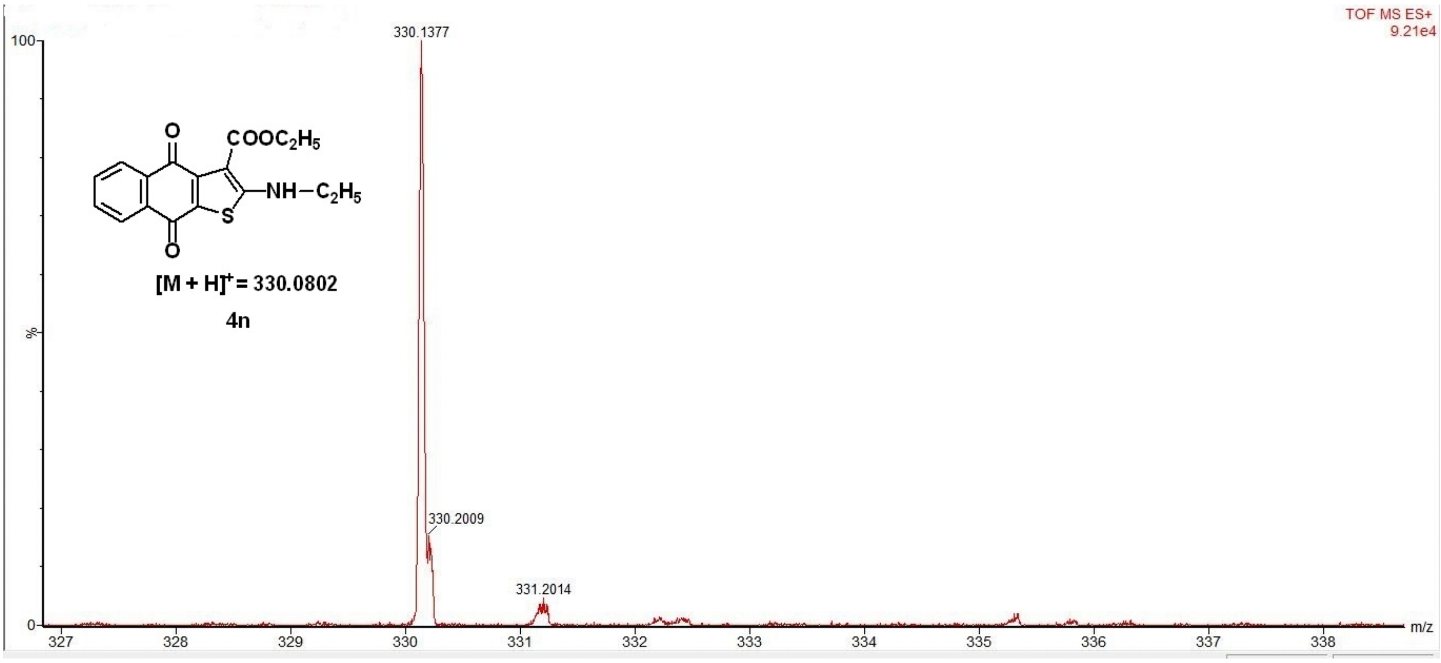
TOF MS ES+
9.21e4
330.1377
330.2009
331.2014
COOC2H5
NH−C2H5
S
O
[M + H]+ = 330.0802
4n
100
%
0
327
328
329
330
331
332
333
334
335
336
337
338
m/z

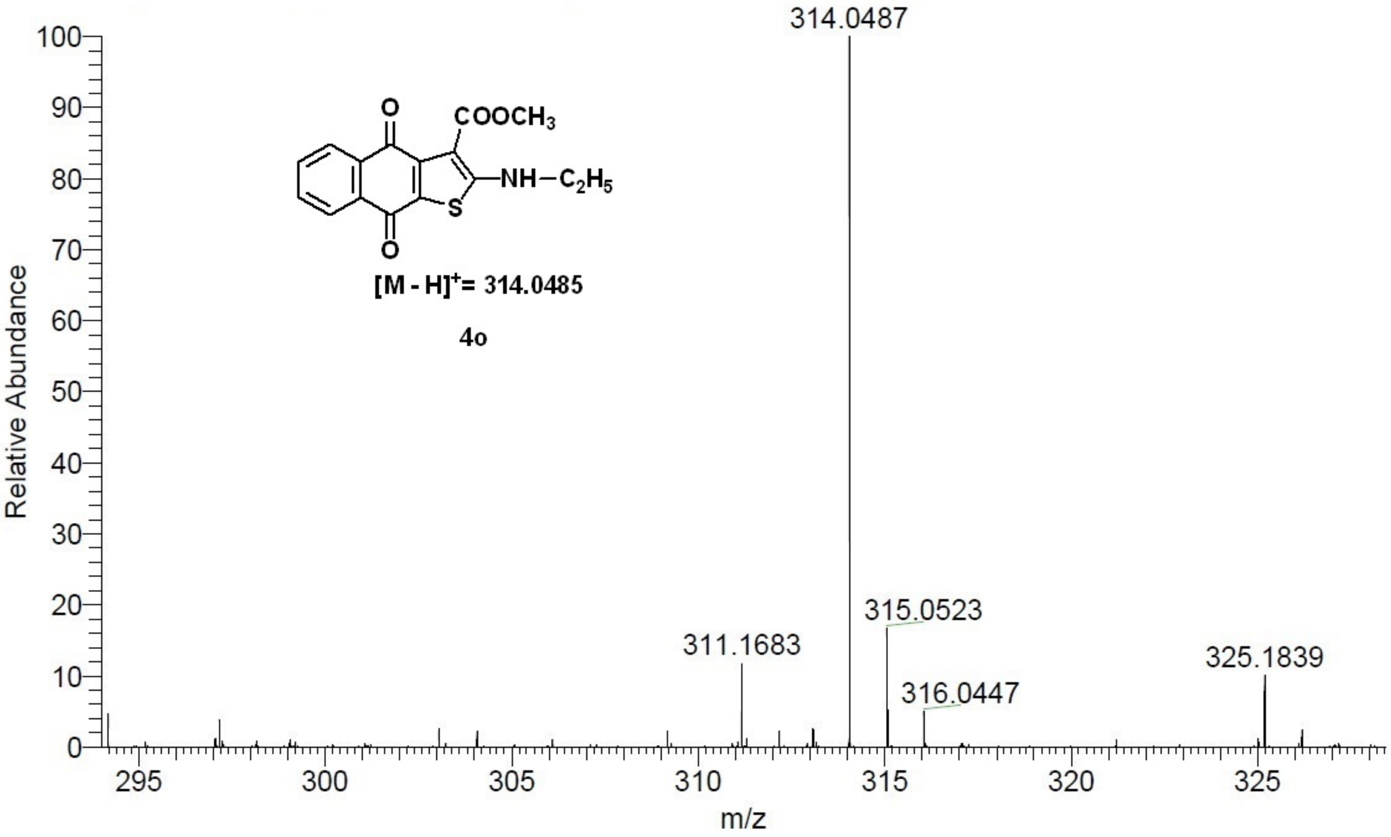
314.0487
COOCH3
NH−C2H5
S
O
[M - H]+= 314.0485
4o
315.0523
311.1683
316.0447
325.1839
Relative Abundance
100
90
80
70
60
50
40
30
20
10
0
295
300
305
310
315
320
325
m/z

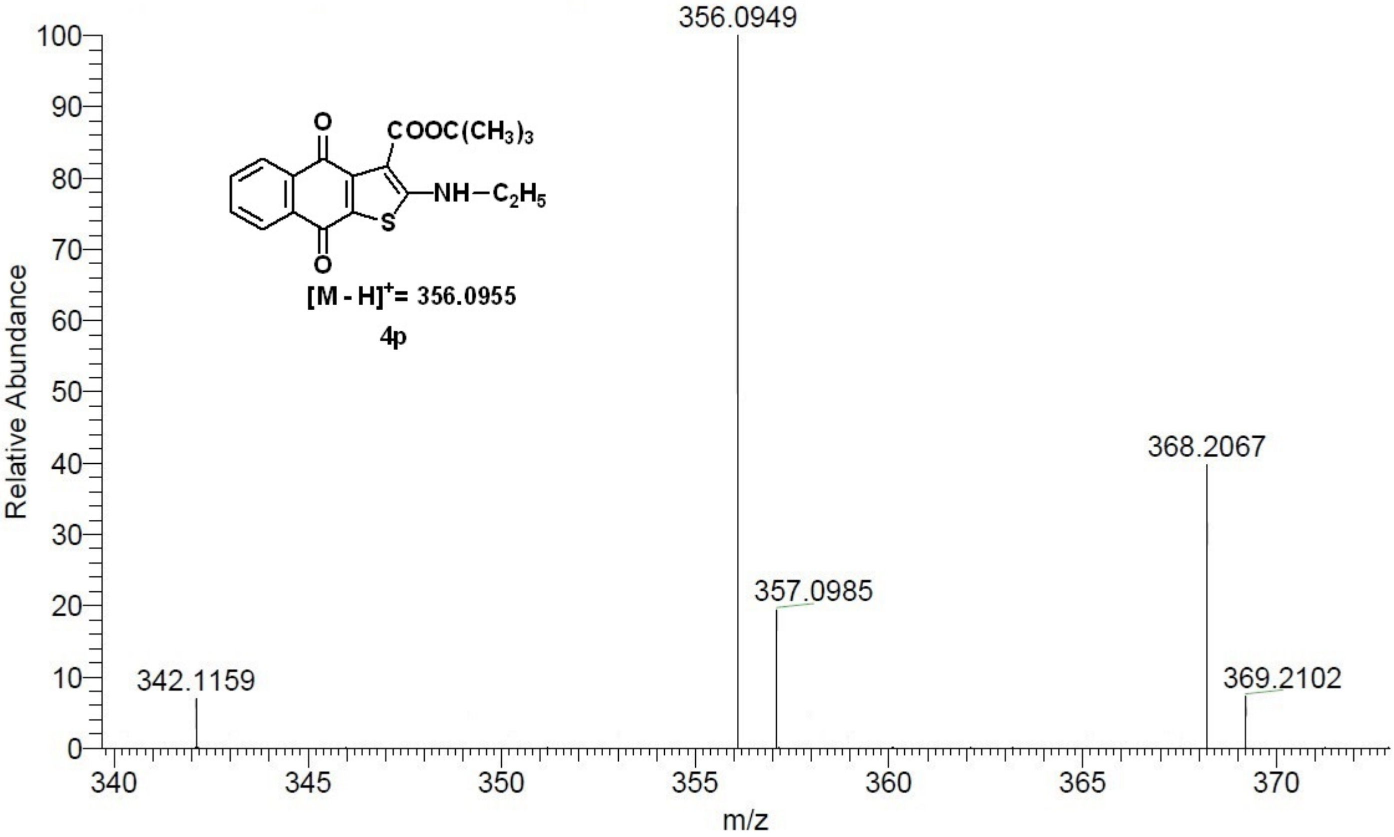
356.0949
100
90
80
70
60
50
40
30
20
10
0
Relative Abundance
COOC(CH3)3
NH−C2H5
S
[M - H]+= 356.0955
4p
368.2067
357.0985
342.1159
369.2102
340
345
350
355
360
365
370
m/z

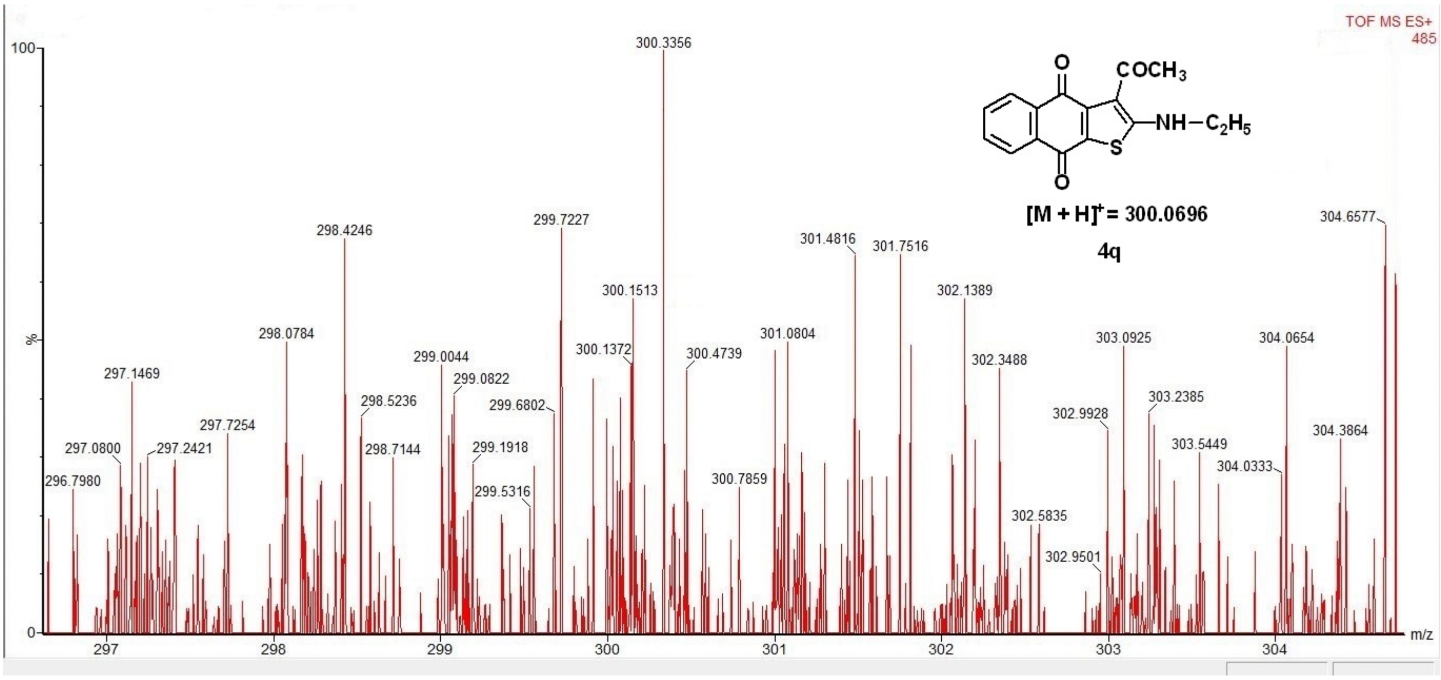
TOF MS ES+
485
100
%
0
300.3356
COCH3
NH−C2H5
S
[M + H]+= 300.0696
4q
304.6577
298.4246
299.7227
301.4816
301.7516
300.1513
302.1389
298.0784
301.0804
303.0925
304.0654
299.0044
300.1372
300.4739
302.3488
297.1469
299.0822
298.5236
299.6802
303.2385
302.9928
297.7254
304.3864
297.0800
297.2421
298.7144
299.1918
303.5449
304.0333
296.7980
299.5316
300.7859
302.5835
302.9501
297
298
299
300
301
302
303
304
m/z

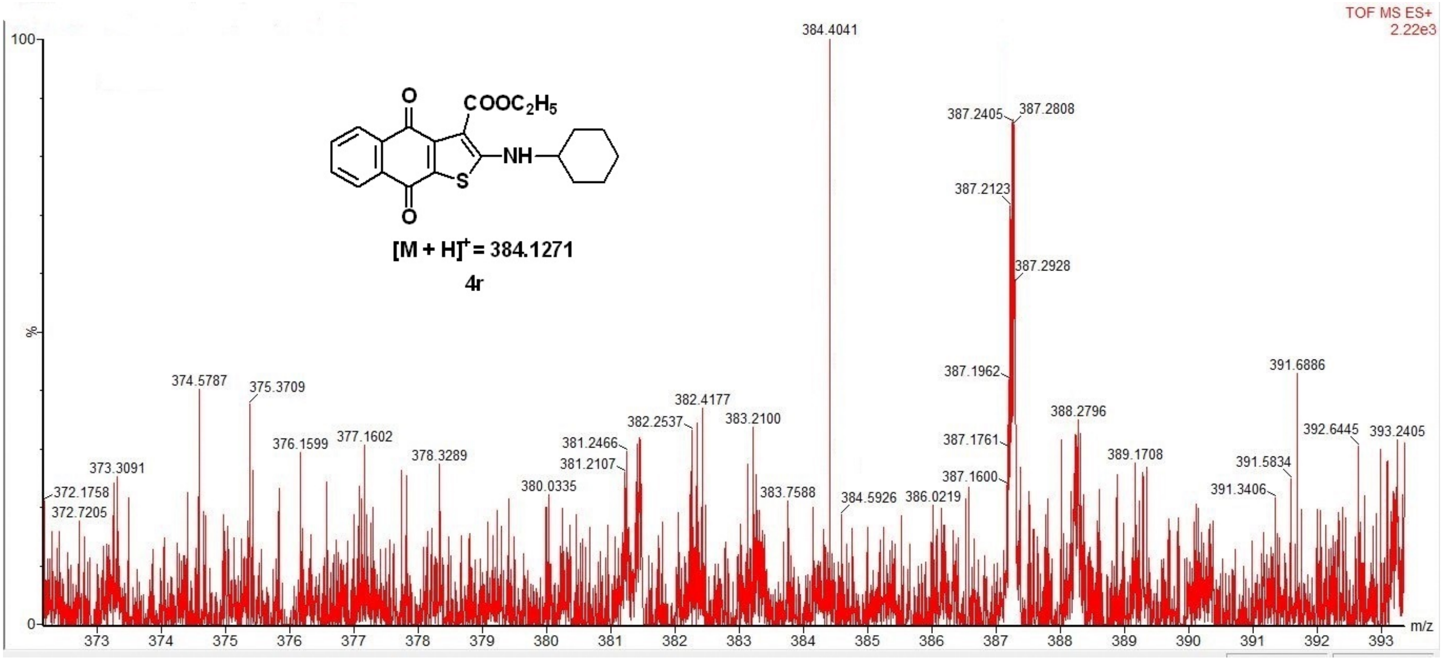
TOF MS ES+
2.22e3
COOC2H5
NH
S
[M + H]+ = 384.1271
4r
384.4041
387.2808
m/z

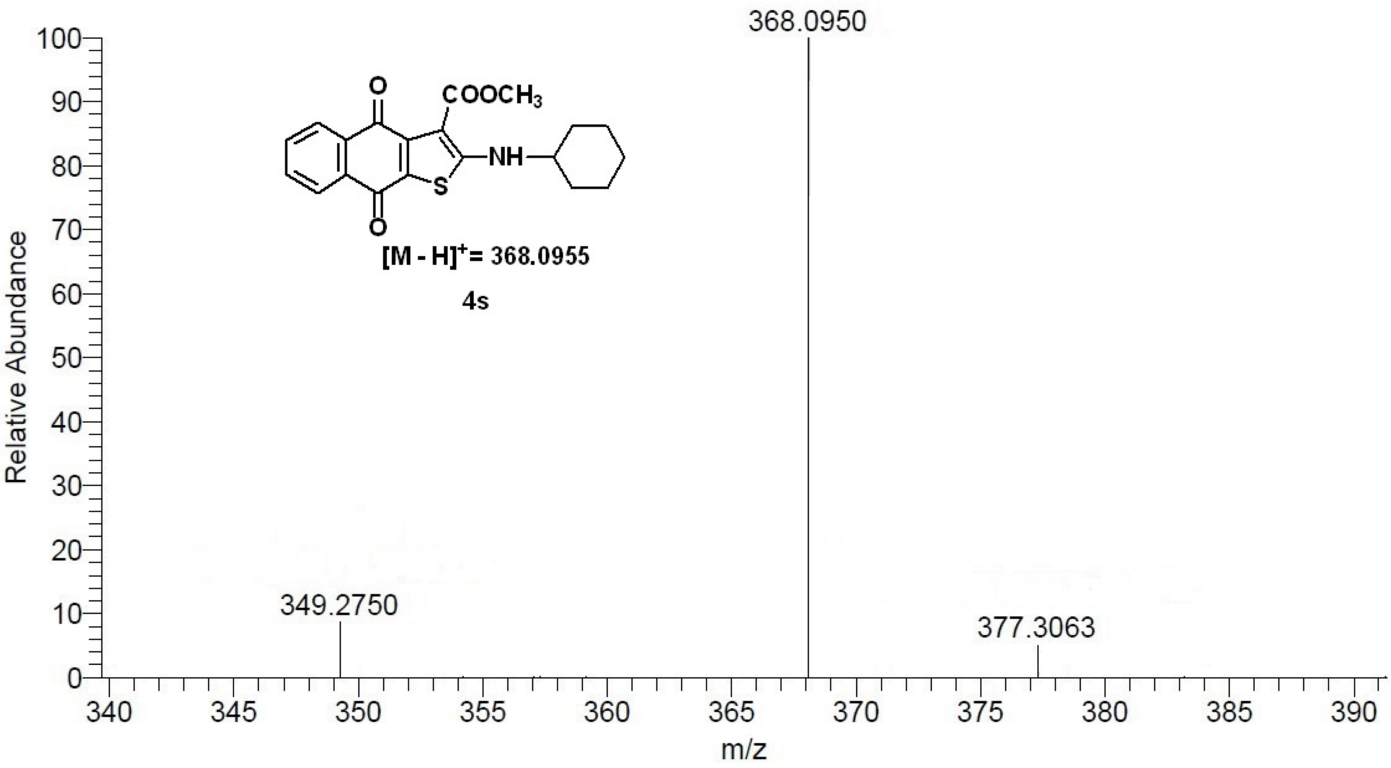
COOCH3
NH
S
[M - H]+ = 368.0955
4s
368.0950
349.2750
377.3063
Relative Abundance
m/z

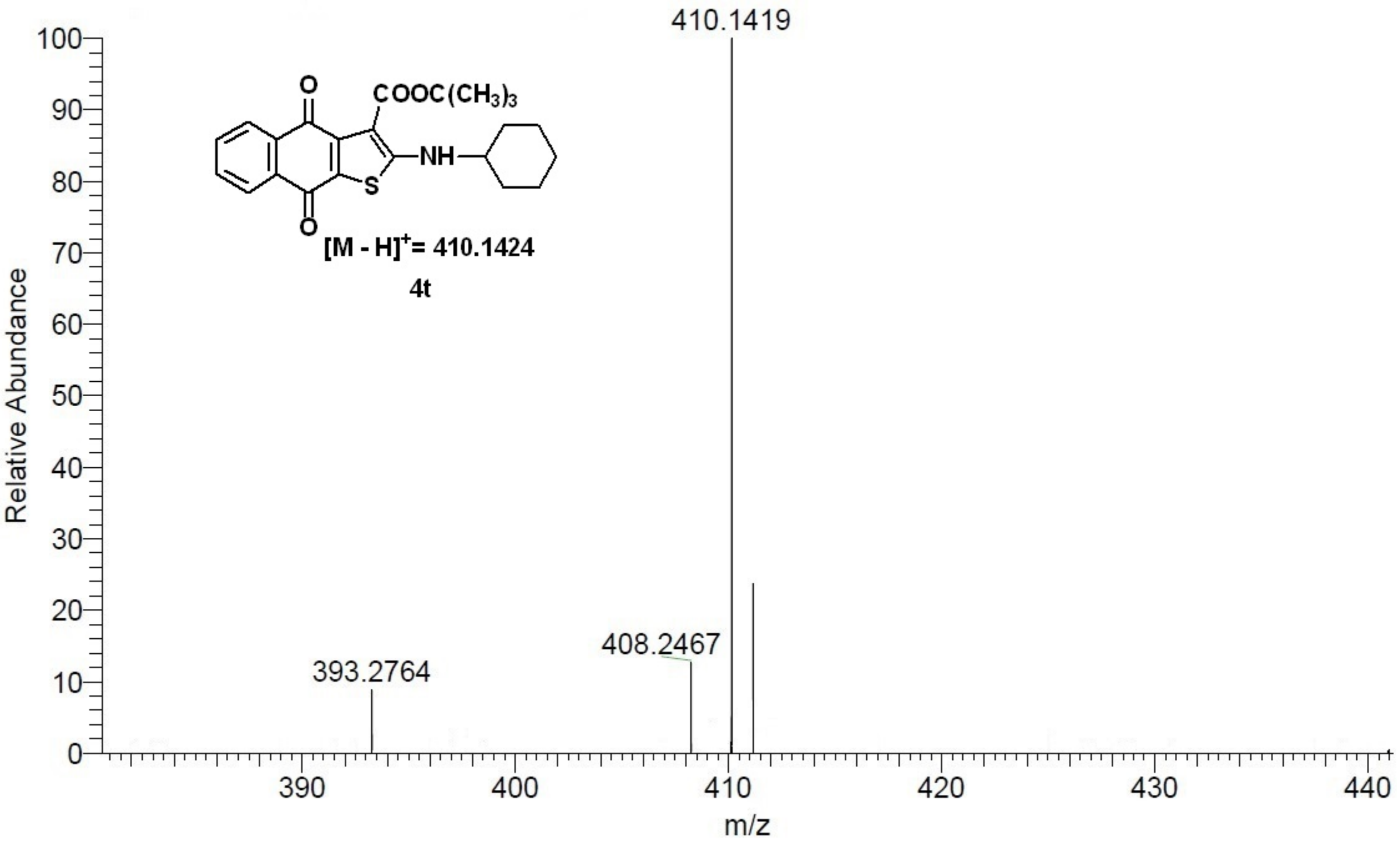
410.1419
COOC(CH3)3
NH
S
O
O
[M - H]+= 410.1424
4t
Relative Abundance
408.2467
393.2764
m/z

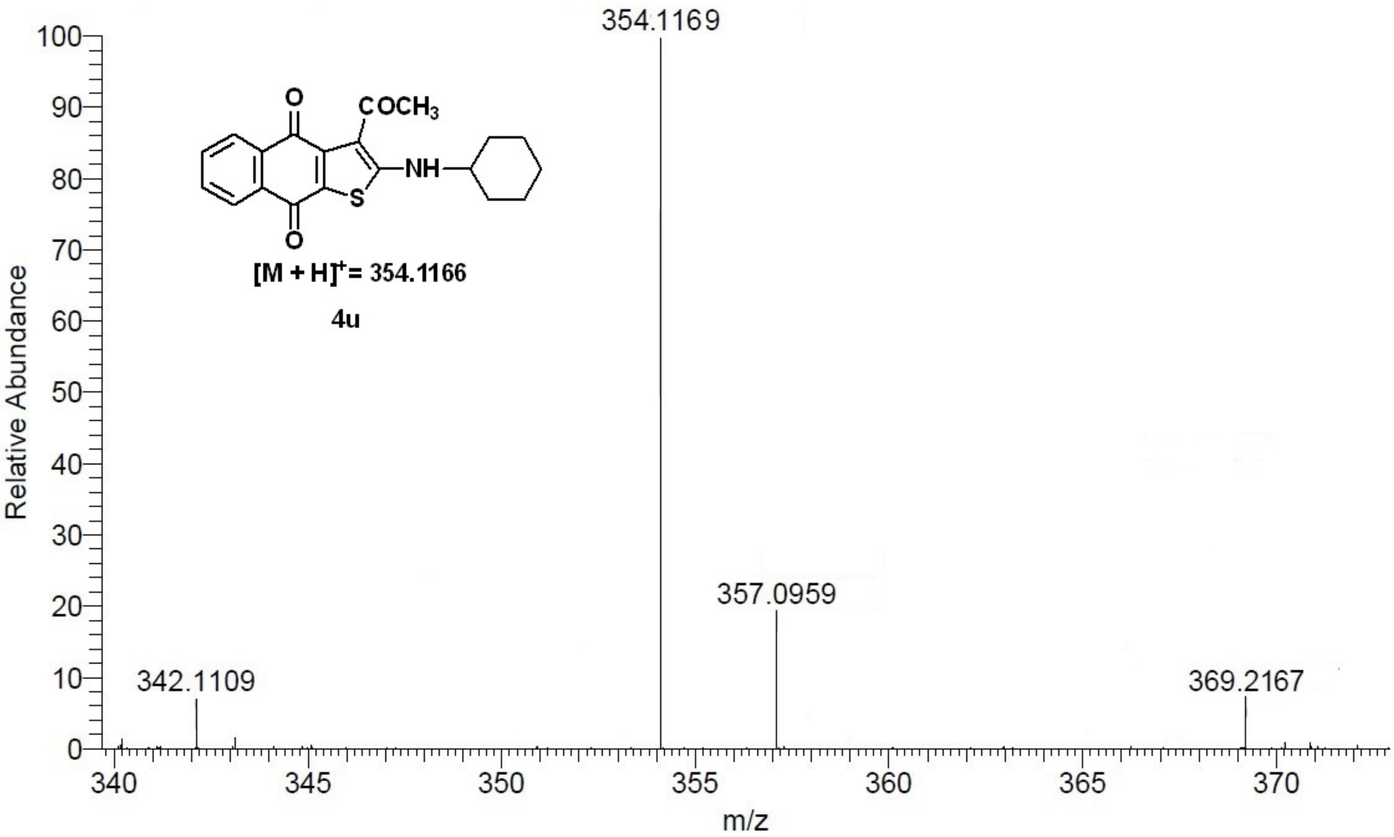
354.1169
COCH3
NH
S
[M + H]+ = 354.1166
4u
357.0959
342.1109
369.2167
Relative Abundance
m/z

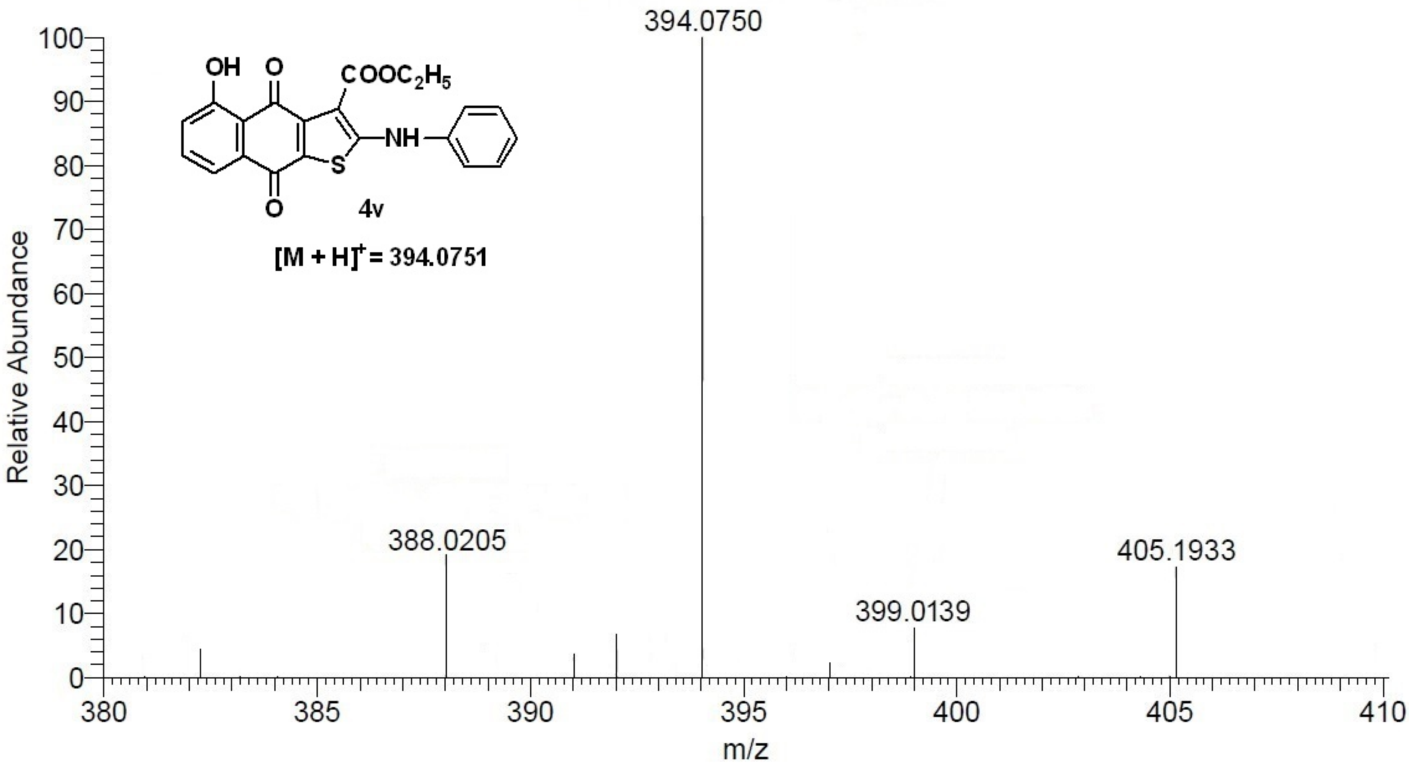
394.0750
OH
COOC2H5
NH
S
4v
[M + H]+ = 394.0751
388.0205
405.1933
399.0139
Relative Abundance
m/z

DFT

The molecular geometry was optimized utilizing Gaussian09W D1 revision. This optimization employed the ωB97XD functional, known for its accuracy in accounting for dispersion interactions, along with the def2SVP basis set, which includes polarization functions to improve the description of electronic distributions in the molecule. For visualization, GaussView5 and Discovery Studio Visualizer 2021 have been used.

Coordinates of the optimized structures are given below.

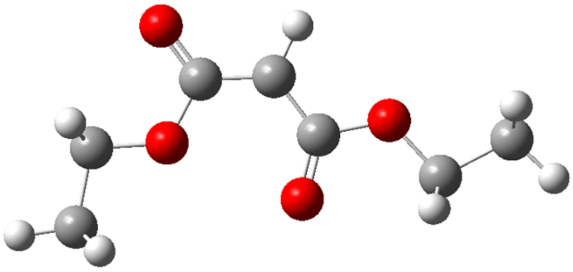

1aa

The sum of electronic and thermal Free Energies = -573.982547

Symbolic Z-matrix:

Charge = -1 Multiplicity = 1

C	0.5656	-0.6766	-0.5316
H	0.0928	-0.3942	-1.4379
C	1.6197	0.1177	-0.0861
O	2.0437	1.1385	-0.6811
O	2.2683	-0.1983	1.0744
C	3.3622	0.5923	1.563
C	3.7848	-0.087	2.8639
H	2.9402	-0.0978	3.5534
H	4.6178	0.4552	3.3083
H	4.0826	-1.1141	2.6516
H	3.029	1.614	1.7505
H	4.1785	0.5861	0.8394
C	0.0867	-1.8115	0.1187
O	0.4868	-2.3242	1.1914
O	-0.9575	-2.4512	-0.4952
C	-1.5318	-3.64	0.0745
C	-2.6303	-4.055	-0.9024

H	-3.3629	-3.2517	-0.9827
H	-3.1162	-4.9612	-0.5436
H	-2.1894	-4.2376	-1.8826
H	-1.9505	-3.4178	1.0572
H	-0.7723	-4.419	0.1553

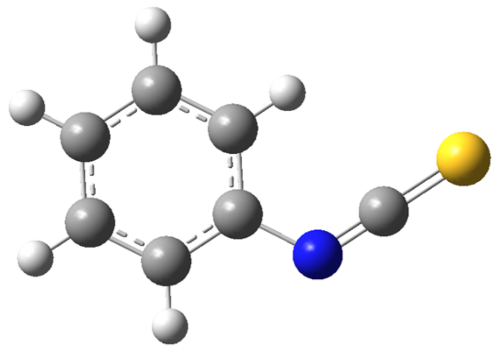

2

The sum of electronic and thermal Free Energies = -722.463620

Symbolic Z-matrix:

Charge = 0 Multiplicity = 1

C	2.90411	-0.98298	-1.71605
N	3.09196	-2.14469	-1.17887
S	2.67665	0.4237	-2.3665
C	5.78003	-4.92741	-2.99203
H	6.45317	-5.62426	-3.44609
C	5.10189	-5.27324	-1.8154
H	5.25727	-6.23414	-1.37108
C	4.22026	-4.36056	-1.22072
H	3.70249	-4.6246	-0.32234
C	4.01675	-3.10205	-1.80266
C	4.69489	-2.75622	-2.97929
H	4.53951	-1.79532	-3.42362
C	5.57652	-3.6689	-3.57398
H	6.09429	-3.40485	-4.47236

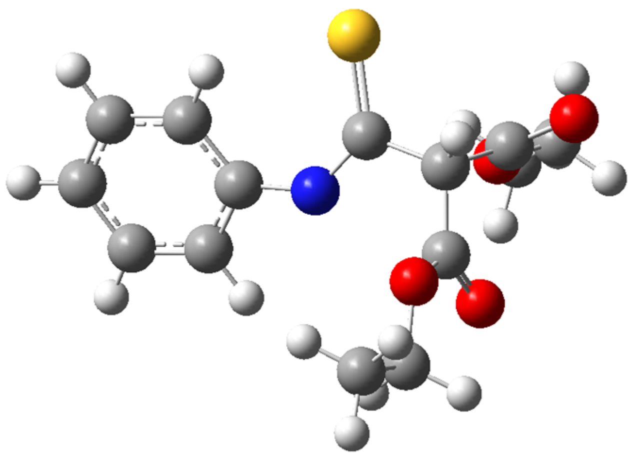

2aa′

The sum of electronic and thermal Free Energies = -1296.465782

Symbolic Z-matrix:

Charge = -1 Multiplicity = 1

C	1.4198	-0.5457	-0.5888
H	0.7818	0.0331	-1.2361
C	2.6135	-1.0285	-1.3843
N	2.9369	-2.3315	-1.2467
S	3.4001	0.1452	-2.3207
C	1.8977	0.2949	0.5589
O	1.3598	1.3705	0.8499
O	2.9224	-0.1332	1.3239
C	3.4175	0.6277	2.4363
C	4.5958	-0.1878	2.9624
H	4.2463	-1.1772	3.2598
H	5.0422	0.3169	3.8179
H	5.3391	-0.299	2.1716
H	2.6448	0.725	3.2008
H	3.7515	1.6114	2.1018
C	0.6154	-1.6812	-0.0107
O	0.5899	-2.0217	1.1803
O	-0.2735	-2.2544	-0.8552
C	-1.027	-3.4116	-0.4475

C	-1.8012	-3.8314	-1.6948
H	-2.4545	-3.0181	-2.011
H	-2.3972	-4.7164	-1.4747
H	-1.0955	-4.0594	-2.495
H	-1.7095	-3.1558	0.3648
H	-0.3467	-4.2076	-0.1363
C	5.8289	-4.8319	-2.9797
H	6.5583	-5.4764	-3.3948
C	5.1774	-5.1734	-1.7981
H	5.4086	-6.0829	-1.3096
C	4.2238	-4.3249	-1.2539
H	3.729	-4.5846	-0.3555
C	3.8912	-3.1014	-1.8616
C	4.5495	-2.7856	-3.0685
H	4.3023	-1.8986	-3.5813
C	5.5049	-3.6341	-3.6112
H	5.9854	-3.3669	-4.5151

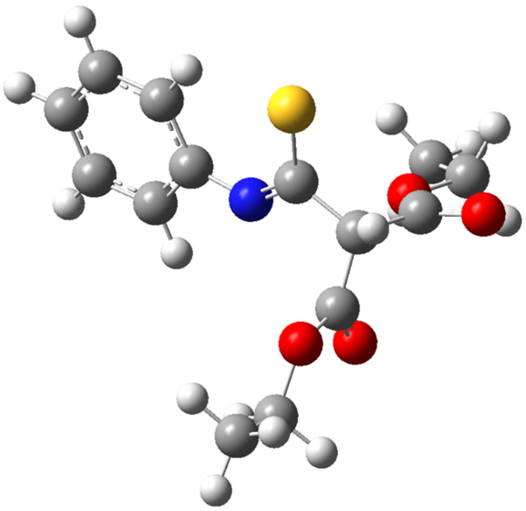

2ab′

The sum of electronic and thermal Free Energies= -1296.466465

Symbolic Z-matrix:

Charge = -1 Multiplicity = 1

C	1.2425	-0.6045	-0.6393

H	0.5649	0.0127	-1.2065
C	2.3389	-1.1001	-1.5466
N	2.9107	-2.2782	-1.2257
S	2.6953	-0.1029	-2.8638
C	1.8423	0.1789	0.4895
O	1.3119	1.2079	0.9264
O	2.9477	-0.2782	1.1157
C	3.5576	0.4333	2.2042
C	4.7559	-0.4266	2.5989
H	4.4074	-1.4132	2.9061
H	5.2942	0.0452	3.4199
H	5.4195	-0.5367	1.7399
H	2.858	0.5238	3.0369
H	3.8861	1.4198	1.8716
C	0.4812	-1.7557	-0.0465
O	0.5989	-2.1905	1.1064
O	-0.4834	-2.2866	-0.8303
C	-1.2593	-3.4193	-0.3917
C	-2.2079	-3.7139	-1.5516
H	-2.8376	-2.8437	-1.7384
H	-2.8337	-4.5707	-1.3044
H	-1.6265	-3.9349	-2.4476
H	-1.8209	-3.17	0.5105
H	-0.6048	-4.2735	-0.2064
C	5.9764	-4.686	-2.7967
H	6.743	-5.3129	-3.1726
C	5.2347	-5.0687	-1.6835
H	5.4362	-5.9923	-1.206
C	4.231	-4.2441	-1.1945
H	3.6711	-4.5366	-0.3457
C	3.9244	-3.0081	-1.7962

C	4.6956	-2.6423	-2.9215
H	4.5142	-1.7236	-3.4054
C	5.6997	-3.4675	-3.4103
H	6.2578	-3.1624	-4.2571

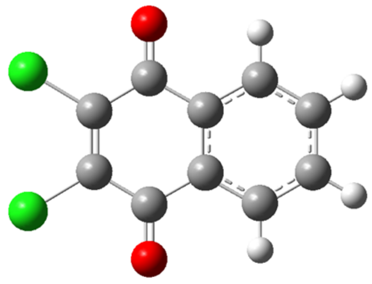

3A

The sum of electronic and thermal Free Energies= -1454.000719

Symbolic Z-matrix:

Charge = 0 Multiplicity = 1

C	1.2579	0.6989	-0.059
C	0.0349	1.3854	-0.1194
H	0.0272	2.4441	-0.2038
C	-1.1687	0.6947	-0.0703
H	-2.0874	1.2244	-0.1171
C	-1.172	-0.693	0.0404
H	-2.0934	-1.219	0.0779
C	0.0283	-1.3885	0.1018
H	0.0157	-2.4471	0.1864
C	1.2546	-0.7067	0.053
C	2.5282	1.4155	-0.1111
C	2.5217	-1.4281	0.1154
C	3.7635	-0.6926	0.058
Cl	5.2537	-1.5849	0.1309
C	3.7666	0.6746	-0.0512
Cl	5.2607	1.5606	-0.1208
O	2.5328	2.6395	-0.2052
O	2.5209	-2.6516	0.2156

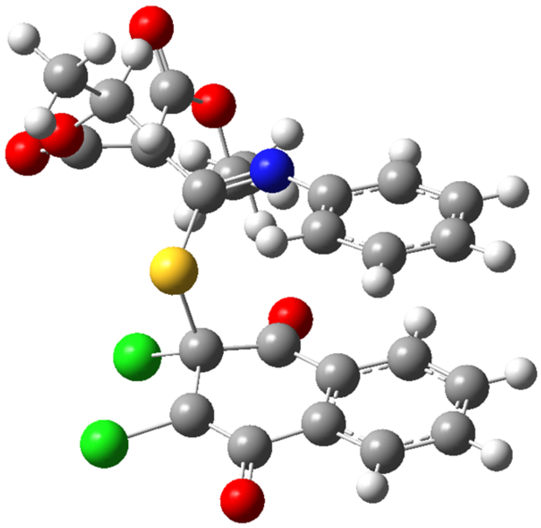

3AA′

The sum of electronic and thermal Free Energies = -2750.410582

Symbolic Z-matrix:

Charge = -1 Multiplicity = 1

C	0.6793	-1.5701	0.9134
H	0.2499	-0.6979	1.3778
C	1.1946	-1.2069	-0.4542
N	2.4401	-1.2915	-0.7411
S	0.0126	-0.6517	-1.6003
C	1.7591	-2.1575	1.7876
O	2.0316	-3.3539	1.8189
O	2.4917	-1.3744	2.6047
C	2.4878	0.0635	2.7116
C	3.2944	0.3514	3.9746
H	2.8075	-0.1096	4.8348
H	3.3592	1.4282	4.1302
H	4.2991	-0.0598	3.8693
H	1.4763	0.4576	2.8230
H	2.9870	0.5048	1.8478
C	-0.3899	-2.6163	0.7633
O	-1.4532	-2.4764	1.3598
O	-0.3041	-3.7134	-0.0281

C	0.7584	-4.2164	-0.8803
C	0.2399	-5.5642	-1.3728
H	0.0591	-6.2183	-0.5191
H	0.9798	-6.0232	-2.0281
H	-0.6924	-5.4215	-1.9201
H	1.6864	-4.3615	-0.3265
H	0.9215	-3.5576	-1.7331
C	4.4731	-0.3888	-4.3270
H	4.9946	-0.1433	-5.2172
C	5.0214	-0.0774	-3.0875
H	5.9656	0.4018	-3.0319
C	4.3324	-0.3945	-1.9220
H	4.7544	-0.1622	-0.9787
C	3.0846	-1.0175	-1.9747
C	2.5601	-1.3601	-3.2281
H	1.6446	-1.8841	-3.3021
C	3.2425	-1.0332	-4.3948
H	2.8268	-1.2817	-5.3388
C	1.9420	2.2636	-3.2171
C	0.6489	1.7868	-3.6716
C	-0.5104	1.8701	-2.6929
H	-0.7559	2.9035	-2.4938
Cl	-1.9830	1.0611	-3.3644
C	-0.0719	1.1694	-1.3912
Cl	-1.3485	1.4194	-0.1576
C	1.2558	1.7488	-0.8885
C	2.2245	2.2861	-1.8365
O	0.4998	1.3744	-4.7989
O	1.5022	1.7305	0.3018
C	3.4478	2.8162	-1.3985
H	3.6698	2.8505	-0.3608

C	4.3819	3.2889	-2.3123
H	5.3057	3.6857	-1.9713
C	4.1129	3.2347	-3.6775
H	4.8332	3.5856	-4.3739
C	2.9039	2.7206	-4.1281
H	2.7052	2.6794	-5.1707

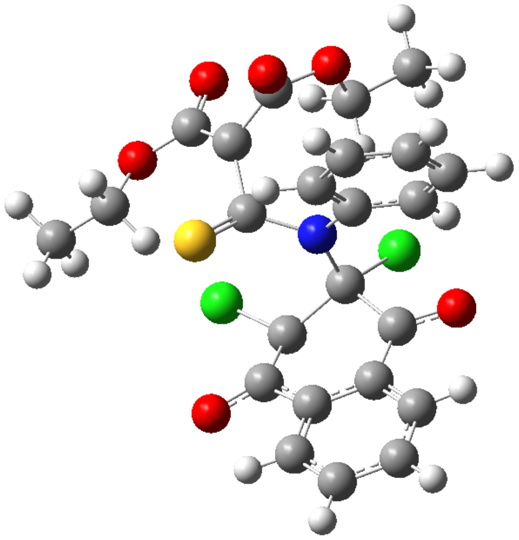

3AA''

The sum of electronic and thermal Free Energies = -2750.348469

Symbolic Z-matrix:

Charge = -1 Multiplicity = 1

C	1.537	-2.624	-0.155
C	1.514	-1.462	-1.284
N	2.228	-0.125	-0.974
S	1.437	-2.018	-2.771
C	2.813	-2.853	0.594
O	3.759	-3.347	-0.044
O	3.248	-2.231	1.807
C	2.313	-1.238	2.322
C	2.85	-0.679	3.682
H	2.914	-1.501	4.404
H	2.165	0.088	4.061

H	3.859	-0.224	3.56
H	1.324	-1.71	2.463
H	2.226	-0.429	1.614
C	0.233	-3.222	0.501
O	0.322	-3.516	1.698
O	-0.998	-3.475	-0.134
C	-1.062	-3.38	-1.533
C	-2.478	-3.585	-2.007
H	-2.837	-4.589	-1.722
H	-2.481	-3.478	-3.077
H	-3.122	-2.812	-1.561
H	-0.39	-4.111	-1.988
H	-0.746	-2.385	-1.82
C	6.405	-0.457	-1.155
H	7.42	-0.544	-1.213
C	5.87	0.568	-0.394
H	6.538	1.254	0.126
C	4.49	0.701	-0.309
H	4.069	1.488	0.273
C	3.642	-0.176	-0.989
C	4.2	-1.199	-1.734
H	3.524	-1.876	-2.227
C	5.577	-1.352	-1.823
H	5.992	-2.154	-2.398
C	1.252	2.128	-3.65
C	0.229	1.24	-3.245
C	0.031	1.092	-1.658
Cl	-1.179	-0.178	-1.228
C	1.434	1.17	-0.749
Cl	1.346	1.718	1.05
C	2.286	2.411	-1.342

C	2.192	2.702	-2.798
O	-0.59	0.881	-4.178
O	3.228	3.015	-0.652
C	3.111	3.59	-3.328
H	3.803	4.011	-2.681
C	3.109	3.907	-4.687
H	3.826	4.594	-5.079
C	2.167	3.323	-5.53
H	2.144	3.556	-6.585
C	1.243	2.437	-4.992
H	0.515	1.983	-5.571

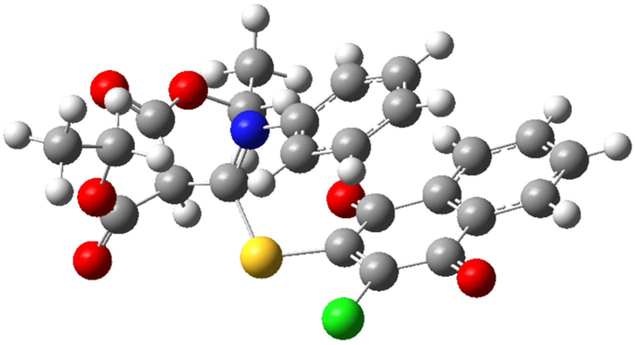

3AB’

The sum of electronic and thermal Free Energies = -2290.314325

Symbolic Z-matrix:

Charge = 0 Multiplicity = 1

C	0.9782	-1.8884	1.2074
H	0.5369	-1.0851	1.7757
C	1.3138	-1.3836	-0.1660
N	2.4664	-1.5838	-0.6984
S	0.0027	-0.5115	-0.9341
C	2.1900	-2.4127	1.9322
O	2.4538	-3.6056	2.0442
O	3.0509	-1.5653	2.5313
C	3.0671	-0.1233	2.4618
C	4.0534	0.2988	3.5468

H	3.7001	-0.0462	4.5193
H	4.1417	1.3850	3.5582
H	5.0308	-0.1413	3.3444
H	2.0846	0.3037	2.6691
H	3.4333	0.2000	1.4861
C	-0.0263	-2.9968	1.0667
O	-1.0817	-2.9309	1.6894
O	0.0940	-4.0611	0.2384
C	1.1588	-4.4813	-0.6515
C	0.7764	-5.9025	-1.0536
H	0.7280	-6.5304	-0.1629
H	1.5251	-6.3033	-1.7365
H	-0.1981	-5.8974	-1.5430
H	2.1280	-4.4914	-0.1520
H	1.1940	-3.8492	-1.5377
C	3.7136	-0.3136	-4.5091
H	4.0251	0.0293	-5.4639
C	4.4396	0.0305	-3.3727
H	5.3097	0.6309	-3.4617
C	4.0282	-0.4189	-2.1225
H	4.5844	-0.1665	-1.2567
C	2.8781	-1.1985	-1.9922
C	2.1729	-1.5676	-3.1425
H	1.3154	-2.1832	-3.0636
C	2.5849	-1.1197	-4.3935
H	2.0388	-1.3919	-5.2618
C	2.1958	2.8633	-3.2012
C	1.1192	2.1180	-3.8465
C	0.4067	1.1106	-3.1002
Cl	-0.8483	0.2169	-3.9059
C	0.7317	0.8438	-1.7924

C	1.6974	1.6611	-1.0934
C	2.4776	2.6426	-1.8374
O	0.8358	2.3501	-5.0198
O	1.8538	1.5377	0.1212
C	3.5111	3.3657	-1.2222
H	3.7275	3.2086	-0.1949
C	4.2574	4.2868	-1.9453
H	5.0378	4.8287	-1.4716
C	3.9822	4.5025	-3.2928
H	4.5535	5.2075	-3.8437
C	2.9601	3.7984	-3.9158
H	2.7560	3.9714	-4.9436

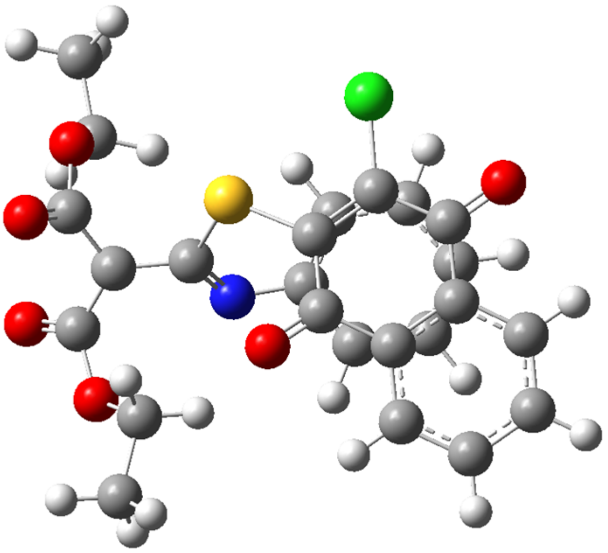

3AC′

The sum of electronic and thermal Free Energies = -2290.307023

Symbolic Z-matrix:

Charge = -1 Multiplicity = 2

C	1.3009	-2.1215	1.0499
C	1.5677	-1.44	-0.1636
N	2.7833	-1.3705	-0.6491
S	0.188	-0.7375	-1.0025
C	2.296	-2.3792	2.0069
O	2.4996	-3.4892	2.5159

O	3.1061	-1.3953	2.489
C	2.8069	-0.0024	2.3059
C	3.5387	0.7101	3.4407
H	3.164	0.3411	4.3957
H	3.3616	1.7821	3.3671
H	4.6064	0.5053	3.367
H	1.7311	0.1732	2.3761
H	3.1856	0.3361	1.3422
C	0.0258	-2.648	1.2943
O	-0.7157	-2.323	2.2289
O	-0.4763	-3.6054	0.4659
C	0.4238	-4.4467	-0.2827
C	-0.4715	-5.5037	-0.9248
H	-0.9962	-6.0528	-0.1427
H	0.1375	-6.1918	-1.5091
H	-1.1995	-5.0155	-1.5729
H	1.1484	-4.91	0.3912
H	0.9417	-3.8716	-1.0505
C	3.9404	-0.1442	-4.4898
H	4.2268	0.1852	-5.4564
C	4.5838	0.3553	-3.3605
H	5.3677	1.0611	-3.4659
C	4.2031	-0.0753	-2.0947
H	4.6961	0.2914	-1.2318
C	3.16	-0.9926	-1.9333
C	2.5465	-1.5164	-3.0794
H	1.7836	-2.2429	-2.9704
C	2.9269	-1.0878	-4.3462
H	2.4441	-1.4811	-5.2046
C	2.1347	2.7951	-3.2161
C	1.1519	1.954	-3.8905

C	0.4955	0.9034	-3.1541
Cl	-0.6017	-0.1381	-4.0132
C	0.754	0.7145	-1.8169
C	1.5253	1.6959	-1.0843
C	2.3188	2.6676	-1.8256
O	0.9023	2.1476	-5.0812
O	1.4846	1.7663	0.1398
C	3.2661	3.4765	-1.1794
H	3.408	3.3837	-0.1311
C	4.0213	4.3941	-1.898
H	4.736	5.0002	-1.3998
C	3.8399	4.5206	-3.2728
H	4.4164	5.2235	-3.8206
C	2.9043	3.7292	-3.9267
H	2.7711	3.832	-4.975

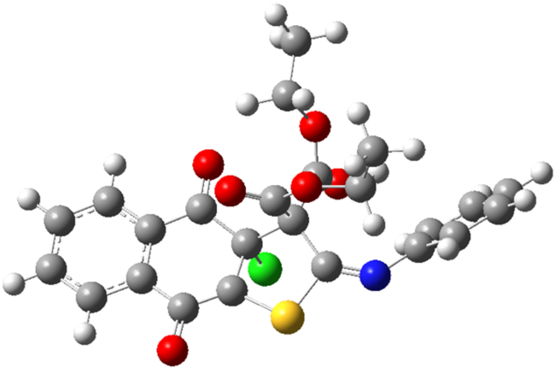

3AD′

The sum of electronic and thermal Free Energies = -2290.266960

Symbolic Z-matrix:

Charge = -1 Multiplicity = 1

C	2.4304	-0.1019	-1.3775
C	1.7213	-0.5977	-2.6497
N	1.8281	-1.6353	-3.4255
S	0.4704	0.534	-3.0716
C	3.8133	-0.6655	-1.0776
O	4.7272	-0.6388	-1.8973

O	4.2252	-1.1647	0.123
C	3.7186	-1.1823	1.4771
C	4.9112	-1.6127	2.327
H	5.2512	-2.598	2.0077
H	4.6154	-1.6503	3.3748
H	5.7207	-0.8923	2.2043
H	2.9148	-1.9084	1.5877
H	3.3909	-0.1865	1.78
C	1.4713	-0.3975	-0.2264
O	1.1494	0.4287	0.6318
O	0.9951	-1.6333	0.0611
C	1.2181	-2.8922	-0.57
C	0.8053	-3.9313	0.4691
H	1.4277	-3.8285	1.3579
H	0.9341	-4.9296	0.0514
H	-0.2406	-3.7824	0.7389
H	2.2715	-3.0199	-0.8221
H	0.5777	-2.9749	-1.4473
C	4.4324	-4.9382	-3.7136
H	5.086	-5.7692	-3.7767
C	4.9129	-3.6534	-3.947
H	5.9313	-3.5031	-4.1965
C	4.0561	-2.5612	-3.8578
H	4.4161	-1.5897	-4.0755
C	2.7181	-2.7289	-3.4961
C	2.2354	-4.0304	-3.3211
H	1.2068	-4.1794	-3.1264
C	3.0874	-5.1261	-3.4113
H	2.7109	-6.1037	-3.2559
C	2.3094	3.5956	-0.178
C	2.9372	2.2997	-0.4044

C	2.4767	1.4484	-1.5929
Cl	3.5557	1.8957	-2.9697
C	1.1016	1.8453	-2.0906
C	0.4748	3.0299	-1.7981
C	1.1324	3.9598	-0.8761
O	3.8681	1.9239	0.2932
O	-0.6998	3.3522	-2.2971
C	0.5888	5.2364	-0.6475
H	-0.2881	5.5227	-1.1695
C	1.1713	6.1214	0.2501
H	0.7403	7.0767	0.4062
C	2.3165	5.7524	0.9475
H	2.7585	6.4245	1.6372
C	2.8779	4.5018	0.7357
H	3.752	4.225	1.2676

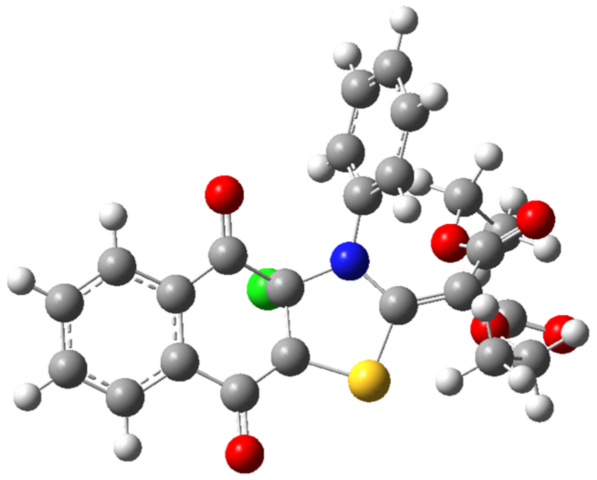

3AD''

The sum of electronic and thermal Free Energies= -2290.176483

Symbolic Z-matrix:

Charge = -1 Multiplicity = 1

C	1.9178	-2.1389	0.2025
C	1.7462	-0.8968	-0.4252
N	2.2294	-0.5678	-1.6881

S	0.9131	0.4307	0.2846
C	2.3437	-2.2009	1.5518
O	2.0025	-3.054	2.3883
O	3.2284	-1.2574	1.997
C	3.678	-1.1985	3.3654
C	4.7072	-0.0708	3.3926
H	5.5264	-0.3084	2.7136
H	5.0938	0.047	4.4043
H	4.2341	0.8591	3.0729
H	4.142	-2.143	3.6542
H	2.8407	-0.9664	4.0254
C	1.6623	-3.359	-0.4755
O	2.153	-4.4635	-0.1882
O	0.786	-3.3527	-1.5275
C	0.5491	-4.4726	-2.4071
C	-0.6665	-5.1976	-1.8292
H	-0.4347	-5.5542	-0.8248
H	-0.9192	-6.0462	-2.4638
H	-1.5125	-4.511	-1.7837
H	1.4161	-5.1345	-2.4469
H	0.3221	-4.1042	-3.4079
C	5.0954	-3.0246	-3.5176
H	5.8164	-3.6549	-3.969
C	5.3625	-2.4339	-2.2827
H	6.2855	-2.6152	-1.797
C	4.4174	-1.6054	-1.6864
H	4.6108	-1.1486	-0.7505
C	3.2006	-1.3762	-2.3311
C	2.9283	-1.9601	-3.5689
H	2.0017	-1.7796	-4.0454
C	3.8803	-2.7854	-4.1595

H	3.68	-3.232	-5.098
C	2.2663	2.96	-3.229
C	2.5552	1.5752	-3.1919
C	1.6683	0.6895	-2.2713
Cl	0.2208	0.223	-3.2289
C	1.1316	1.4763	-1.0973
C	0.9084	2.8301	-1.1255
C	1.3929	3.5813	-2.2687
O	3.4307	1.0768	-4.0001
O	0.3237	3.4705	-0.1381
C	1.0676	4.937	-2.4234
H	0.4048	5.3857	-1.7284
C	1.6026	5.7067	-3.4464
H	1.3435	6.7302	-3.5358
C	2.4987	5.1253	-4.3542
H	2.9221	5.714	-5.1267
C	2.8275	3.7931	-4.2472
H	3.504	3.3695	-4.9451

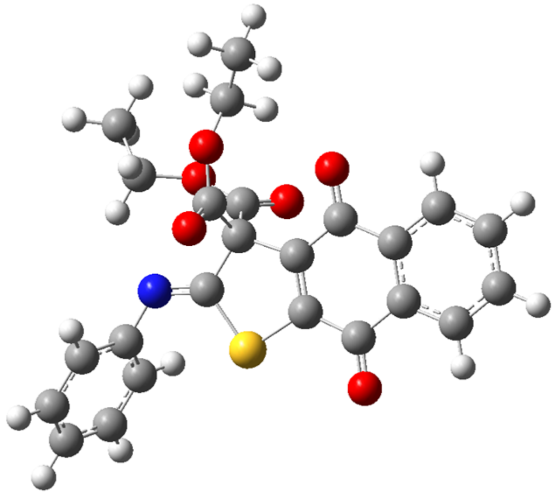

4′a

The sum of electronic and thermal Free Energies = -1830.233465

Symbolic Z-matrix:

Charge = 0 Multiplicity = 1

C	2.0448	0.2573	-1.5359
C	1.7950	0.0161	-3.0141
N	2.0568	-1.0840	-3.6096
S	1.1209	1.4029	-3.8309
C	3.5032	-0.0103	-1.2301
O	4.3711	0.3391	-2.0270
O	3.9848	-0.5429	-0.0789
C	3.4114	-0.9865	1.1820
C	4.4840	-0.6625	2.2174
H	5.4083	-1.1844	1.9674
H	4.1458	-0.9755	3.2049
H	4.6674	0.4130	2.2254
H	3.2333	-2.0614	1.1643
H	2.4916	-0.4581	1.4326
C	1.0959	-0.6438	-0.7755
O	0.1215	-0.1698	-0.1948
O	1.1928	-1.9962	-0.6943
C	2.0731	-2.9874	-1.2938
C	1.8818	-4.2359	-0.4364
H	2.1595	-4.0300	0.5967
H	2.5051	-5.0424	-0.8230
H	0.8362	-4.5450	-0.4702
H	3.1180	-2.6764	-1.2747
H	1.7613	-3.2110	-2.3156
C	1.4907	3.8518	0.0051
C	1.8834	2.4459	-0.0928
C	1.7123	1.7426	-1.3250
C	1.2129	2.3864	-2.4125
C	0.8043	3.7812	-2.3781
C	0.9566	4.5111	-1.1245
O	2.3709	1.8722	0.8709

O	0.3460	4.3215	-3.3744
C	0.5830	5.8601	-1.0209
H	0.1803	6.3612	-1.8661
C	0.7354	6.5467	0.1764
H	0.4500	7.5670	0.2441
C	1.2627	5.8977	1.2903
H	1.3778	6.4251	2.2043
C	1.6370	4.5627	1.2065
H	2.0375	4.0765	2.0612
C	1.3666	-1.7404	-7.7047
H	1.2006	-1.9071	-8.7385
C	2.6630	-1.7490	-7.1968
H	3.4846	-1.9238	-7.8437
C	2.8802	-1.5276	-5.8409
H	3.8648	-1.5350	-5.4505
C	1.8061	-1.2901	-4.9834
C	0.5063	-1.2937	-5.4972
H	-0.3151	-1.1298	-4.8499
C	0.2883	-1.5142	-6.8533
H	-0.6991	-1.5108	-7.2396

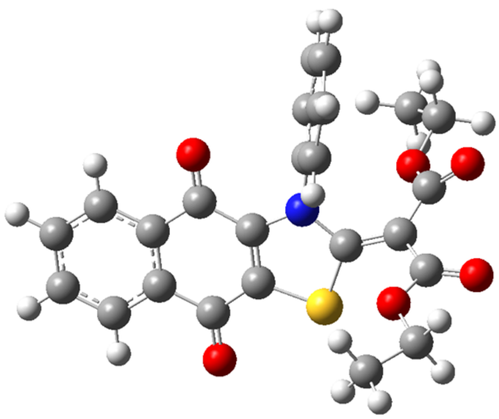

4’ab

The sum of electronic and thermal Free Energies = -1830.088163

Symbolic Z-matrix:

Charge = 0 Multiplicity = 1

C	1.9342	-2.1178	0.1431
C	1.8377	-0.9267	-0.5942
N	2.5267	-0.6542	-1.7614
S	0.8611	0.4326	-0.1703
C	2.278	-2.0681	1.5151
O	2.0411	-2.9305	2.3652
O	2.9212	-0.9502	1.9626
C	3.292	-0.7861	3.3514
C	3.9424	0.5935	3.418
H	4.8193	0.6171	2.7705
H	4.2446	0.8045	4.4438
H	3.2294	1.3503	3.0887
H	4.0021	-1.5598	3.6516
H	2.4065	-0.821	3.9897
C	1.6789	-3.3723	-0.4631
O	2.1063	-4.4671	-0.0859
O	0.8886	-3.3895	-1.5778
C	0.6217	-4.62	-2.292
C	-0.3317	-4.2232	-3.4166
H	-1.2414	-3.7975	-2.9921
H	-0.5868	-5.1048	-4.0045
H	0.1458	-3.4858	-4.0617
H	0.1485	-5.3499	-1.6312
H	1.5478	-5.0292	-2.7014
C	5.3625	-3.4149	-3.1346
H	6.0744	-4.1255	-3.4703
C	5.6346	-2.6395	-2.0069
H	6.5522	-2.7627	-1.4901
C	4.7042	-1.7059	-1.5617

H	4.904	-1.1114	-0.7078
C	3.5032	-1.5674	-2.2553
C	3.2224	-2.3277	-3.3884
H	2.3089	-2.1969	-3.907
C	4.1606	-3.2568	-3.8257
H	3.9643	-3.8433	-4.6865
C	2.3535	2.4955	-3.9639
C	2.7892	1.156	-3.5601
C	2.2408	0.6042	-2.3359
C	1.3522	1.304	-1.5774
C	0.9006	2.6233	-1.9418
C	1.4259	3.2148	-3.1707
O	3.5899	0.5445	-4.2579
O	0.0991	3.2323	-1.2427
C	1.0204	4.4948	-3.5799
H	0.3252	5.0407	-2.9911
C	1.5183	5.0575	-4.7476
H	1.2037	6.0262	-5.0465
C	2.4308	4.3524	-5.5275
H	2.8102	4.785	-6.4194
C	2.8449	3.0847	-5.14
H	3.5419	2.5567	-5.7429

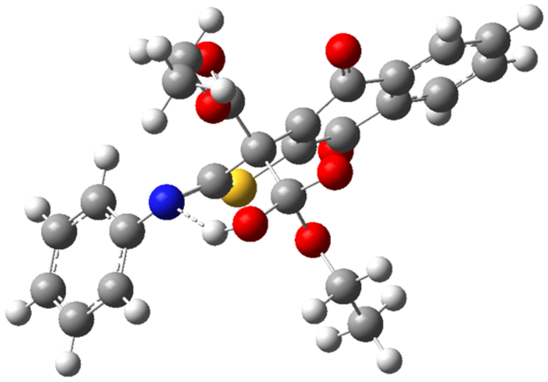

4'aa

The sum of electronic and thermal Free Energies = -1905.925134

Symbolic Z-matrix:

Charge = 0 Multiplicity = 1

C	-3.2810	-2.1550	-1.1340
C	-3.5820	-0.8140	-0.8100
C	-4.8700	-0.3200	-1.0670
C	-5.8450	-1.1380	-1.6240
C	-5.5510	-2.4640	-1.9350
C	-4.2800	-2.9690	-1.6920
C	-1.9400	-2.6710	-0.8820
C	-0.9180	-1.7290	-0.4620
C	-1.2000	-0.4240	-0.2070
C	-2.5580	0.0320	-0.2020
O	-1.6860	-3.8640	-1.0180
O	-2.9030	1.0490	0.3980
S	0.7430	-2.1580	-0.2180
C	1.1830	-0.5120	0.1820
C	0.0280	0.4550	0.0170
N	2.3700	-0.1680	0.5430
C	3.4140	-1.1050	0.6840
C	4.5260	-1.0180	-0.1540
C	5.5750	-1.9240	-0.0280
C	5.5210	-2.9210	0.9430
C	4.4190	-3.0080	1.7900
C	3.3710	-2.1000	1.6630
C	0.3030	1.2650	-1.2780
O	0.4760	0.3450	-2.3680
O	1.4680	2.0700	-1.2030
O	-0.7910	2.0860	-1.6050
C	-0.0990	1.2430	1.3020
O	-0.4590	0.6930	2.3560
O	0.3240	2.5210	1.3920
C	0.7250	4.6690	2.2370
C	0.1760	3.2910	2.5950

C	0.8060	-0.2760	-4.6020
C	0.7190	0.9240	-3.6630
H	-5.0950	0.6910	-0.8330
H	-6.8140	-0.7510	-1.8140
H	-6.2980	-3.0870	-2.3590
H	-4.0590	-3.9790	-1.9320
H	4.5610	-0.2580	-0.8900
H	6.4130	-1.8550	-0.6720
H	6.3190	-3.6130	1.0380
H	4.3770	-3.7640	2.5310
H	2.5350	-2.1590	2.3100
H	2.1650	1.5410	-0.7870
H	0.6400	5.3340	3.0960
H	0.1550	5.0750	1.4000
H	1.7700	4.5780	1.9420
H	0.7530	2.8450	3.4060
H	-0.8790	3.3590	2.8660
H	0.9900	0.0690	-5.6170
H	-0.1340	-0.8270	-4.5640
H	1.6190	-0.9270	-4.2790
H	1.6530	1.4870	-3.6660
H	-0.1100	1.5760	-3.9430

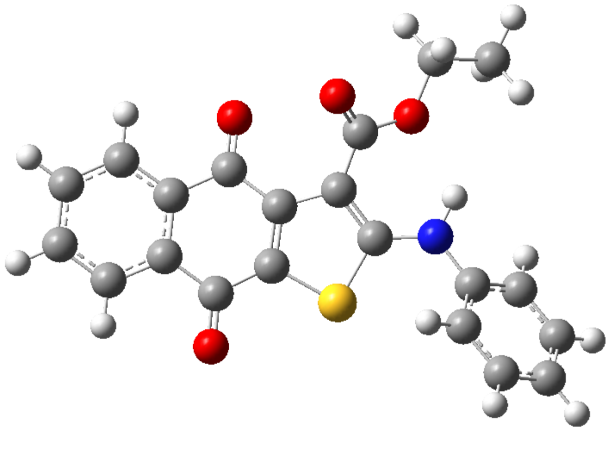

4a

The sum of electronic and thermal Free Energies = -1562.529165

Symbolic Z-matrix:

Charge = 0 Multiplicity = 1

C	-3.3260	-2.1064	-1.4629
C	-3.6978	-0.8353	-0.9644
C	-5.0421	-0.4373	-1.0429
H	-5.3320	0.5153	-0.6728
C	-5.9994	-1.2749	-1.6006
H	-7.0123	-0.9619	-1.6535
C	-5.6325	-2.5248	-2.0920
H	-6.3666	-3.1620	-2.5187
C	-4.3081	-2.9374	-2.0250
H	-4.0372	-3.8920	-2.4033
C	-1.9316	-2.5351	-1.3875
C	-0.9987	-1.6697	-0.7194
C	-1.3473	-0.4541	-0.1612
C	-2.6862	0.0524	-0.3859
O	-1.5768	-3.6054	-1.8737
O	-2.9603	1.2244	-0.1719
S	0.6893	-2.0146	-0.5180
C	0.9044	-0.5524	0.3715
C	-0.2695	0.1714	0.5298
N	2.1606	-0.1687	0.8482
H	2.2746	0.8295	1.0980
C	3.3199	-0.9640	0.8526
C	4.5099	-0.3978	0.3960
H	4.5213	0.5977	0.0362
C	5.6761	-1.1557	0.4085
H	6.5844	-0.7362	0.0583
C	5.6534	-2.4682	0.8787
H	6.5435	-3.0439	0.8849
C	4.4618	-3.0251	1.3415
H	4.4490	-4.0222	1.7010
C	3.2909	-2.2745	1.3339

H	2.3862	-2.6886	1.6952
C	-0.3576	1.3502	1.3581
O	-1.2816	1.6066	2.1215
O	0.6930	2.2190	1.3477
C	2.0284	4.0850	1.8756
H	2.0939	5.0188	2.4342
H	2.1330	4.2962	0.8108
H	2.8307	3.4195	2.1952
C	0.6755	3.4285	2.1401
H	0.5673	3.1910	3.2008
H	-0.1377	4.0825	1.8171
